# The Immune Barrier of Porcine Uterine Mucosa Differs Dramatically at Proliferative and Secretory Phases and Could Be Positively Modulated by Colonizing Microbiota

**DOI:** 10.3389/fimmu.2021.750808

**Published:** 2021-11-30

**Authors:** Deping Han, Peng Sun, Yanxin Hu, Jing Wang, Guoying Hua, Jianfei Chen, Chuyun Shao, Fan Tian, Hesham Y. A. Darwish, Yurong Tai, Xue Yang, Jianyu Chang, Yunfei Ma

**Affiliations:** ^1^ College of Veterinary Medicine, China Agricultural University, Beijing, China; ^2^ Research and Development Department for Breeding Poultry Feed, Shandong Hekangyuan Biological Breeding Co., Ltd, Jinan, China; ^3^ College of Animal Science and Technology, China Agricultural University, Beijing, China; ^4^ College of Veterinary Medicine, Northwest Agriculture and Forestry University, Yangling, China; ^5^ Department of Applied Biotechnology, Molecular Biology Researches & Studies Institute, Assiut University, Assiut, Egypt

**Keywords:** mucosal immunity, intraepithelial lymphocyte, reproduction, macrophage, cell migration

## Abstract

Endometrial immune response is highly associated with the homeostatic balance of the uterus and embryo development; however, the underlying molecular regulatory mechanisms are not fully elucidated. Herein, the porcine endometrium showed significant variation in mucosal immunity in proliferative and secretory phases by single-cell RNA sequencing. The loose arrangement and high motility of the uterine epithelium in the proliferative phase gave opportunities for epithelial cells and dendritic cells to cross talk with colonizing microbial community, guiding lymphocyte migration into the mucosal and glandular epithelium. The migrating lymphocytes were primarily NK and CD8^+^ T cells, which were robustly modulated by the chemokine signaling. In the secretory phase, the significantly strengthened mechanical mucosal barrier and increased immunoglobulin A alleviated the migration of lymphocytes into the epithelium when the neuro-modulation, mineral uptake, and amino acid metabolism were strongly upregulated. The noticeably increased intraepithelial lymphocytes were positively modulated by the bacteria in the uterine cavity. Our findings illustrated that significant mucosal immunity variation in the endometrium in the proliferative and secretory phases was closely related to intraepithelial lymphocyte migration, which could be modulated by the colonizing bacteria after cross talk with epithelial cells with higher expressions of chemokine.

## Introduction

As an important reproductive organ, the uterus contributes to providing an appropriate site for embryonic implantation and development. However, pregnancy failure and defective embryonic development are becoming more prevalent, often resulting from untoward changes in the uterine microenvironment (1, 2). During follicular and luteal phases of the ovarian cycle, the uterine mucosa is programmed to undergo various physiological changes in order to facilitate the implantation of the embryonic blastocyst (3, 4), while the mucosal epithelial cells (MEC) are transformed from pseudo-stratified columnar to simple columnar. In addition, the immune status of the uterine mucosa is closely related to successful embryo implantation, and an inhibited immune response is considered beneficial for implantation success (5). Regarding rodents and humans, researchers have found that natural killer cells assist in embryonic implantation into the endometrium, but non-inflammatory responses are induced during this process (2, 6, 7). Thus, although immune responses in the uterus vary at different times of the reproductive cycle, the underlying mechanisms are not yet fully understood.

Concerning mucosal immunity, several leukocyte populations comprise the important sentinel cells, including eosinophils, innate lymphocytes, and antigen-presenting cells. However, intraepithelial lymphocytes (IELs) constitute a large and highly conserved T-cell compartment and reduce the chances for pathogen invasion (8–10). The IELs in rodents and humans are identified as “natural”/thymic-derived IELs (nIELs) and as “induced”/peripheral IELs (pIELs) (11). The nIELs categorized as αβ and γδ T cells are present at birth and carry αβ and γδ T-cell receptors (TCR). pIELs arise from conventional TCRαβ^+^ cells after exposure to tissue-derived antigens (12). IELs exhibit a wide distribution and reside in the epithelium of all mucosal barrier sites, such as the skin, digestive tract, respiratory tract, and genital tract (13–16); Kobayashi et al. found that lymphocytes in the skin could inhibit sebaceous gland proliferation that produces antimicrobial peptides to reduce the colonization of commensal gram-positive bacteria (17). The IELs in the gut are also highly associated with pathogen resistance and mucosal barrier maintenance. It has been found the endometrial receptivity was closely related to the immune response and inflammation, and the lymphocytes and macrophages were the main immune cell populations in the human endometria across the menstrual cycle (18, 19). Due to various morphologies and motilities, the functions of different IEL populations and their underlying migratory mechanisms in different mucosal tissues remain to be clearly elucidated.

Commensal bacteria in mucosal tissues have been widely investigated and are reported to play significant roles in mucosal immunity by modulating the function of IEL (20–23). Hoytema van Konijnenburg et al. reported that the commensal luminal microbes recruited IEL into the epithelium by cross talk with epithelial cells, so as to maintain the intestinal epithelial barrier during infection (8). In the respiratory tract, the local microbiotas activate the lung-resident T cells and are involved in inflammation and tumor cell proliferation in lung cancer (24). These studies suggest that commensal bacteria play crucial roles in the homeostasis of the respiratory and gastrointestinal tracts. In view of particular physiological changes involved, we hypothesize that the composition of colonizing bacteria in the uterus might vary during different times of the estrous or menstrual cycle. In pigs, embryonic implantation depends upon the establishment of a relationship between the chorionic membrane and maternal endometrium—especially the epithelial cells—such that the IELs are very important for successful implantation. Thus, this study aims to investigate the various mucosal immune levels at proliferation and secretory phases and their molecular modulatory mechanisms involved.

## Results

### More Lymphocytes Migrated Into the Endometrium in the Proliferative Phase

**Figure 1:**
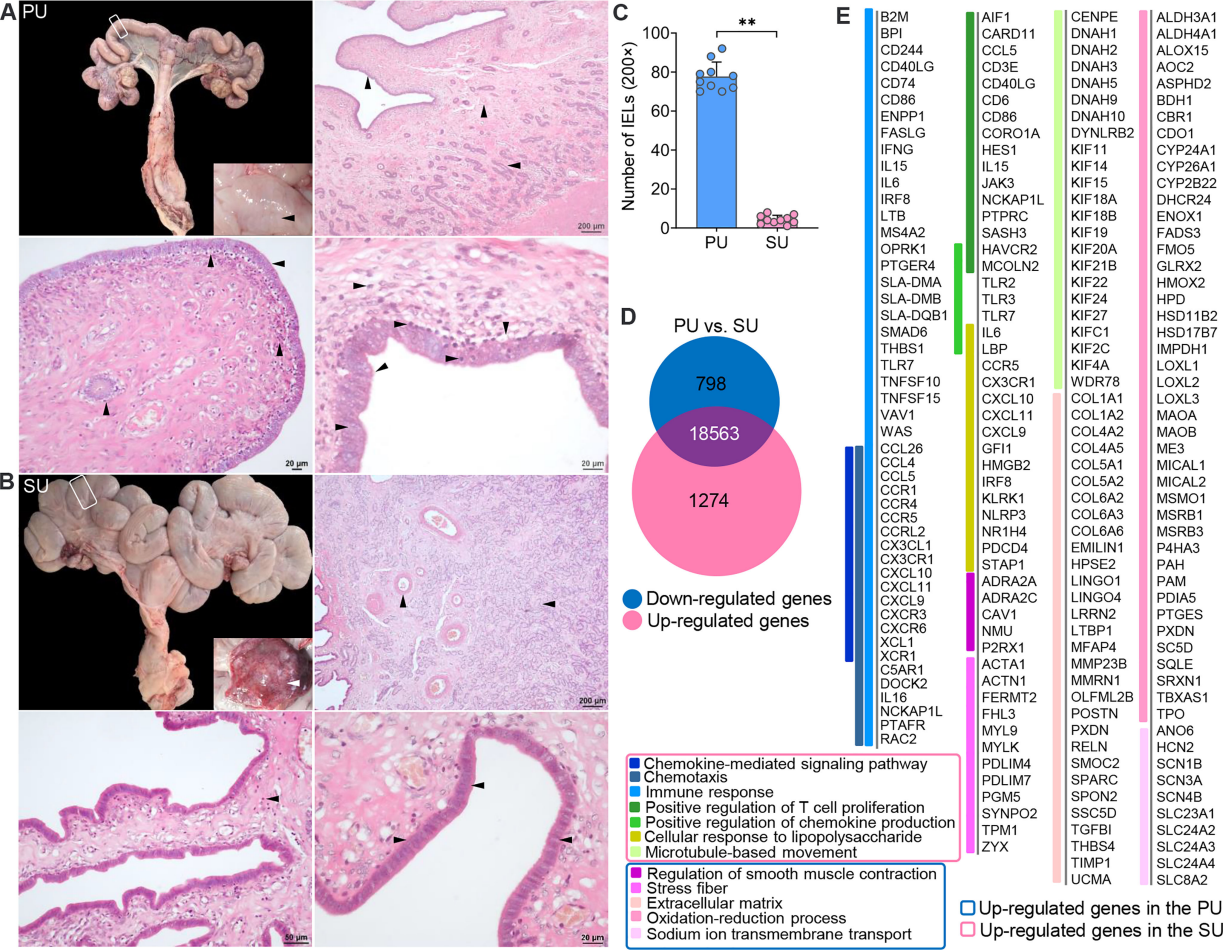
Obvious different histology and enriched pathways from differentially expressed genes (DEGs) of the endometrium at different phases. **(A)** Finger-like endometrium fold, pseudostratified columnar epithelium, and migrated lymphocytes in mucosal and glandular epithelium were obviously observed in the proliferative phase (PU). **(B)** Obvious vascular and uterine gland proliferation, endometrium fold reduction, and simple columnar epithelium were in the secretory phase (SU). **(C)** More intraepithelial lymphocytes (IELs) were significantly calculated in the mucosal epithelium in the PU under ×200 magnification. ^**^ *p* < 0.01. **(D)** A total of 1,274 upregulated genes and 798 downregulated genes were identified between the PU and SU. **(E)** The enriched pathways from the DEGs were closely related to chemotaxis, immune activation and cell migration in the PU, and obvious metabolism in the SU.

After dissection, obvious fingerlike plicae were observed in the uterus in the proliferative phase (PU; **Figure 1A**), and in the secretory phase (SU), we observed an enlarged uterine lumen, disappearance of the plicae, and angiogenesis (**Figure 1B**). In the PU, the mucosal and glandular epithelial cells were pseudostratified and columnar in configuration, which was loosely arranged with more intraepithelial lymphocyte migration in the mucosal and glandular epithelium (**Figures 1A, C** and **Figures S1A**–**C**). In the SU, in addition to significant arterial blood vessels and uterine gland proliferation, the epithelial cells transformed into simple columnar epithelium with increased basal membrane integrity and surfactant in the mucosal surface (**Figure 1B** and **Figures S1D**–**F**). The lymphocyte migration into the epithelium was only observed in the lamina propria of the endometrium (**Figure 1B** and **Figures S1D**–**F**). Moreover, we found more adipocytes located around the capillaries in the endometrium of SU (**Figure 1B** and **Figure S1F**). These results indicated that obvious differences of mucosal barrier were in the endometrium between PU and SU.

To investigate the possible pathways involved in the mechanism of different mucosal immunity especially IEL migration in the PU, we performed RNA sequencing (RNA-seq) to identify the differentially expressed genes (DEGs) in the endometrium. A total of 2,072 DEGs were identified, including 1,274 upregulated genes and 798 downregulated genes (**Figure 1D** and **Figure S2A**). By analyzing the enriched pathways in the PU, elevated expressions of genes were highly associated with chemotaxis, the chemokine-mediated signaling pathway, immune response, cellular response to lipopolysaccharide (LPS), positive regulation of chemokine production, and microtubule-based movement (**Figure 1E**, **Figure S2** and **Table S2**, **S3**), such as *B2M*, *BPI*, *CCL26*, *CCL4*, *CCL5*, *CCR1*, *CCR4*, *CCR5*, *FASLG*, *IFNG*, *IL15*, *IL6*, *IRF8*, *LTB*, *SMAD6*, *TLR7*, *TNFSF10*, *TNFSF15*, *XCL1*, and *XCR1* (**Figure S2B**), while the highly expressed genes in the SU were closely related to extracellular matrix (ECM), stress fibers, regulation of smooth muscle contraction, the oxidation–reduction process, and sodium ion trans-membrane transport (**Figure 1E**, **Figure S2C** and **Table S4**) including *FGF7*, *FGFR4*, *IGF1*, *TGFBI*, *TGFB1L1*, and *TFRC* (**Figure S2B** and **Table S2**). After analyzing the Kyoto Encyclopedia of Genes and Genomes (KEGG) pathways of the DEGs, we demonstrated that the top pathways in the PU were primarily related to infection, graft *versus* host disease, allograft rejection, chemokine signaling, immune cell differentiation, IgA production, natural killer cell-mediated cytotoxicity, and leukocyte trans-endothelial migration (**Figure S2D** and **Table S5**). In the SU, the top pathways were focal adhesion, ECM–receptor interaction, protein digestion and absorption, dilated cardiomyopathy, vascular smooth muscle contraction, ovarian steroidogenesis, metabolism, long-term depression, and mineral absorption (**Figure S2D** and **Table S6**). All the results indicated that the immune statuses between the PU and SU were very different when more lymphocytes migrated into the epithelium of the PU; and their migration was closely related to enhanced immune response and chemokine gene expressions in the endometrium.

### Mucosal Immunity Difference Highly Associated With Cell Population Variation

**Figure 2:**
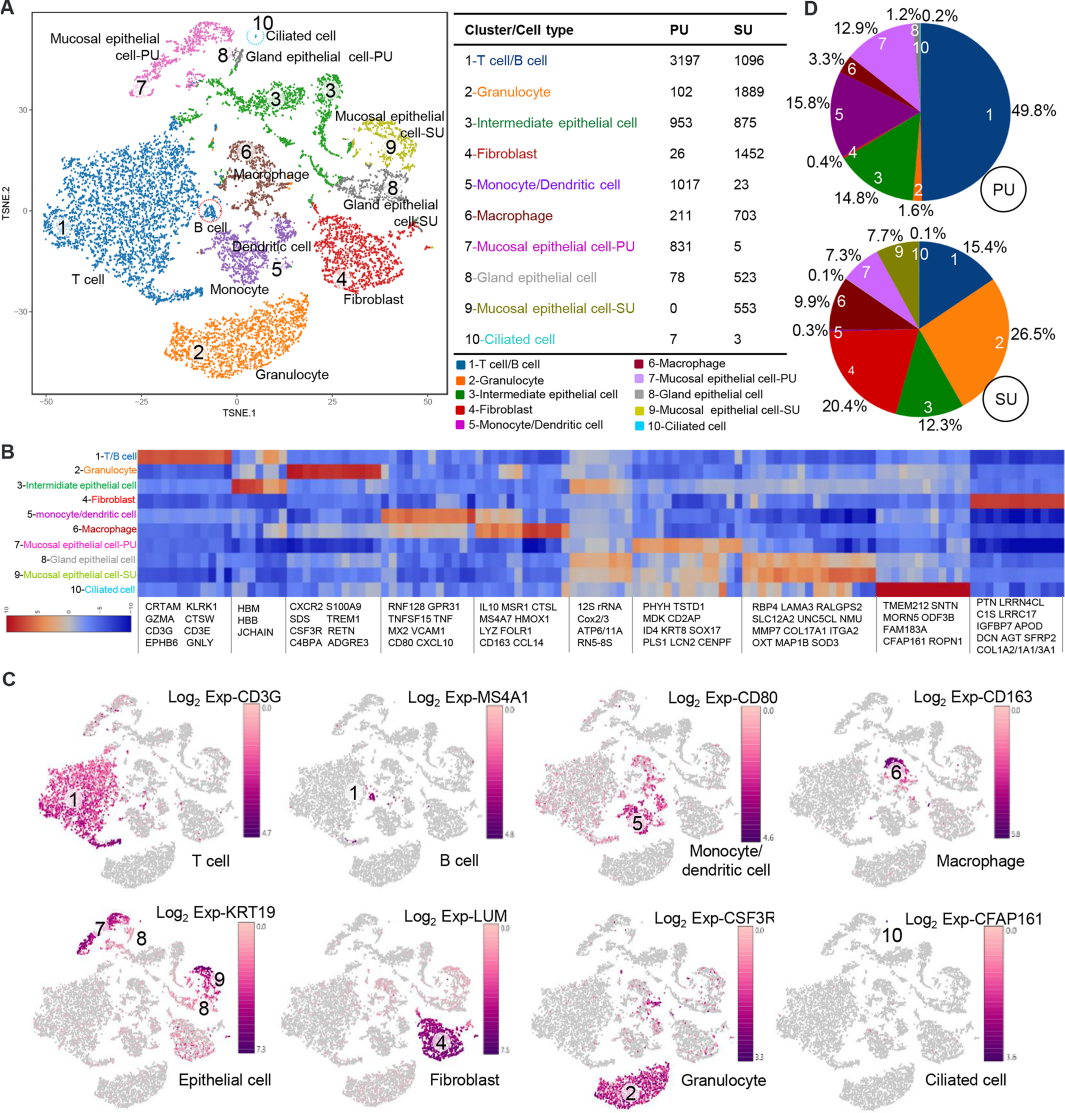
Different cell populations in the endometrium of proliferative phase (PU) and secretory phase (SU). **(A)** Cell populations of the endometrium were counted and could be divided into 10 clusters, and the mucosal and glandular epithelial cells of PU and SU were significantly separated. **(B)** Specific gene expressions in the 10 cell populations identified from single-cell RNA-seq (scRNA-seq). **(C)** The different cell populations were identified according to their marker gene expressions. **(D)** More numbers of lymphocytes, monocytes, and dendritic cells were identified in the PU; and more granulocytes, fibroblasts, and macrophages were in the SU.

On account of the histological variation and DEGs in the endometrium of PU and SU, we conducted single-cell RNA-seq (scRNA-seq) to analyze the differences in cell populations and gene expressions in specific cells. The endometrium in the proliferative and secretory phases was dissociated into single cells, which were captured using the droplet-based microfluidic system chromium (10× Genomics) (**Figure S3A**). We obtained 13,544 good-quality cells, 6,422 in the PU and 7,122 in the SU, and conducted aggregating multiple GEM groups (AGGR) to reanalyze the cell clusters and expressed genes (**Figures S3B, C**). The cells from the PU and SU were divided into 10 clusters visualized after tSNE projection according to the correlation of identified cell populations (**Figure 2A**). According to gene expressions (**Figure 2B**), the cell types were characterized by the expression of marker genes, and 10 cellular populations were characterized by the expressions of *CD3G*, *MS4A1*, *CSF3R*, *CD80*, *CD163*, *KRT19*, *LUM*, and *CFAP161* (**Figure 2C**). We found the cell populations between the PU and SU varied significantly with greater gene expressions in the epithelial cells, monocytes, and lymphocytes in the PU; and epithelial cells, macrophages, and fibroblasts in the SU (**Figure S3C**). In the PU, the T lymphocytes (49.8%), monocytes, and dendritic cells (15.8%) were the major cellular populations, whereas granulocytes (26.5%), macrophages (9.9%), and fibroblasts (20.6%) constituted the primary cell clusters in the SU (**Figure 2D**). It is interesting to note that a few MEC were clustered as ciliated cells, especially in the PU, which highly expressed specific genes including *ROPN1*, *ROPN1L*, *CFAP45*, *CFAP161*, *ODF3B*, *DTHD1*, and *MORN5* (**Figure 2B**).

**Figure 3:**
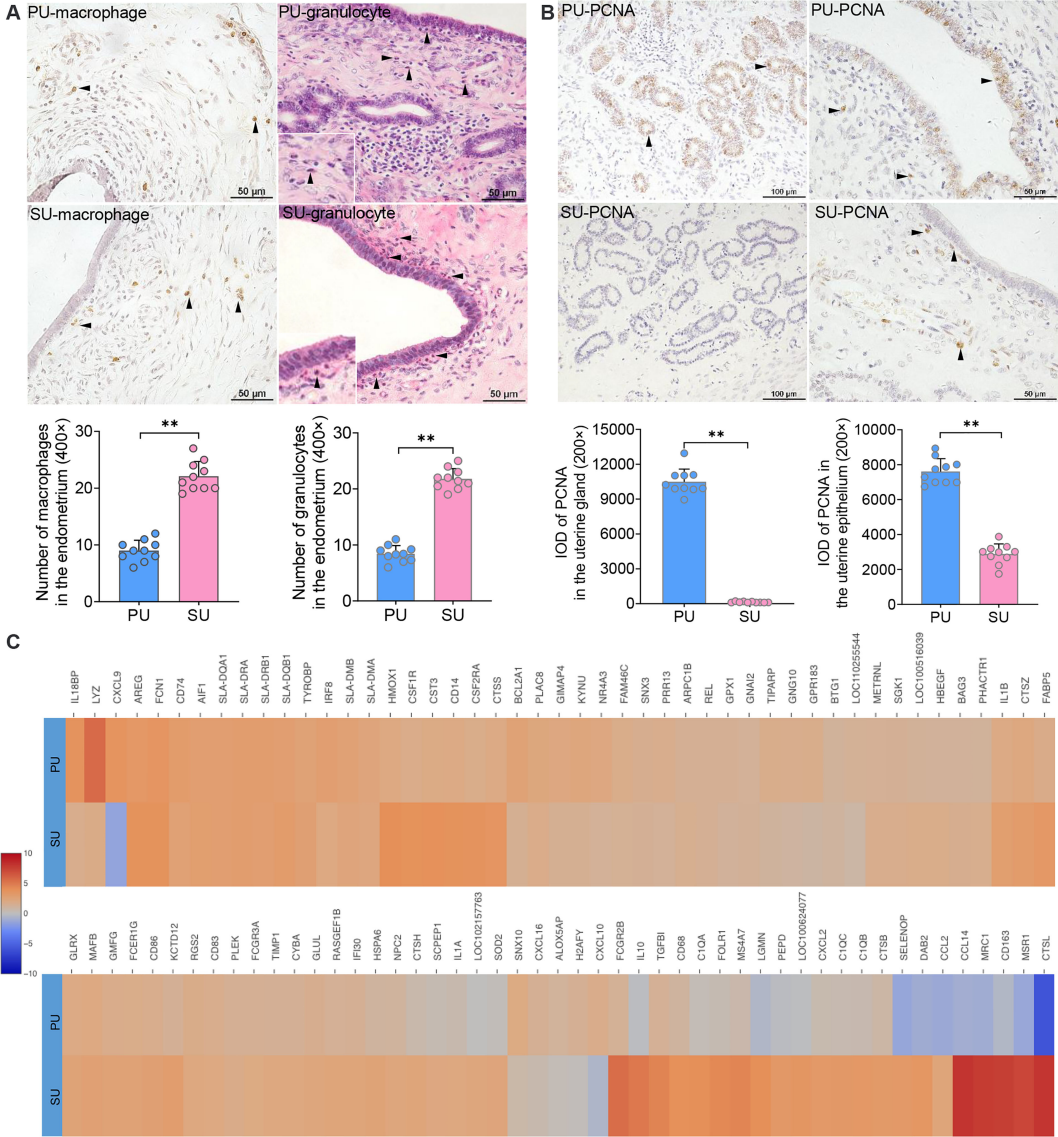
Major cell population identification and gene expression in epithelial cell and macrophages. **(A)** Cell populations of macrophages (brown) and granulocytes (red granules in cytoplasm) in the endometrium of proliferative phase (PU) and secretory phase (SU), and more numbers were identified in the SU. ^**^ *p* < 0.01. **(B)** Obvious proliferating cell nuclear antigen (PCNA) expressions (brown) were observed in the mucosal and glandular epithelial cells of PU when those were only observed in the vascular endothelial cells and stromal cells in the SU. More PCNA expressions were calculated in the PU. ^**^ *p* < 0.01. **(C)** Significant differences of gene expressions in the macrophages were identified between the PU and SU when *CXCL9* and *CXCL10* were highly detected in the PU and *IL10*, *FCGR2B*, *CCL2*, *CCL14*, *CXCL2*, *CD163*, *CTSL*, and *MSR1* in the SU.

According to the identified cell populations, it is strongly consistent that more IELs were observed in the mucosal and uterine glandular epithelium of the PU (**Figure 1A**). Moreover, in the SU, more granulocytes were observed in the submucosal epithelium, and more macrophages were distributed in the lamina propria of the endometrium, which was in line with the scRNA-seq results (**Figure 3A**). In view of the significant differences in histology of the MEC between PU and SU, we found the proliferating cell nuclear antigen (PCNA) expressions were only observed in the mucosal and uterine glandular epithelial cells in the PU, whereas in vascular endothelial cells and fibroblasts in the SU (**Figure 3B**). By analyzing the expressed genes associated with cell recruitment, we found in macrophages that chemokines *CXCL9*, *CXCL10*, and *CXCL16* were highly identified in PU when higher *HMOX1*, *SOD2*, *MRC1*, *MSR1*, *CTSL*, *CD163*, *IL1A*, *IL1B*, *IL10*, *CXCL2*, *CCL2*, and *CCL14*, were in SU (**Figure 3C**).

### Intraepithelial Lymphocyte Migration Obviously Modulated by Epithelial Cells

**Figure 4:**
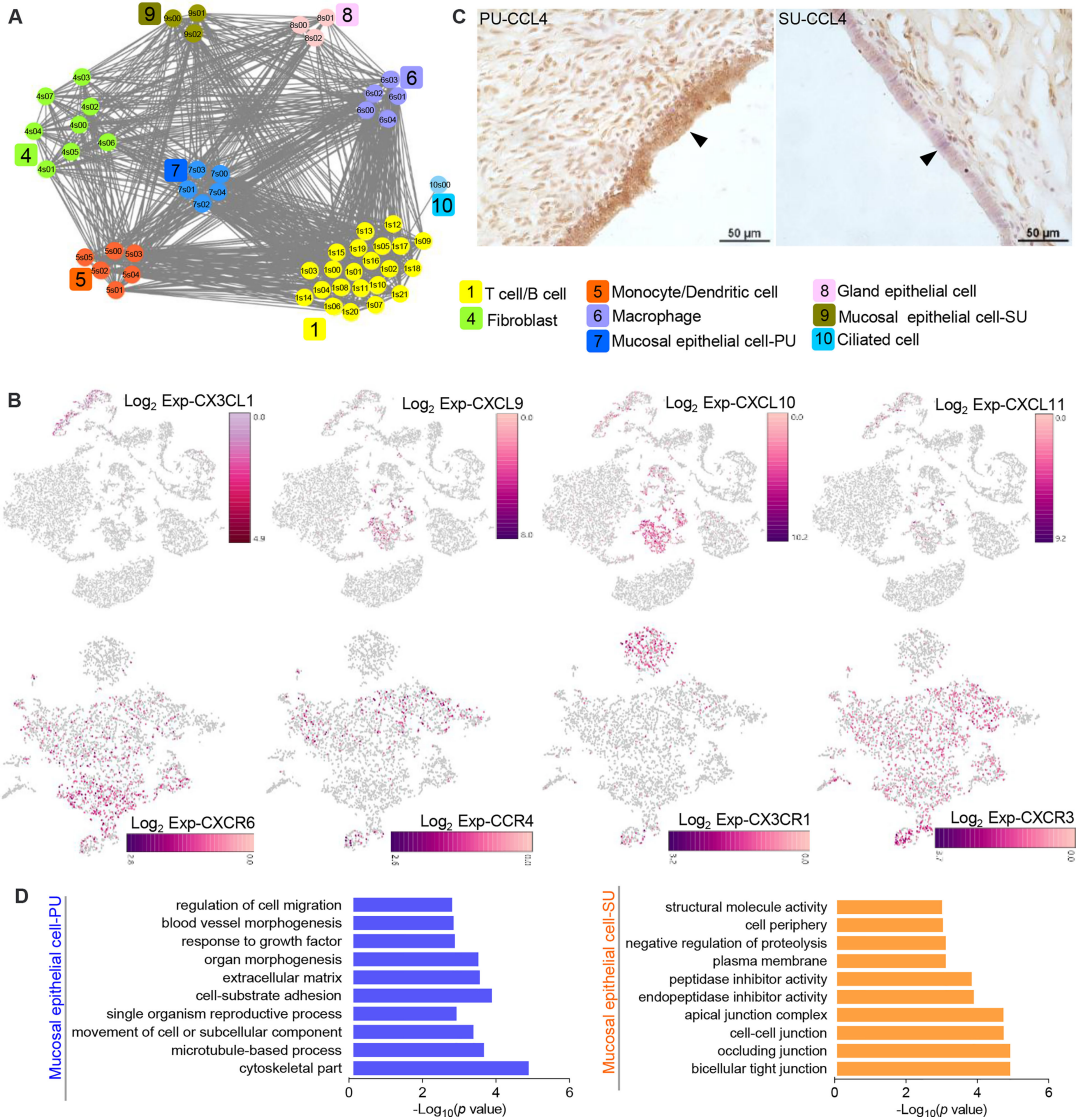
The epithelial cells in the proliferative phase (PU) and secretory phase (SU) show differential morphologies. **(A)** Interaction network between different cell populations were analyzed, and significant interactions between the epithelial cells and macrophages, monocytes, and dendritic cells were identified in the PU. **(B)** Chemokine genes and related receptor gene expressions were obviously detected in the epithelial cells, monocytes, dendritic cells, and lymphocytes. **(C)** Higher expressions of CCL4 in the mucosal epithelial cells were detected in the PU by immunohistochemistry. **(D)** The enriched Gene Ontology (GO) terms for highly expressed genes in the epithelial cells of PU and SU were significantly different.

On account of the significant variation in IELs, we further analyzed the cell–interaction networks to elucidate what types of cells were involved in the modulation of IEL migration in the PU. We found the epithelial cells, macrophages, monocytes, and dendritic cells were closely interacted with T and B lymphocytes (**Figure 4A**), while chemokine genes *CXCL9*, *CXCL10*, *CXCL11*, *CX3CL1*, and *IL15* were mainly detected in the MEC, monocytes, and dendritic cells in the PU (**Figure 4B** and **Figure S4A**). Moreover, *CCR4*, *CXCR3*, *CXCR6*, *CX3CR1*, *IL2RB*, and *IL2RG* were highly detected in the lymphocytes (**Figure 4B** and **Figure S4A**). By immunohistochemistry analysis, we detected a higher expression of CCL4 in the MEC in the PU (**Figure 4C**). Gene expressions in the MEC revealed that regulation of cell migration, cell-substrate adhesion, movement of cell or subcellular component, and microtubule-based process were highly enriched in the PU (**Figure 4D** and **Table S7**), whereas bicellular tight junction, occluding junction, cell–cell junction, apical junction, endopeptidase and peptidase inhibitor activities, and negative regulation of proteolysis were highly enriched in the SU (**Figure 4D**), demonstrating that the MEC had played different roles during proliferative and secretory phases.

**Figure 5:**
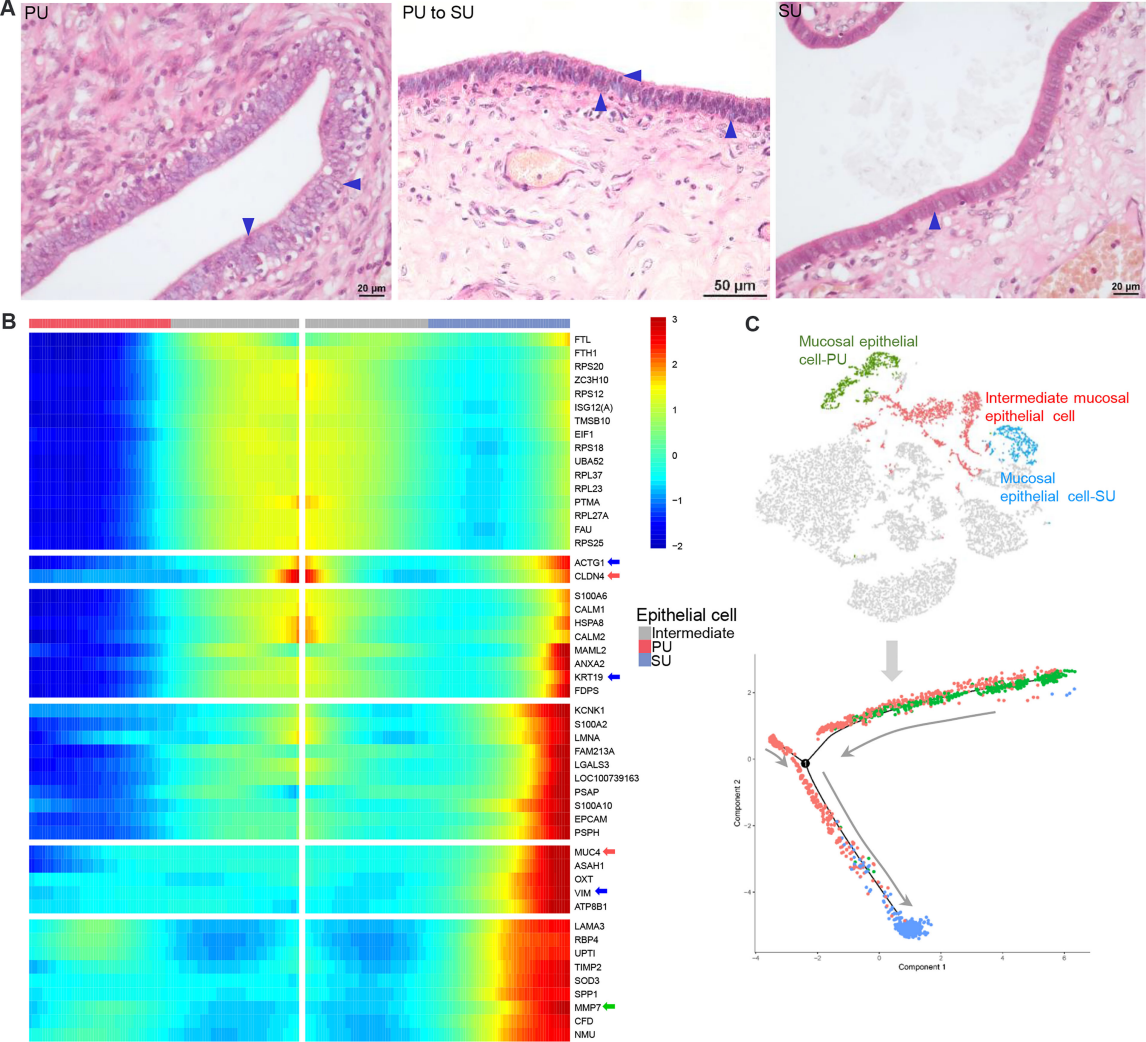
The differences of mucosal epithelial cells (MEC) with different gene expressions in the proliferative phase (PU) and secretory phase (SU). **(A)** Morphological changes of MEC were obviously identified from pseudostratified columnar epithelium in the PU to simple columnar epithelium in the SU. **(B)** During morphological changes of the MEC, they could be significantly divided into distinct two populations with specific gene expressions when one population of intermediate mucosal epithelial cell was identified. **(C)** The Monocle trajectory analysis showed that the identified mucosal epithelial cell populations changed from PU to SU with one intermediate cell population.

By observing the MEC morphology, we found that in the PU they appeared foamy with a loose arrangement, programed into intermediate cells that are shown as two significant morphologies with significant surfactant in the mucosal surface and then transformed into a tight single-cell layer with a small amount of cytoplasm (**Figure 5A**). Due to the characteristic physiological changes occurring from the PU to SU, the mucosal and glandular epithelial cells in PU and SU were divided into different clusters according to their gene expression (**Figure 5B**). Using Monocle trajectory analysis, we noticed that the intermediate epithelial cell population transited from the proliferative phase to the secretory phase, with higher expressions of ribosomal protein genes (**Figures 5B, C**). In the SU, the epithelial cells showed different physiological functions, with a higher expression of related genes, such as *MUC4*, *KRT19*, *VIM*, *CLAN4*, *S100A2*, *S100A10*, *MMP7*, and *SOD3* (**Figure 5B**), which were very related to mucus, structural integrity of epithelial cells, cell cycle progression and differentiation, and anti-oxidation. Commensurate with the great variation in the number of migrating lymphocytes, we found that gene expression also varied between lymphocytes in the PU and SU. The lymphocytes in the PU were identified as primarily CD8^+^ T cells and natural killer cells, which showed a high expression of *CCL5*, *LTB*, and *GPR183* (**Figure S4B**). According to the results of RNA-seq, we found that the G-protein-coupled receptor genes, including *GPR171*, *GPR183*, *GPR31*, *GPR34*, *GPR65*, and *GPR85*, showed a significantly higher expression in the PU (**Table S2**), which were highly detectable in the lymphocytes, granulocytes, and macrophages by the scRNA-seq (**Figure S6**). These results presented above provided clues that the MEC in the proliferative and secretory phases manifested very different physiological functions, which were closely related to modulate lymphocyte migration.

### Colonizing Bacteria Guide Intraepithelial Lymphocyte Recruitment Through Chemokine Signaling Pathway

**Figure 6:**
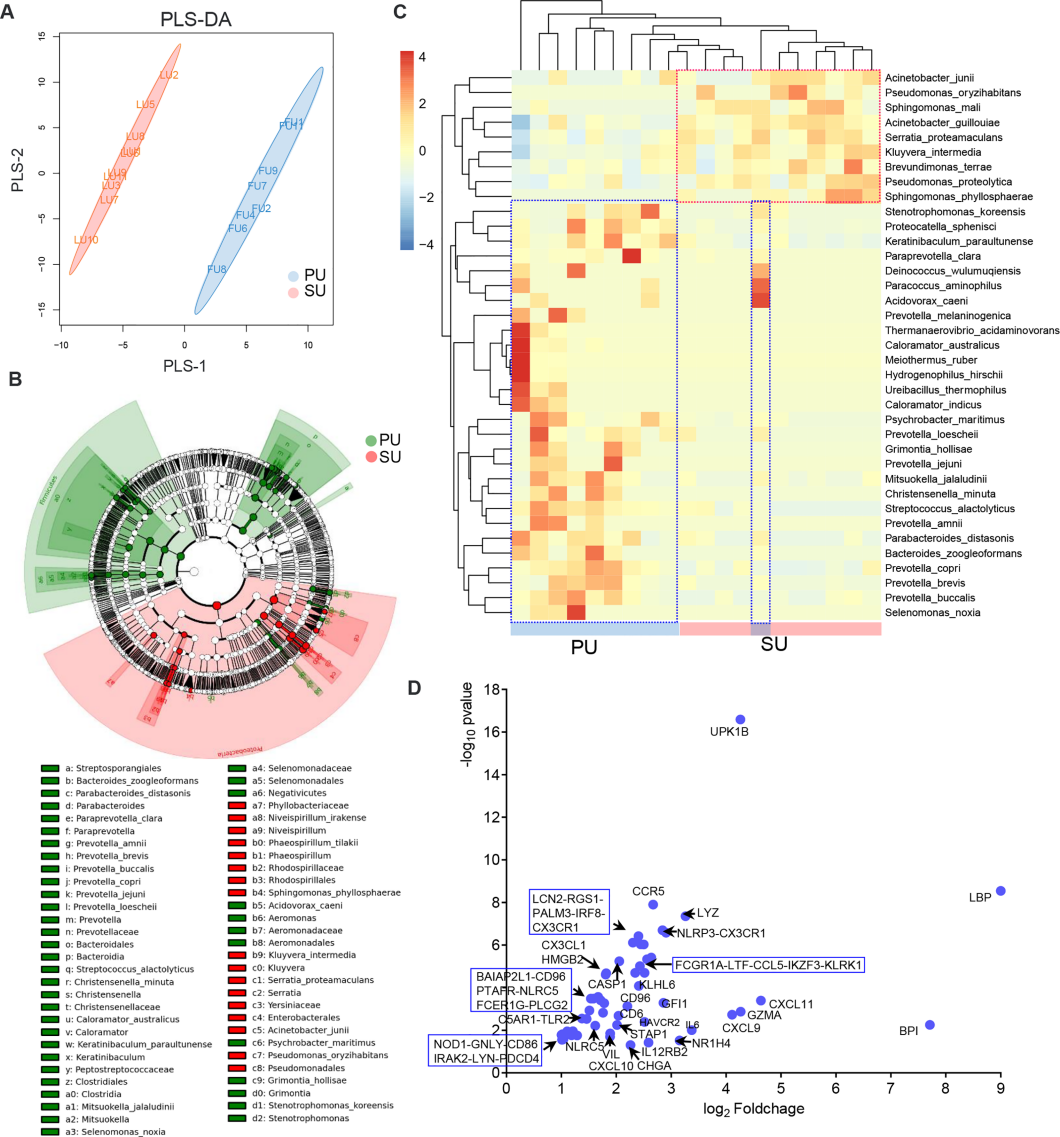
The differences of colonizing bacteria and upregulated immune genes in the proliferative phase (PU) and secretory phase (SU). **(A)** The colonizing bacteria in the PU and SU were obviously grouped. **(B)** The compositions of colonizing bacteria were significantly different between the PU and SU. **(C)** The top clusters of bacterial species in the PU and SU were significantly different and could be divided into specific clusters. **(D)** Upregulated genes associated with immune responses to bacteria were only highly detected in the PU.

Based on the important roles of commensal bacteria in mucosal immunity by modulating IEL function, we postulated that the presence of colonizing bacteria in the uterine lumen might be involved in the recruitment of lymphocytes to the epithelium in the PU. To elucidate it, we firstly collected the luminal bacteria and conducted 16S rRNA full-length sequencing (**Figure 6A**). anonis/permanova analysis indicated that the compositions of colonizing bacteria were significantly different between the PU and SU (*p* = 0.006, **Table S8**). The result of linear discriminant analysis (LDA) effect size (LEfSe) analysis indicated that the different species in the PU belonged to Firmicutes and Bacteroidia. By contrast, in the SU, they were species of Proteobacteria (**Figure 6B** and **Figure S6A**). The relative abundance of Firmicutes and Bacteroidia, such as *Ureibacillus_thermophilus*, *Streptococcus alactolyticus*, *Christensenella minuta*, *Caloramator australicus*, *Proteocatella sphenisci*, *Bacteroides zoogleoformans*, *Parabacteroides distasonis*, *Paraprevotella clara*, and *Prevotella copri*, in the PU was higher than in the SU (*p* = 0.011, **Table S8**), whereas in the SU, Proteobacteria, including *Brevundimonas terrae*, *Kluyvera intermedia*, *Serratia proteamaculans*, *Acinetobacter guillouiae*, and *Pseudomonas proteolytica*, were higher than in the PU (*p* = 0.007, **Table S8**). We chose the top 50 species to analyze the cluster variation, and we discovered that the species were divided into two branches, which suggested that there were obvious differences in the dominant species between the PU and SU (**Figure 6C**). By analyzing the functions of the commensal bacteria, we observed that cell motility and environmental adaptation were highly enriched in the PU (**Figure S6B**). Furthermore, RNA-seq analysis indicated that the genes associated with responses to bacteria and cellular responses to LPS were only highly detected in the PU, including *LBP*, *BPI*, *NOD1*, *TLR2*, *TLR3*, *LTF*, *LYZ*, *NLRP3*, *NLRC5*, *CASP1*, *LCN2*, *FCER1G*, *FCGR1A*, and *CHGA* (**Figure 6D**).

**Figure 7:**
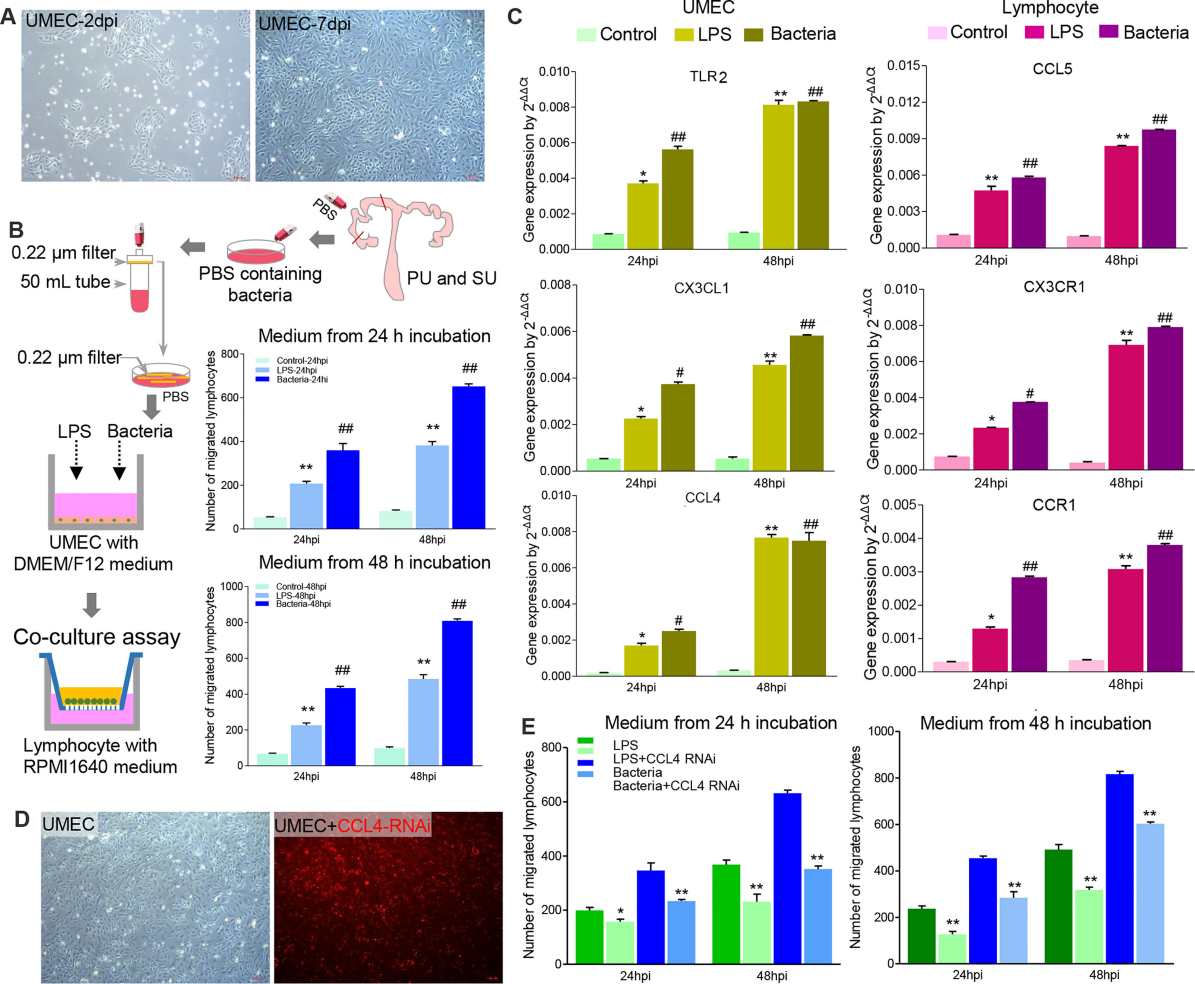
The colonizing bacteria guided lymphocyte migration by upregulating chemokine gene expressions in the mucosal epithelial cells. **(A)** Primary uterine mucosal epithelial cells (UMEC) were isolated and cultured *in vitro*. **(B)** The colonizing bacteria were collected from the proliferative phase (PU) and secretory phase (SU) and exposed to the UMEC. Like lipopolysaccharide (LPS), the supernatant from the colonizing bacteria-treated UMEC significantly recruited lymphocyte migration. ^**^ *p* < 0.01; ^##^ *p* < 0.01. **(C)** LPS and colonizing bacteria induced higher expressions of *TLR3*, *CXCL3*, and *CCL4* in the UMEC, and migrated lymphocytes increased expressions of *CCL5*, *CXC3R*, and *CCR1*. ^*^ *p* < 0.05; ^**^ *p* < 0.01; ^#^ *p* < 0.05; ^##^ *p* < 0.01. **(D)** The UMEC were efficiently transfected with *CCL4* RNAi (red fluorescence). **(E)** Downregulation of *CCL4* expression in the UMEC significantly reduced the lymphocyte migration after being treated with LPS and colonizing bacteria. ^*^ *p* < 0.05; ^**^ *p* < 0.01.

To investigate how the resident bacteria upregulated immune gene expression and modulated IEL migration in the PU, we isolated primary uterine MEC (UMEC; **Figure 7A**) and splenic lymphocytes. When the UMEC were exposed to LPS or collected colonizing bacteria, their culture supernatant could induce significant lymphocyte migration (**Figure 7B**). It should be noted that gene expressions for *TLR2*, *CXC3L*, and *CCL4* significantly increased in the UMEC, and the recruited lymphocytes highly expressed *CCL5*, *CXCR3*, and *CCR1* (**Figure 7C**). Thus, these results indicated that colonizing bacteria in the uterine lumen could communicate with the MEC and be involved in modulating lymphocyte migration in the endometrium. Moreover, we observed knock-down chemokine *CCL4* expression in the UMEC (**Figure 7D**); after exposure to collected colonizing bacteria or LPS, their culture supernatant significantly inhibited lymphocyte migration (**Figure 7E**). Herein, we demonstrated that the MEC in the PU strongly modulated lymphocyte migration into the epithelium through a chemokine signaling pathway, which is closely related to the colonizing bacteria.

### Strengthened Mucosal Barrier Inhibits Lymphocyte Migration in the Secretory Phase

**Figure 8:**
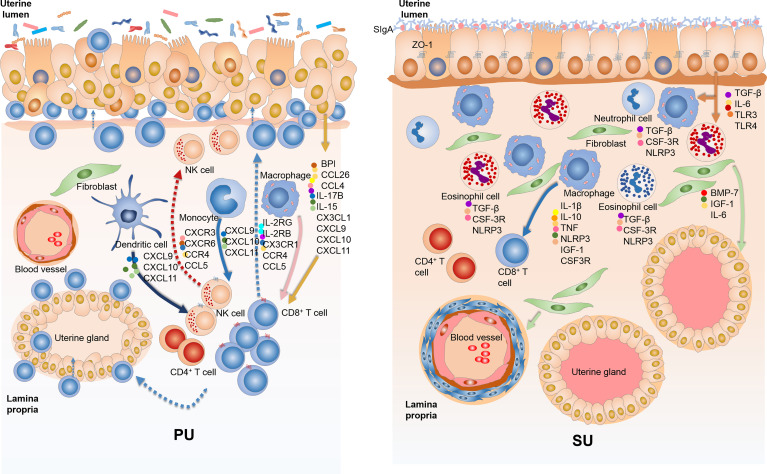
The variation of mucosal immunity in the endometrium of proliferative phase (PU) and secretory phase (SU). **(A)** The loosely arranged epithelial cells give the opportunity for the colonizing bacteria to cross talk with epithelial cells, macrophages, and dendritic cells and then guide intraepithelial lymphocyte (IEL) migration into the epithelium by upregulation of chemokine expressions. **(B)** The endometrium epithelium of SU programmed tightly arranged epithelial cells and integrated basal membrane, and more sIgA and tight junction proteins prohibited luminal bacteria colonization, reducing lymphocyte migration.

Different from more IELs and an enhanced immune response in the mucosal immunity of the PU, what is the underlying mechanism of mucosal immunity in the SU, and what prevents lymphocyte migration? The DEGs identified from RNA-seq showed that upregulated genes in the SU were highly associated with focal adhesion, ECM, actin binding, collagen trimer, actin cytoskeleton, stress fibers, and regulation of smooth muscle contraction (**Table S4**, **S6**). Compared with those in the PU, a higher expression of *NLRP3*, *IL1β*, *TNF*, and *IL10* in macrophages; *CSF3R* and *TGFβ* in granulocytes; and *IL17B*, *IL6*, *BMP7*, and *IGF1* in fibroblasts were detected (**Figure S7**). According to the highly expressed genes in the MEC of the SU were considerably enriched for the mechanical barrier of bicellular tight junction, occludin junction, cell–cell junction, apical junction complex, endopeptidase inhibitor activity, peptidase inhibitor activity, and negative regulation of proteolysis, we discovered that a higher expression of toll-like receptor 4 (TLR4), secretory immunoglobulin A (sIgA), tight junction proteins ZO-1, claudin, and occludin was present in the mucosa of the SU (**Figure S8**). These results indicated the mucosal immunity in the PU and SU was markedly different and could be positively modulated by the colonizing bacteria (**Figure 8**).

## Discussion

As for intestinal mucosal immunity, IELs serve a pivotal immunologic surveillance function for the mucosal barrier (25–28). In the present study, we found in swine endometrium during the proliferative phase that more IELs are distributed in the mucosal and glandular epithelium. Also, the epithelium of the PU with increased permeability provided opportunities for lymphocyte migration, which were recruited by epithelial cells after cross talk with constitutively colonizing bacteria. A previous study showed that lymphocyte recruitment in the skin was regulated by *CCR6* and that this lymphocyte inhibited sebaceous gland proliferation, which can produce antimicrobial peptides to reduce the colonization of commensal gram-positive bacteria (17). In this study, we noticed that lymphocyte migration in the PU was closely related to a higher expression of chemokine genes that were principally from the MEC, macrophages, and dendritic cells when the colonizing bacteria are engaged in this process. Additionally, it has been reported that interleukin 15 (*IL15*) plays important immunologic surveillance roles in modulating the residential lymphocyte response that entails a higher expression of NK1.1, CD49a, and CD103 (11, 29, 30). In our work, we revealed that *IL15* was highly expressed in the MEC of PU and that the lymphocytes showed a commensurately high expression of *IL2RB* and *IL2RG*, indicating that the MEC might modulate lymphocyte proliferation and immune responses by the IL15 signaling pathway. Many studies reported that bacteria and their metabolic products interacted with epithelial cell-modulating lymphocyte migration and function in order to maintain homeostasis (30–34), which could then drive and activate T cells to promote tumor cell proliferation (35). Herein, we found that in the PU, the types of colonizing bacteria were significantly different from those in the SU and that the genes associated with immune response, natural killer cell-mediated cytotoxicity, T-cell differentiation, and inflammatory response were only detected in the PU. The luminal colonizing bacteria from the PU upregulated chemokine gene expression in epithelial cells to recruit lymphocytes that allowed their migration. In addition, higher proliferation capabilities were only detected in epithelial cells of the PU. Therefore, we concluded that the migrated lymphocytes might be involved in mucosal barrier formation and be closely related to proliferation of the mucosal epithelium and uterine gland in the proliferative phase.

In contrast, the secretory phase reflects vigorous secretion of the uterine glands. We observed an abundance of secreted sIgA on the mucosal surface, which might inhibit the colonization of bacteria and reduce its communication with the MEC. Moreover, the tight arrangement of the MEC with higher expressions of tight junction proteins further strengthened mechanical mucosal barrier to prevent the recognition of commensal bacteria by epithelial cells, macrophages, and dendritic cells, which reduced the immune response in the endometrium and hindered lymphocyte migration. Because the SU provides a specific place for embryonic implantation, the reduced resident bacteria, presence of fewer IEL, and inhibited immune responses allow for the appropriate physiological changes to take place in epithelial cells, thus providing an adequate site for implantation and subsequent development of the embryo. According to previous studies, endometrial decidualization was shown to be important for embryo implantation when the MEC played key roles (2, 36, 37). Although the placenta of the pig does not program a decidual response before implantation, we also found genes corresponded with endometrium-programmed decidualization highly expressed in the SU. These genes were involved in macrophages, granulocytes, and fibroblasts, including *IL1β*, *IL6*, *IL10*, *TNF*, *CSF3R*, *TGFβ*, *BMP7*, and *IGF1*. The principal cell populations in the endometrium of SU were the MEC, fibroblasts, granulocytes, and macrophages, so we inferred that they played important roles in embryo implantation in pregnant pigs. An in-depth investigation of their roles and related molecular mechanism would further help us to better understand the endometrial decidualization. Additionally, we noted that macrophage was the principal cell population in the SU with higher expressions of folate receptor (*FOLR*), *HMOX1*, *TFRC*, *GPX1*, and *SOD2*, which might be closely related to nutritional immunity, heme metabolism, and ferric iron absorption and utilization. Moreover, we found *IL10* highly expressed in macrophages and other anti-inflammatory cytokines that increased in the endometrium of the SU, which would build weak immune microenvironment to help embryo implantation.

As for some specific reproductive diseases, we speculated that their pathogenesis would be noticeably associated with the different cellular populations in the endometrium, which may provide different opportunities for the invasion of specific pathogens. We explored that the PU had a greater number of lymphocytes with enhanced CCR5 expression, which would aid in their migration and increase the chance of infection with specific pathogen, such as the human immunodeficiency virus (38, 39). It is known that *CD163* is a key gene for pigs infected with the porcine reproductive and respiratory syndrome virus. Hence, we found more macrophages in the SU with increased *CD163* expression, which could be linked to reproductive failure in pregnant sows (40). Additionally, it has been reported that the increased permeability of the intestinal barrier might lead to secretory IgA leakage and IgA–C3 complex deposition in cardiovascular lesions (41). We can thus deduce that if the strengthened mechanical mucosal barrier was destroyed during the secretory phase, accompanied by increased permeability, this would result in greater sIgA leakage and provide an opportunity for bacteria invasion, thereby inducing a severe inflammatory response that would result in embryo implantation failure, abortion, or stillbirth in pregnant sows.

In conclusion, our work illustrated that the architecture and function of the immune barrier of the uterine mucosa were very different between proliferative and secretory phases. The mechanical mucosal barrier in the secretory phase was less permeable relative to the increased permeability of the mucosal epithelium during the proliferative phase, which allowed for opportunities of cross talk between the colonizing bacteria and epithelial cells. We demonstrated that their cross talk could upregulate chemokine CCL4 production in the MEC by TLR signaling pathway, thus recruiting lymphocytes to migrate into the epithelium.

## Materials and Methods

### Animals and Sample Collection

The uteri were collected from healthy 6-month-old Large White pigs at the animal farm of China Agricultural University, Beijing. Before sampling, the pigs’ sera were isolated and evaluated negative for porcine reproductive and respiratory syndrome virus, porcine pseudorabies virus, swine fever virus, porcine parvovirus, and porcine circovirus. The uteri in the proliferative phase (PU) were identified as all ovarian follicles with no corpus luteum, and the uteri in the secretory phase (SU) were identified with obvious three to four corpora lutea. Twelve pigs in the proliferative phase and 12 pigs in the secretory phase were chosen and anesthetized by carbon dioxide. After ligation of portio vaginalis, the uteri were isolated and sampled. As shown in **Figure 1**, on one side of the uterine horn, nearly 5 cm was cut into five pieces: one was fixed in 4% paraformaldehyde for histological analysis, two were stored in liquid nitrogen for quantitative PCR and transcriptome analysis, and one was dissociated for scRNA-seq. The other side of the uterine horn was washed with sterile phosphate-buffered saline (PBS) and then prepared for 16S rRNA sequencing and colonizing bacteria collection. Three specific pathogen free (SPF) Large White pigs aged at 4 weeks were obtained from the Beijing Centre for SPF Swine Breeding & Management to isolate primary cells. The protocols for animal use and experimentation were approved by the Beijing Association for Science and Technology (approval ID, SYXK [Beijing] 2007–0023) and were in compliance with the Beijing Laboratory Animal Welfare and Ethics guidelines. All animal research work was also approved by the Beijing Administration Committee of Laboratory Animals and was in accordance with the China Agricultural University (CAU) Institutional Animal Care and Use Committee guidelines (ID: SKLAB-B-2010-003).

### Histology

After fixation in 4% paraformaldehyde for at least 24 h, the uteri (n = 12 for each phase) were then trimmed of fat and connective tissue, dehydrated using a graded series of alcohols, embedded in paraffin, and cut into 5-µm sections. For histological observation, the sections were dewaxed, rehydrated, and stained with hematoxylin (Zhongshan Golden Bridge Biotechnology Co. Ltd, Beijing, China) for 5 min. After being rinsed with distilled water, the sections were treated with 1% HCl in 75% alcohol for 20 s and then incubated in PBS for 10 min. The sections were incubated in 70% and then 80% alcohol for 2 min each and then stained with eosin (Zhongshan Golden Bridge Biotechnology Co. Ltd, Beijing, China) for 30 s. After destaining in 95% alcohol for 1 min, the sections were dehydrated in 100% alcohol followed by xylene and were mounted for light microscopic observations. The numbers of IELs and granulocytes in the PU and SU were counted under microscope at ×200 and ×400 magnification, respectively, by one investigator.

### RNA Sequencing

Based on the histological observation, the uteri (n = 3 for each phase) were chosen for transcriptome analysis. Total RNA was isolated using the TRIZOL^®^ reagent (Invitrogen, Shanghai, China) according to the manufacturer’s instructions. RNA degradation and contamination on 1% agarose gels were visualized; RNA purity was checked using a NanoPhotometer^®^ spectrophotometer (IMPLEN, CA, USA); and concentrations were determined using the Qubit^®^ RNA Assay Kit in Qubit^®^ 2.0 Fluorometer (Life Technologies, CA, USA). RNA integrity was assessed using the RNA Nano 6000 Assay Kit of the Bioanalyzer 2100 system (Agilent Technologies, Shanghai, China), and a total of 3 µg of RNA from each sample was used as the input material for RNA sample preparations. The ribosomal RNA was removed using an Epicentre Ribo-zero™ rRNA Removal Kit (Epicentre), and the mRNA sequencing libraries were constructed using an NEBNext^®^ Ultra™ Directional RNA Library Prep Kit for Illumina^®^ (NEB), according to the manufacturer’s recommendations. The mRNA libraries were then sequenced on an Illumina HiSeq 2000 platform, and 100-bp paired-end reads were generated.

The transcriptome sequencing and analysis were conducted by OE biotech Co., Ltd. (Shanghai, China). Raw data (raw reads) were processed using Trimmomatic. The reads containing poly-N and the low-quality reads were removed to obtain the clean reads. The clean reads were mapped to *Sus scrofa* (pig) genome (ftp://ftp.ensembl.org/pub/release-90/fasta/sus_scrofa/dna/) using hisat2. The Fragments Per Kilobase per Million mapped reads (FPKM) value of each gene was calculated using cufflinks, and the read counts of each gene were obtained by htseq-count. FPKM and the read count value of each transcript (protein coding) were calculated using Bowtie2 and eXpress. DEGs were identified using the DESeq. R package functions estimateSizeFactors and nbinomTest. p-Value <0.05 and foldChange >2 or foldChange <0.5 were set as the threshold for significantly differential expression. Hierarchical cluster analysis of DEGs was performed to explore gene expression pattern. Gene Ontology (GO) enrichment and KEGG pathway enrichment analysis of DEGs were performed using R based on the hypergeometric distribution.

### Preparation of Single-Cell Suspensions

According to the histological observation, as shown in **Figure 1**, three uterine horns from each phase were sampled and transferred into Roswell Park Memorial Institute (RPMI) 1640 medium (Gibco, Shanghai, China) containing 10% fetal bovine serum (FBS) (Gibco, Shanghai, China) on ice. the uterine horn was then washed with PBS three times and transferred to pre-warmed digestion medium containing 100 mg/ml of DNase I (Sigma-Aldrich, Shanghai, China) and 0.1 g/ml of collagenase I (Sigma-Aldrich, Shanghai, China) in RPMI1640. The amount of enzyme used depended upon the size of the uterine horn: 1 ml of enzyme mix was added for 1 cm^2^ of tissue. Tissues were shaken vigorously for 30 s and further incubated at 37°C for 30 min in an incubator, with general shaking every 6 min to release cells. The released cells passed through a 70-mm cell strainer (BD, Shanghai, China) and were collected in 15-ml tubes containing 4 ml of fluorescence-activated cell sorting (FACS) buffer to neutralize enzymes. Cells were then collected by spinning at 500 g for 6 min and suspended in FACS buffer [1× PBS with 1% bovine serum albumin (BSA)] for subsequent staining.

### Single-Cell RNA Sequencing and Data Analysis

With the use of single-cell 3′ Library and Gel Bead Kit V3 (10x Genomics) and Chromium Single Cell B Chip Kit (10x Genomics), the cell suspension (300–600 living cells per microliter as determined by Count Star) was loaded onto the Chromium single-cell controller (10x Genomics) to generate single-cell gel beads in the emulsion according to the manufacturer’s protocol. In brief, single cells were suspended in PBS containing 0.04% BSA, and approximately 13,544 cells were added to each channel; the target cells recovered were estimated to be about 1,138 cells. Captured cells were lysed, and the released RNA was barcoded through reverse transcription in individual GEMs. Reverse transcription was performed on a S1000TM Touch Thermal Cycler (Bio-Rad, Shanghai, China) at 53°C for 45 min, followed by 85°C for 5 min, and held at 4°C. The cDNA was generated and then amplified, and quality was assessed using an Agilent 4200 (performed by CapitalBio Technology, Beijing, China). According to the manufacturer’s instructions, scRNA-seq libraries were constructed using a Single Cell 3′ Library and Gel Bead Kit V3. The libraries were ultimately sequenced using an Illumina Novaseq6000 sequencer with a sequencing depth of at least 48,594 reads per cell with a paired-ends 150 bp (PE150) reading strategy (performed by CapitalBio Technology, Beijing, China).

The Cell Ranger software was obtained from the 10x Genomics website https://support.10xgenomics.com/single-cell-gene-expression/software/downloads/latest. Alignment, filtering, barcode counting, and UMI counting were performed with a cellranger count module to generate feature-barcode matrix and to determine clusters. Dimensionality reduction was performed using principal component analysis (PCA), and the first 10 principal components were used to generate clusters by a K-means algorithm and graph-based algorithm. GO enrichment and KEGG enrichment of cluster markers were performed using KOBAS software with the Benjamini–Hochberg multiple-testing adjustment, using the top 20 marker genes of the cluster. The results were visualized using the R package.

Our single-cell trajectories were built with Monocle (R package), which introduced pseudotime. Genes were filtered by the following criteria: expression in more than 10 cells; an average expression value greater than 0.1; and Qval less than 0.01 in different analyses.

Weighted correlation network analysis (WGCNA) was performed using the WGCNA R software package; and according to clustering results, every cluster was divided into sub-clusters, and the average expression of a gene in a sub-cluster was calculated. Parameters were set to default. Cell types were annotated by singleR (https://bioconductor.org/packages/devel/bioc/html/SingleR.html), which performs unbiased cell-type recognition from scRNA-seq data by leveraging reference transcriptomic datasets of pure cell types and inferring the cell of origin for each single cell independently.

### Immunohistochemistry

The 4% paraformaldehyde-fixed uteri (n = 12 for each phase) were trimmed and dehydrated using 30% and 50% sucrose solution, respectively, and then embedded in optimal cutting temperature compound (Sakura, CA, USA), and frozen sections were cut at 5 µm. The frozen sections with distilled water were washed and then treated with 3% hydrogen peroxide for 15 min at room temperature. After being washed with PBS, the sections were treated with goat serum at room temperature for 30 min and then incubated with the indicated antibodies (**Table S1**) at 4°C overnight. After being washed three times in PBS, the sections were stained with the corresponding secondary antibodies (horseradish peroxidase (HRP)-conjugated goat anti-mouse antibody [1:200, Abcam, Shanghai, China] or HRP-conjugated goat anti-rabbit antibody [1:200, Abcam, Shanghai, China]) for 2 h at room temperature. After being washed three times in PBS, the sections were visualized with diaminobenzidine staining for 10 min at room temperature and counterstained with hematoxylin. The sections were ultimately dehydrated and mounted with neutral balsam. The numbers of macrophages in the PU and SU were counted under microscope at ×400 magnification by one investigator. According to the integrated optical density (IOD), the expressions of PCNA in the PU and SU were collected using Image-Pro Plus 9.0 under the microscope magnified at ×200. The sections of each sample in 10 random sights were selected to conduct the IOD analysis by only one investigator to minimize bias.

### 16S rRNA Full-Length Sequencing and Data Analysis

As shown in **Figure 7B**, both ends of the cornua uteri were ligated (n = 12 for each phase) and injected into the cervix with 50 ml of sterile PBS. The PBS washes were collected, and 20 ml was filtered using a 0.22-μm bacterial membrane filter to obtain the colonizing bacteria. Total microbial genomic DNA samples were extracted using the OMEGA DNA isolation kit (Omega, D5625-01, USA) following the manufacturer’s instructions and stored at −20°C prior to further analysis. The quantity and quality of extracted DNAs were measured using a NanoDrop ND-1000 spectrophotometer (Thermo Fisher Scientific, MA, USA) and agarose gel electrophoresis, respectively. The extracted DNA was amplified with two-step PCR, with sample-specific 16-bp barcodes incorporated into the forward and reverse primers for multiplex sequencing in the second PCR step. PCR amplification of the full-length bacterial 16S rRNA genes was performed using the forward primer 27F (5′-AGAGTTTGATCMTGGCTCAG-3′) and the reverse primer 1492R (5′-ACCTTGTTACGACTT-3′).

All PCR amplicons were purified with Agencourt AMPure Beads (Beckman Coulter, Indianapolis, IN) and quantified using the PicoGreen dsDNA Assay Kit (Invitrogen, CA, USA). Raw sequences were initially processed through the PacBio SMRT Link portal (version 5.0.1.9585), and sequences were filtered for a minimum of three passes, with a minimal predicted accuracy of 99% (minfullpass = 3, minPredictedAccuracy = 99). The predicted accuracy of 99% was defined as the threshold below, and a CCS was considered to be noise. The files generated by the PacBio platform were then used for amplicon size trimming to remove sequences with lengths longer than 2,000 bp.

The Quantitative Insights Into Microbial Ecology (QIIME, v1.8.0) pipeline was employed to process the sequencing data as previously described (42–44). Sequence data analyses were primarily performed using QIIME and R packages (v3.2.0). Operational taxonomic unit (OTU)-level alpha diversity indices (such as Chao1 richness estimator, ACE metric [Abundance-based Coverage Estimator], Shannon diversity index, and Simpson index) were calculated using the OTU table in QIIME. OTU-level ranked abundance curves were generated to compare the richness and evenness of OTUs among samples. Beta-diversity analysis was performed to investigate the structural variation of microbial communities across samples using UniFrac distance metrics (45) and visualized *via* principal coordinates analysis (PCoA), non-metric multidimensional scaling (NMDS), and the unweighted pair-group method with arithmetic mean (UPGMA) for hierarchical clustering (46). Differences in the UniFrac distances for pairwise comparisons among groups were determined using Student’s *t*-test and the Monte Carlo permutation test with 1,000 permutations and visualized through box-and-whiskers plots. PCA was also conducted based on genus-level compositional profiles. The significance of differentiation of microbiota structure among groups was assessed by permutational multivariate ANOVA (PERMANOVA) and analysis of similarities (ANOSIM) using the R package “vegan”. The taxonomy compositions and abundances were visualized using MEGAN and GraPhlAn. A Venn diagram was generated to visualize the shared and unique OTUs among samples or groups using the R package “VennDiagram,” based on the occurrence of OTUs across samples/groups regardless of their relative abundance. Taxa abundances at the phylum, class, order, family, genus, and species levels were statistically compared among samples or groups using Metastats and visualized as violin plots. LEfSe was used to detect differentially abundant taxa across groups using the default parameters. Partial least squares discriminant analysis (PLS-DA) was also introduced as a supervised model to reveal the variations in microbiotas among groups, using the “plsda” function in the R package “mixOmics.” Random forest analysis was applied in order to discriminate the samples from different groups using the R package “randomForest,” with 1,000 trees and all default settings. The generalization error was estimated using a 10-fold cross-validation; and the expected “baseline” error was also included, which was obtained by a classifier that simply predicted the most common category label. Co-occurrence analysis was performed by calculating Spearman’s rank correlations between/among predominant taxa. Correlations with |RHO| > 0.6 and p < 0.01 were visualized as a co-occurrence network using Cytoscape. Microbial functions with PICRUSt (phylogenetic investigation of communities by reconstruction of unobserved states) was predicted based on high-quality sequences.

### Isolation of Primary Uterine Mucosal Epithelial Cells

After anesthetization, three SPF-weaned pigs were sacrificed, and their uteri were sampled to dissociate the primary uterine MEC. The uterine mucosa was dissected and washed in sterile PBS three times at room temperature to remove mucus. Specimens were digested for 2 h in 0.1 mg/ml of collagenase A solution, and the digests were filtered through a cell strainer and then were centrifuged for 15 min at 500 g. The cell pellets were re-suspended with modified DMEM/F12 medium (50 ml of fetal bovine serum, 1.25 ml of 1,000 mg/L insulin, 50 μl of 100 g/ml EGF, 2 ml of 25 mg/ml heparin sodium, and 10 ml of penicillin–streptomycin in 450 ml of medium). The primary uterine MEC were purified according to the various digestion and adherence times during their passages in culture.

### Isolation of Splenic Lymphocytes

Three SPF-weaned pigs were anesthetized and sacrificed, and their spleens were sampled. After the tunica was removed, the spleens were washed three times with sterile PBS and then cut into pieces. The pieces were transferred to a stainless cell strainer incubated in RPMI1640 medium and then ground to obtain single-cell suspensions. After filtration through the cell strainer, the suspensions were centrifuged at 400 g, and the cell pellets were re-suspended and washed with sterile PBS. After being washed three times, the cellular pellet was re-suspended with lysis buffer to discard the red blood cells; and after centrifugation, the cell pellet was re-suspended and washed twice with RPMI1640 medium. The lymphocytes were then cultured in the modified RPMI1640 medium with 10% FBS, and 200 IU of penicillin–streptomycin was added for the subsequent assay.

### Co-Culture Assay and siRNA Transfection

In order to analyze the modulation mechanism of lymphocyte migration, a co-culture assay was performed for UMEC to recruit splenic lymphocyte migration. Transwell plates were chosen, and then the Matrigel (1:8 in PBS) was prepared on the bottom of the upper cell in 37°C for 30 min. After being hydrated in PBS for 10 min at room temperature, the UMEC were cultured in the lower chamber and exposed to LPS (1 μg/ml) or 800 μl of collected colonizing bacteria. The colonizing bacteria were collected as described above and shown in **Figure 7B**; both ends of the cornua uteri were ligated, and 50 ml of sterile PBS was injected into the cervix. All the washes were collected, and 20 ml of PBS was filtered using a 0.22-μm bacterial membrane. Then the membrane was washed in 5 ml of sterile PBS at room temperature on rotary shaker for 1 h to obtain the colonizing bacteria. At 24 and 48 h after exposure, splenic lymphocytes were added in the upper cell, and then the numbers of migrated lymphocytes were counted at 24 and 48 h, respectively. To detect gene expressions by UMEC and migrated lymphocytes, the UMEC and lymphocytes at 24 h and 48 h were collected, and then total RNA for quantitative real-time PCR was prepared as described above.

To investigate the role of CCL4 in the modulation of lymphocyte migration, as shown in **Figure 7**, the UMEC were transfected with interfering RNA (RNAi) to downregulate *CCL4* expression, then were cultured in the bottom of the lower well, and exposed to LPS or colonizing bacteria collected from 0.22-μm bacterial membrane filtration. At 24 and 48 h after exposure, the splenic lymphocytes were cultured in the upper cell, and the numbers of migrated lymphocytes were counted at 24 and 48 h.

### RNA Extraction and Quantitative Real-Time PCR

Total RNA was extracted according to the manufacturer’s instructions using the TRIZOL^®^ reagent (Invitrogen, CA, USA). RNA (0.5 µg) was transcribed into cDNA using the Fast Quant RT Kit (with gDNase) (TIANGEN Biotech Co. Ltd, Beijing, China) according to the manufacturer’s instructions. The expression levels of the genes were quantified with quantitative real-time PCR (RT-qPCR) using the SYBR Green Real-time PCR Master Mix (TIANGEN Biotech Co. Ltd, Beijing, China). The primers were designed using the Primer Premier 5 software (PREMIER Biosoft, Palo Alto, CA) and were subsequently synthesized (Sangon Biotech Co. Ltd, Beijing, China). The detected genes and primers are listed in **Table S9**. The cycling parameters used for qPCR amplification were as follows: initial heat denaturation at 95°C for 4 min; 40 cycles of 95°C for 30 s, 59°C for 30 s, and 72°C for 30 s; and a final extension at 72°C for 5 min. A melting curve analysis was performed to exclude genomic DNA contamination and to confirm primer specificities. Gene expression was normalized using the 2^−ΔΔCT^ method with the glyceraldehyde 3-phosphate dehydrogenase (*GAPDH*) gene used as an internal standard. Each biological duplicate was controlled in three technical replicates.

### Statistical Analysis

All statistical analyses were performed using Prism 9.0 (GraphPad Software). Data are expressed as means ± standard error of the mean (SEM). Statistical significance was evaluated using Student’s *t*-test. Asterisk coding is indicated in the figure legends: *, # indicate *p* < 0.05; and **, ## indicate *p* < 0.01.

## Data Availability Statement

The original contributions presented in the study are included in the article/**Supplementary Material**. The datasets supporting the conclusions of this article are available in the NCBI Sequence Read Archive under accession number PRJNA600817 and PRJNA601123.

## Ethics Statement

The animal study was reviewed and approved by Beijing Laboratory Animal Welfare and Ethics Committee, Beijing Association for Science and Technology. Written informed consent was obtained from the owners for the participation of their animals in this study.

## Author Contributions

DH conceived the project and designed the steady-state experiments. DH, PS, JW, GH, CS, and FT collected the samples for RNA-seq and scRNA-seq. DH and GH collected the samples for 16S rRNA sequencing. DH, PS, JFC, GH, YT, and XY planned the data analyses. DH performed the immunohistochemistry. DH, PS, and JC performed the primary cell isolation and *in vitro* experiment. JW, YH, JYC, and YM provided the resources. DH wrote the manuscript, and DH and HD revised the manuscript. DH and YM supervised the work and edited manuscripts. All authors contributed to the article and approved the submitted version.

## Funding

This work was supported by grants from the National Natural Science Foundation of China: 31772686 and 21027094.

## Conflict of Interest

PS was employed by the company Shandong Hekangyuan Biological Breeding Co., Ltd.

The remaining authors declare that the research was conducted in the absence of any commercial or financial relationships that could be construed as a potential conflict of interest.

## Publisher’s Note

All claims expressed in this article are solely those of the authors and do not necessarily represent those of their affiliated organizations, or those of the publisher, the editors and the reviewers. Any product that may be evaluated in this article, or claim that may be made by its manufacturer, is not guaranteed or endorsed by the publisher.

## References

[1] Zhang S Kong S Wang B Cheng X Chen Y Wu W. Uterine Rbpj is Required for Embryonic-Uterine Orientation and Decidual Remodeling *via* Notch Pathway-Independent and -Dependent Mechanisms. Cell Res (2014) 24(8):925–42. doi: 10.1038/cr.2014.82 PMC4123295 24971735

[2] Vento-Tormo R Efremova M Botting RA Turco MY Vento-Tormo M Meyer KB. Single-Cell Reconstruction of the Early Maternal-Fetal Interface in Humans. Nature (2018) 563(7731):347–53. doi: 10.1038/s41586-018-0698-6 PMC7612850 30429548

[3] Niu Y Sun N Li C Lei Y Huang Z Wu J. Dissecting Primate Early Post-Implantation Development Using Long-Term *In Vitro* Embryo Culture. Science (2019) 366(6467):eaaw5754. doi: 10.1126/science.aaw5754 31672917

[4] Li R Zhong C Yu Y Liu H Sakurai M Yu L. Generation of Blastocyst-Like Structures From Mouse Embryonic and Adult Cell Cultures. Cell (2019) 179(3):687–702 e18. doi: 10.1016/j.cell.2019.09.029 31626770 PMC7359735

[5] Griffith OW Chavan AR Protopapas S Maziarz J Romero R Wagner GP. Embryo Implantation Evolved From an Ancestral Inflammatory Attachment Reaction. Proc Natl Acad Sci USA (2017) 114(32):E6566–75. doi: 10.1073/pnas.1701129114 PMC5559003 28747528

[6] Pennisi E. Tamed Immune Reaction Aids Pregnancy. Science (2018) 359(6373):260. doi: 10.1126/science.359.6373.260 29348216

[7] Kelleher AM Milano-Foster J Behura SK Spencer TE. Uterine Glands Coordinate on-Time Embryo Implantation and Impact Endometrial Decidualization for Pregnancy Success. Nat Commun (2018) 9(1):2435. doi: 10.1038/s41467-018-04848-8 29934619 PMC6015089

[8] Raine T Liu JZ Anderson CA Parkes M Kaser A. Generation of Primary Human Intestinal T Cell Transcriptomes Reveals Differential Expression at Genetic Risk Loci for Immune-Mediated Disease. Gut (2015) 64(2):250–9. doi: 10.1136/gutjnl-2013-306657 PMC4316924 24799394

[9] Hoytema van Konijnenburg DP Reis BS Pedicord VA Farache J Victora GD Mucida D. Intestinal Epithelial and Intraepithelial T Cell Crosstalk Mediates a Dynamic Response to Infection. Cell (2017) 171(4):783–94 e13. doi: 10.1016/j.cell.2017.08.046 28942917 PMC5670000

[10] Olivares-Villagomez D Van Kaer L. Intestinal Intraepithelial Lymphocytes: Sentinels of the Mucosal Barrier. Trends Immunol (2018) 39(4):264–75. doi: 10.1016/j.it.2017.11.003 PMC8056148 29221933

[11] Mayassi T Jabri B. Human Intraepithelial Lymphocytes. Mucosal Immunol (2018) 11(5):1281–9. doi: 10.1038/s41385-018-0016-5 PMC6178824 29674648

[12] Li Y Innocentin S Withers DR Roberts NA Gallagher AR Grigorieva EF. Exogenous Stimuli Maintain Intraepithelial Lymphocytes *via* Aryl Hydrocarbon Receptor Activation. Cell (2011) 147(3):629–40. doi: 10.1016/j.cell.2011.09.025 21999944

[13] McDonald BD Jabri B Bendelac A. Diverse Developmental Pathways of Intestinal Intraepithelial Lymphocytes. Nat Rev Immunol (2018) 18(8):514–25. doi: 10.1038/s41577-018-0013-7 PMC6063796 29717233

[14] Huang C Zeng Y Tu W. The Role of Gammadelta-T Cells During Human Pregnancy. Am J Reprod Immunol (2017) 78(2):e12713. doi: 10.1111/aji.12713 28653491

[15] Bush A Fleming L Saglani S. Severe Asthma in Children. Respirology (2017) 22(5):886–97. doi: 10.1111/resp.13085 28543931

[16] Gamliel M Goldman-Wohl D Isaacson B Gur C Stein N Yamin R. Trained Memory of Human Uterine NK Cells Enhances Their Function in Subsequent Pregnancies. Immunity (2018) 48(5):951–62.e5. doi: 10.1016/j.immuni.2018.03.030 29768178

[17] Kobayashi T Voisin B Kim DY Kennedy EA Jo JH Shih HY. Homeostatic Control of Sebaceous Glands by Innate Lymphoid Cells Regulates Commensal Bacteria Equilibrium. Cell (2019) 176(5):982–97.e16. doi: 10.1016/j.cell.2018.12.031 30712873 PMC6532063

[18] Altmäe S Koel M Võsa U Adler P Suhorutšenko M Laisk-Podar T. Meta-Signature of Human Endometrial Receptivity: A Meta-Analysis and Validation Study of Transcriptomic Biomarkers. Sci Rep (2017) 7(1):10077. doi: 10.1038/s41598-017-10098-3 28855728 PMC5577343

[19] Wang W Vilella F Alama P Moreno I Mignardi M Isakova A. Single-Cell Transcriptomic Atlas of the Human Endometrium During the Menstrual Cycle. Nat Med (2020) 26(10):1644–53. doi: 10.1038/s41591-020-1040-z 32929266

[20] Ladinsky MS Araujo LP Zhang X Veltri J Galan-Diez M Soualhi S. Endocytosis of Commensal Antigens by Intestinal Epithelial Cells Regulates Mucosal T Cell Homeostasis. Science (2019) 363(6431):eaat4042. doi: 10.1126/science.aat4042 30846568 PMC6708280

[21] Qi X Yun C Sun L Xia J Wu Q Wang Y. Gut Microbiota-Bile Acid-Interleukin-22 Axis Orchestrates Polycystic Ovary Syndrome. Nat Med (2019) 25(8):1225–33. doi: 10.1038/s41591-019-0509-0 PMC7376369 31332392

[22] Cervantes-Barragan L Chai JN Tianero MD Di Luccia B Ahern PP Merriman J. Lactobacillus Reuteri Induces Gut Intraepithelial CD4(+)CD8alphaalpha(+) T Cells. Science (2017) 357(6353):806–10. doi: 10.1126/science.aah5825 PMC5687812 28775213

[23] Duerkop BA Vaishnava S Hooper LV. Immune Responses to the Microbiota at the Intestinal Mucosal Surface. Immunity (2009) 31(3):368–76. doi: 10.1016/j.immuni.2009.08.009 19766080

[24] Peng J Sun BF Chen CY Zhou JY Chen YS Chen H. Single-Cell RNA-Seq Highlights Intra-Tumoral Heterogeneity and Malignant Progression in Pancreatic Ductal Adenocarcinoma. Cell Res (2019) 29(9):725–38. doi: 10.1038/s41422-019-0195-y PMC6796938 31273297

[25] Sujino T London M Hoytema van Konijnenburg DP Rendon T Buch T Silva HM. Tissue Adaptation of Regulatory and Intraepithelial CD4(+) T Cells Controls Gut Inflammation. Science (2016) 352(6293):1581–6. doi: 10.1126/science.aaf3892 PMC4968079 27256884

[26] Kadowaki A Miyake S Saga R Chiba A Mochizuki H Yamamura T. Gut Environment-Induced Intraepithelial Autoreactive CD4(+) T Cells Suppress Central Nervous System Autoimmunity *via* LAG-3. Nat Commun (2016) 7:11639. doi: 10.1038/ncomms11639 27198196 PMC4876462

[27] Nielsen MM Witherden DA Havran WL. Gammadelta T Cells in Homeostasis and Host Defence of Epithelial Barrier Tissues. Nat Rev Immunol (2017) 17(12):733–45. doi: 10.1038/nri.2017.101 PMC5771804 28920588

[28] Kurd N Robey EA. Unconventional Intraepithelial Gut T Cells: The TCR Says it All. Immunity (2014) 41(2):167–8. doi: 10.1016/j.immuni.2014.08.004 25148016

[29] Zhou X Yu J Cheng X Zhao B Manyam GC Zhang L. The Deubiquitinase Otub1 Controls the Activation of CD8(+) T Cells and NK Cells by Regulating IL-15-Mediated Priming. Nat Immunol (2019) 20(7):879–89. doi: 10.1038/s41590-019-0405-2 PMC6588407 31182807

[30] Wang Y Zhang Y Yi P Dong W Nalin AP Zhang J. The IL-15-AKT-XBP1s Signaling Pathway Contributes to Effector Functions and Survival in Human NK Cells. Nat Immunol (2019) 20(1):10–7. doi: 10.1038/s41590-018-0265-1 PMC6293989 30538328

[31] Sun M Wu W Chen L Yang W Huang X Ma C. Microbiota-Derived Short-Chain Fatty Acids Promote Th1 Cell IL-10 Production to Maintain Intestinal Homeostasis. Nat Commun (2018) 9(1):3555. doi: 10.1038/s41467-018-05901-2 30177845 PMC6120873

[32] Morita N Umemoto E Fujita S Hayashi A Kikuta J Kimura I. GPR31-Dependent Dendrite Protrusion of Intestinal CX3CR1(+) Cells by Bacterial Metabolites. Nature (2019) 566(7742):110–4. doi: 10.1038/s41586-019-0884-1 30675063

[33] Ardain A Domingo-Gonzalez R Das S Kazer SW Howard NC Singh A. Group 3 Innate Lymphoid Cells Mediate Early Protective Immunity Against Tuberculosis. Nature (2019) 570(7762):528–32. doi: 10.1038/s41586-019-1276-2 PMC6626542 31168092

[34] Zhao Y Chen F Wu W Sun M Bilotta AJ Yao S. GPR43 Mediates Microbiota Metabolite SCFA Regulation of Antimicrobial Peptide Expression in Intestinal Epithelial Cells *via* Activation of mTOR and STAT3. Mucosal Immunol (2018) 11(3):752–62. doi: 10.1038/mi.2017.118 PMC5976519 29411774

[35] Jin C Lagoudas GK Zhao C Bullman S Bhutkar A Hu B. Commensal Microbiota Promote Lung Cancer Development *via* Gammadelta T Cells. Cell (2019) 176(5):998–1013 e16. doi: 10.1016/j.cell.2018.12.040 30712876 PMC6691977

[36] Owusu-Akyaw A Krishnamoorthy K Goldsmith LT Morelli SS. The Role of Mesenchymal-Epithelial Transition in Endometrial Function. Hum Reprod Update (2019) 25(1):114–33. doi: 10.1093/humupd/dmy035 30407544

[37] Lee K Jeong J Kwak I Yu CT Lanske B Soegiarto DW. Indian Hedgehog is a Major Mediator of Progesterone Signaling in the Mouse Uterus. Nat Genet (2006) 38(10):1204–9. doi: 10.1038/ng1874 16951680

[38] Kulkarni S Lied A Kulkarni V Rucevic M Martin MP Walker-Sperling V. CCR5AS lncRNA Variation Differentially Regulates CCR5, Influencing HIV Disease Outcome. Nat Immunol (2019) 20(7):824–34. doi: 10.1038/s41590-019-0406-1 PMC6584055 31209403

[39] Gosmann C Anahtar MN Handley SA Farcasanu M Abu-Ali G Bowman BA. Lactobacillus-Deficient Cervicovaginal Bacterial Communities Are Associated With Increased HIV Acquisition in Young South African Women. Immunity (2017) 46(1):29–37. doi: 10.1016/j.immuni.2016.12.013 28087240 PMC5270628

[40] Van Gorp H Van Breedam W Van Doorsselaere J Delputte PL Nauwynck HJ. Identification of the CD163 Protein Domains Involved in Infection of the Porcine Reproductive and Respiratory Syndrome Virus. J Virol (2010) 84(6):3101–5. doi: 10.1128/JVI.02093-09 PMC2826032 20032174

[41] Noval Rivas M Wakita D Franklin MK Carvalho TT Abolhesn A Gomez AC. Intestinal Permeability and IgA Provoke Immune Vasculitis Linked to Cardiovascular Inflammation. Immunity (2019) 51(3):508–21 e6. doi: 10.1016/j.immuni.2019.05.021 31471109 PMC6751009

[42] Caporaso JG Kuczynski J Stombaugh J Bittinger K Bushman FD Costello EK. QIIME Allows Analysis of High-Throughput Community Sequencing Data. Nat Methods (2010) 7(5):335–6. doi: 10.1038/nmeth.f.303 PMC3156573 20383131

[43] Edgar RC. Search and Clustering Orders of Magnitude Faster Than BLAST. Bioinformatics (2010) 26(19):2460–1. doi: 10.1093/bioinformatics/btq461 20709691

[44] Altschul SF Madden TL Schaffer AA Zhang J Zhang Z Miller W. Gapped BLAST and PSI-BLAST: A New Generation of Protein Database Search Programs. Nucleic Acids Res (1997) 25(17):3389–402. doi: 10.1093/nar/25.17.3389 PMC146917 9254694

[45] Lozupone C Knight R. UniFrac: A New Phylogenetic Method for Comparing Microbial Communities. Appl Environ Microbiol (2005) 71(12):8228–35. doi: 10.1128/AEM.71.12.8228-8235.2005 PMC1317376 16332807

[46] Ramette A. Multivariate Analyses in Microbial Ecology. FEMS Microbial Ecol (2007) 62(2):142–60. doi: 10.1111/j.1574-6941.2007.00375.x PMC2121141 17892477

