# Supplementary material for: The Immune Barrier of Porcine Uterine Mucosa Differs Dramatically at Proliferative and Secretory Phases and Could Be Positively Modulated by Colonizing Microbiota
Source: Front Immunol. 2021 Nov 30;12:750808. doi: 10.3389/fimmu.2021.750808 (PMC8670328; doi:10.3389/fimmu.2021.750808)
Supplement: Supplementary file 1 [file DataSheet_1.docx]

**Supplementary Materials**

**Supplementary Figures**

**
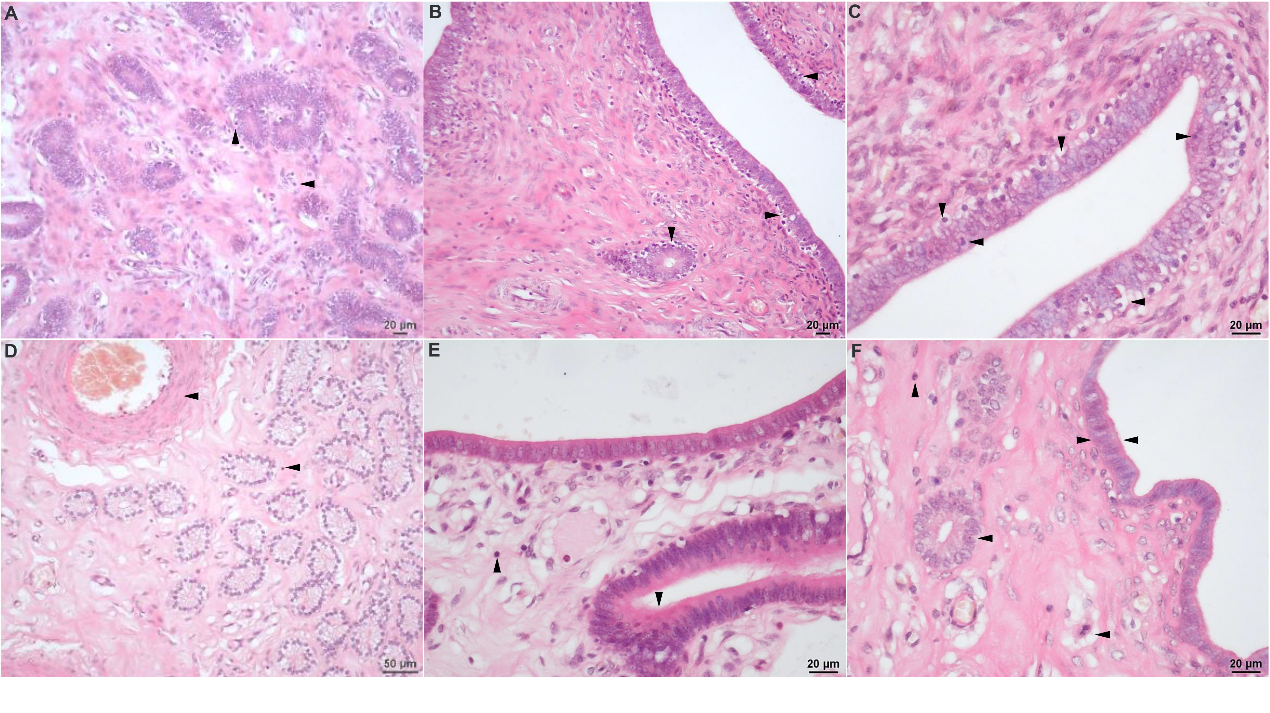
**

**FIGURE S1|** Obvious differences in mucosal immunity of endometrium between the PU and SU. (**A**) More lymphocytes distributed in the lamina propria of endometrium in the PU. (**B**) Lots of lymphocytes migrated into the mucosal and uterine glandular epithelium in the PU. (**C**) The mucosal epithelium in the PU showed pseudostratified columnar epithelium and loosely arrangement with more intraepithelial lymphocytes migration into the epithelium. (**D**) Fewer lymphocytes and increased artery blood vessels were in the endometrium of the SU. (**E-F**) The mucosal epithelial cells turned into neatly and tightly with integrated basal membrane and more secretion on the surface of the mucosal epithelium. More granulocytes were observed in the lamina propria, and more adipocytes were around the small and micro blood vessels.


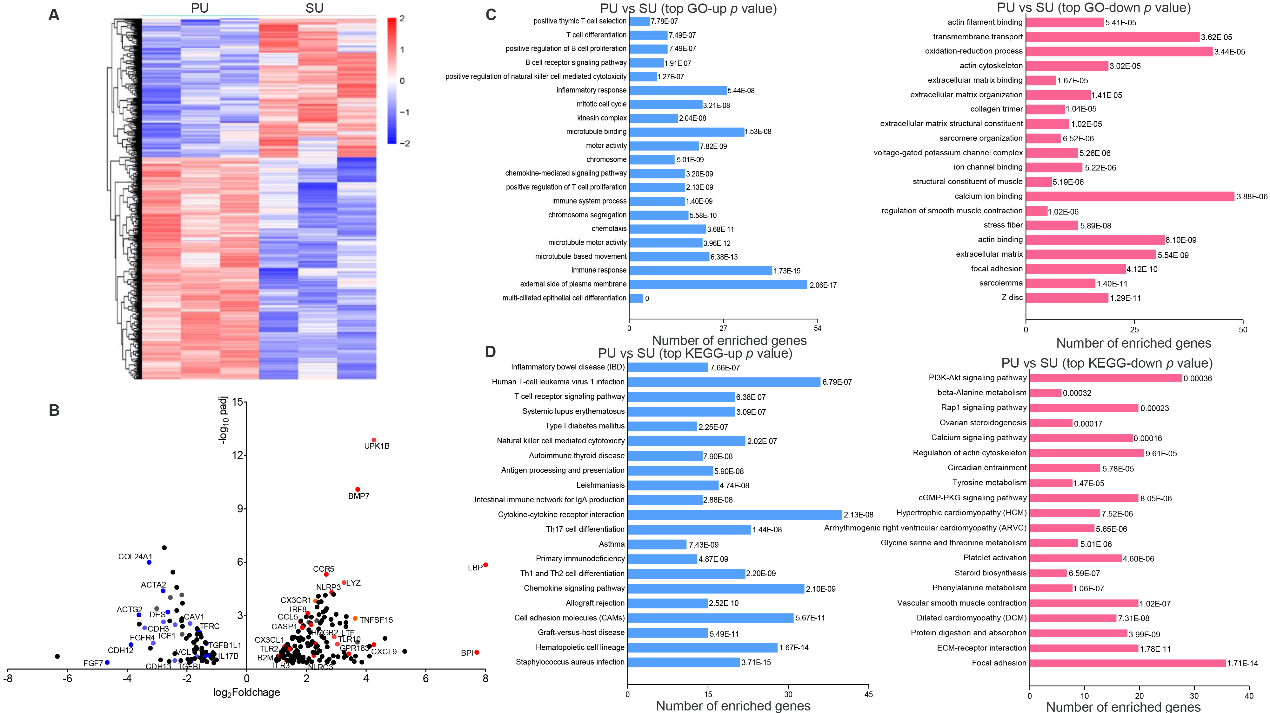


**FIGURE S2|** Differentially expressed genes (DEGs) and enriched GO terms and KEGG pathways in endometrium of the PU and SU. (**A**) Heatmap significantly showed the clustered DEGs in the endometrium between the PU and SU. (**B**) Genes related to immunity and metabolism differentially expressed in the PU (red dot) and SU (blue dot) respectively. (**C**) The enriched GO terms from up- and down-regulated genes between the PU and SU. (**D**) The KEGG pathways enriched from up- and down-regulated genes between the PU and SU.


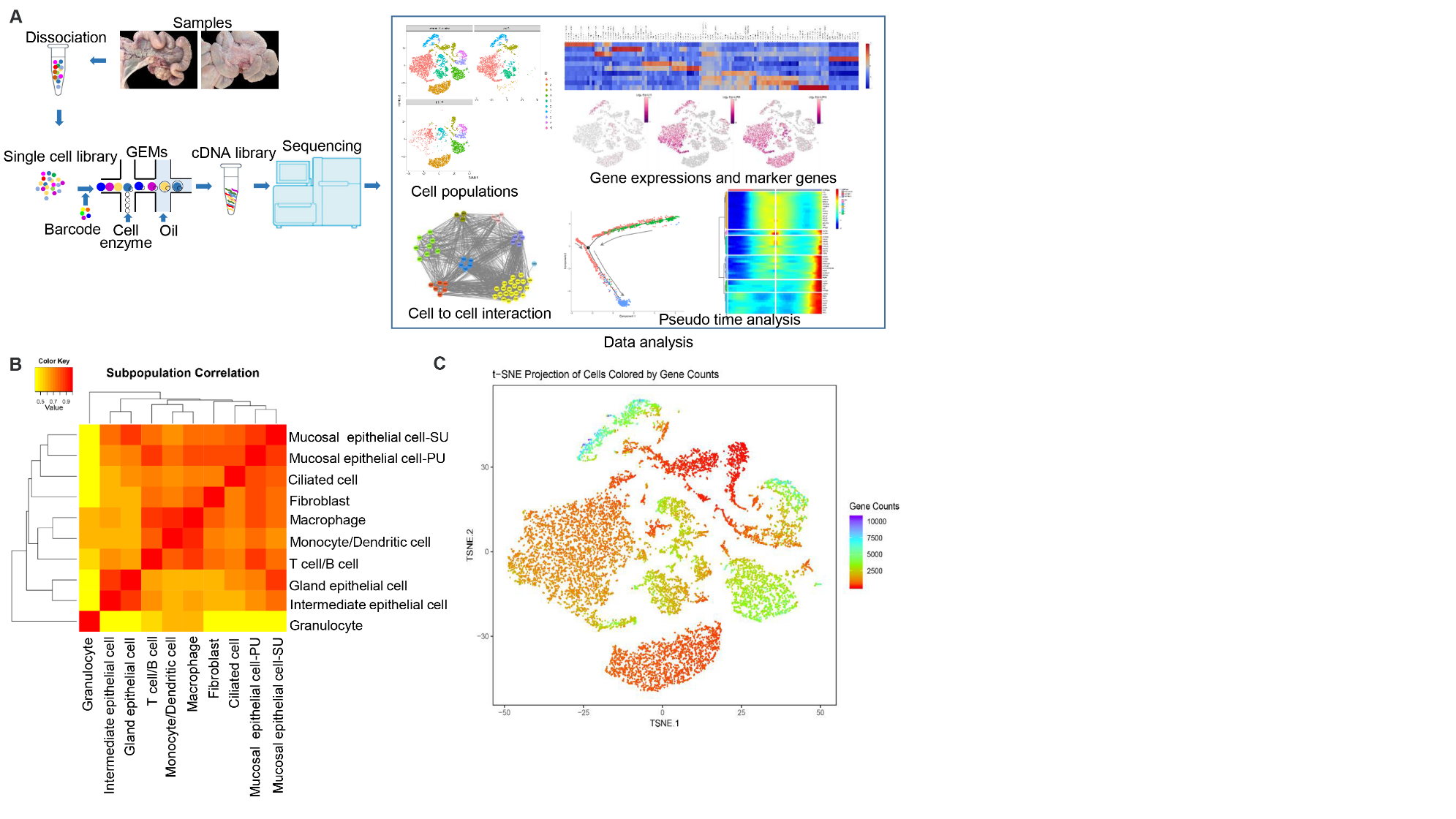


**FIGURE S3|** Single cell RNA sequencing (scRNA-seq) process, gene counts and correlation of cell populations from endometrium of the PU and SU. (**A**) The endometrium from the PU and SU were sampled and performed the scRNA-seq to analyze cell populations and genes expressions in specific cell populations. (**B**) The correlations of identified cell populations. (**C**) Genes counts were analyzed in different cell populations.


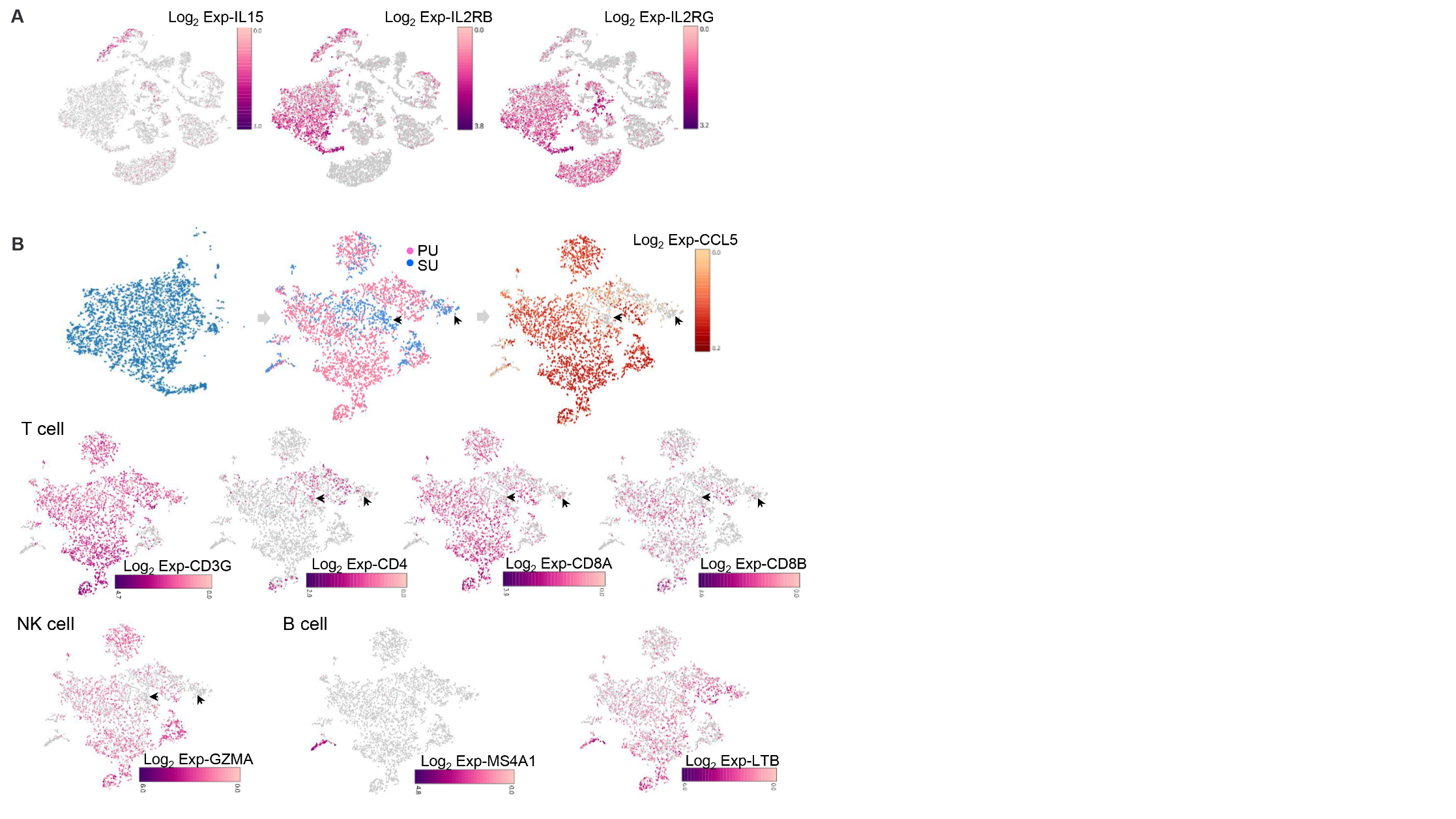


**FIGURE S4|** Genes expression in mucosal epithelial cells and sub-populations of lymphocytes in the endometrium of PU and SU. (**A**) Expression of *IL15*, *IL2RB*, and *IL2RG* in the mucosal epithelial cells and lymphocytes. (**B**) *CCL5* was highly detected in the lymphocytes from the PU and the lymphocytes were sub divided into T cells, NK cell and B cell according to specific genes expressions.

**
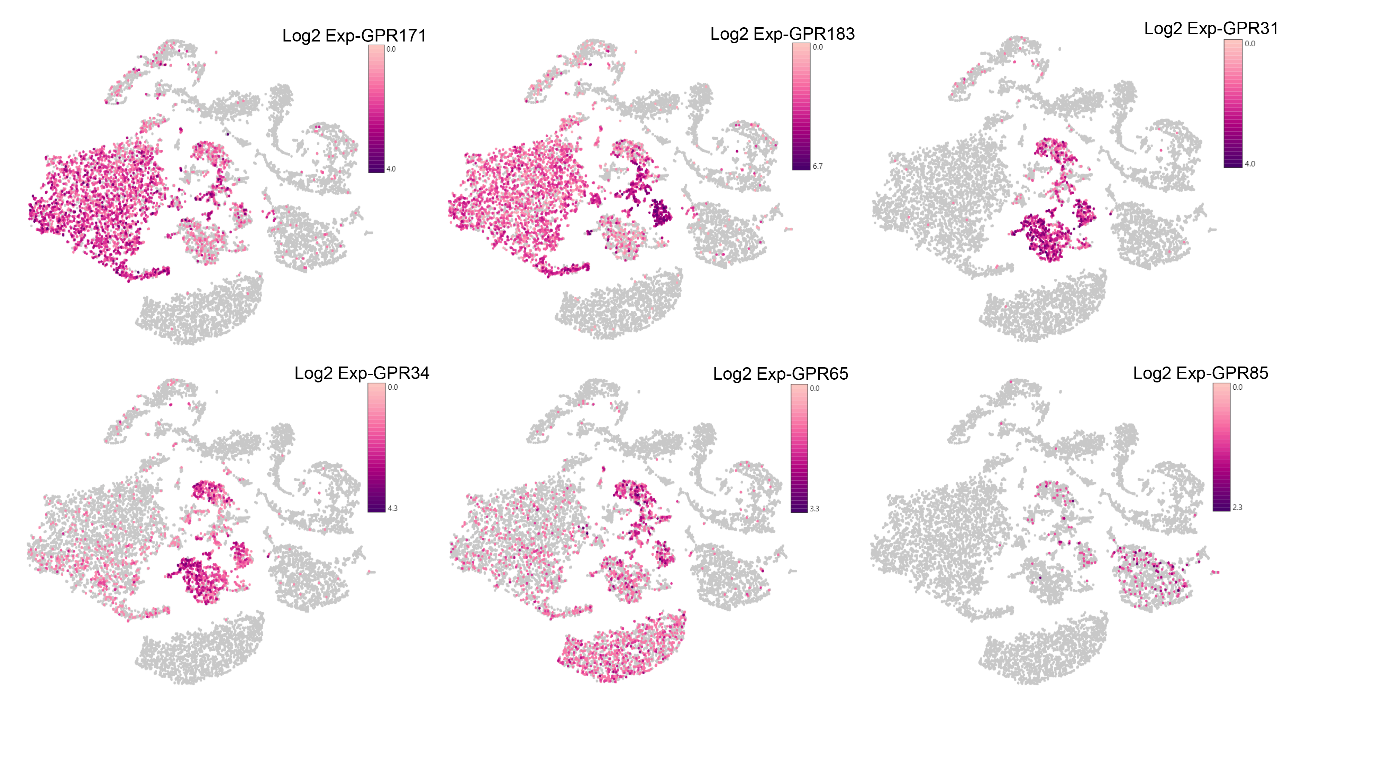
**

**FIGURE S5|** Expressions of G protein coupled receptors in different cell populations of the PU and SU. (**A**) Expression of *IL15*, *IL2RB*, and *IL2RG* in the mucosal epithelial cells and lymphocytes. (**B**) CCL5 were highly in the lymphocytes from PU. (**C**) The lymphocytes were sub divided into T cells, NK cell and B cell according to specific genes expressions.

**
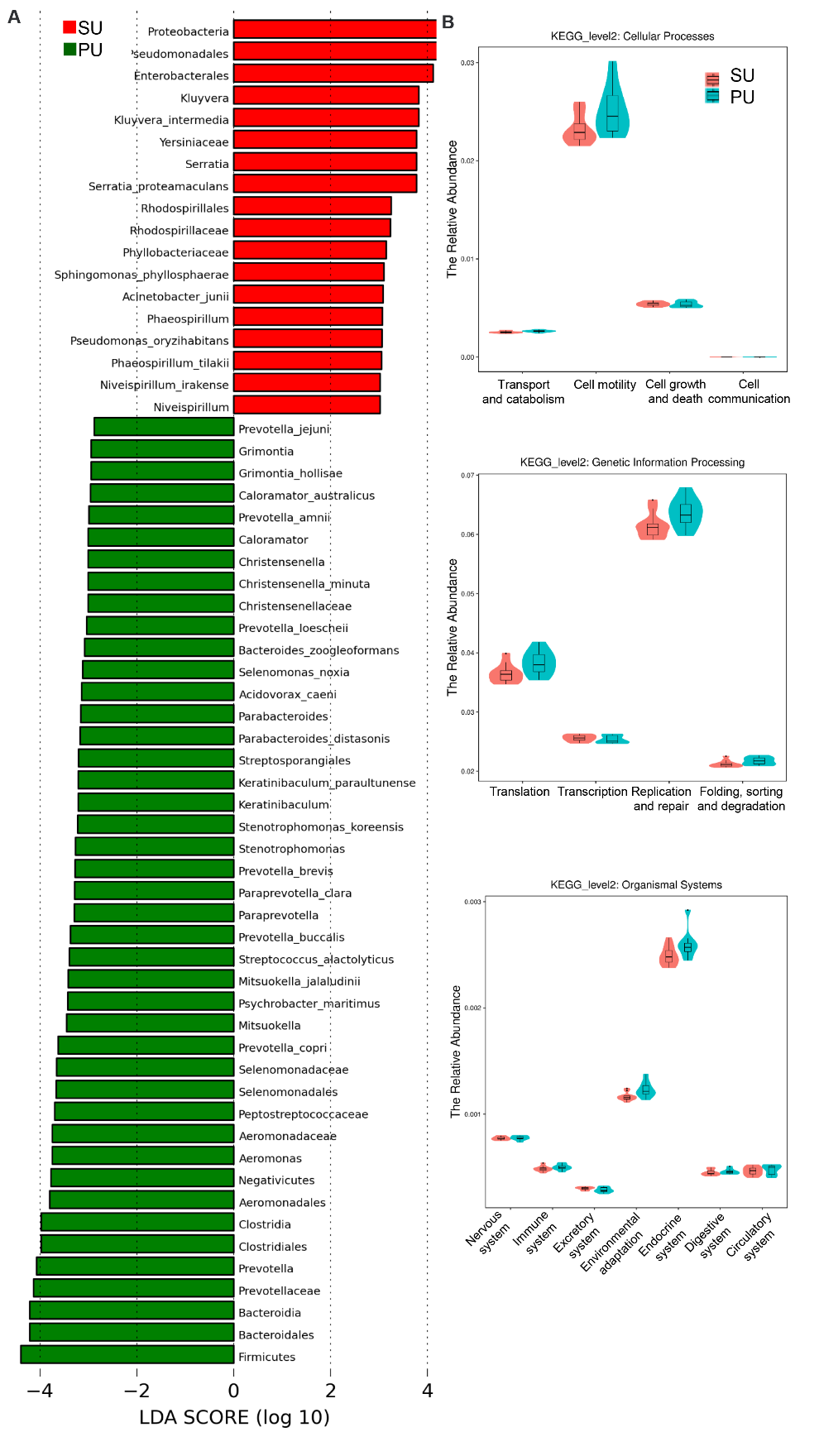
**

**FIGURE S6|** The top colonized bacteria in the PU and SU, and possible pathways identified from the bacteria. (**A**) The composition of colonized bacteria was significantly different in the PU and SU. (**B**) KEGG pathways enriched from the colonized bacteria. Cell motility, replication and repair, and endocrine system were differentially detected between the PU and SU.

**
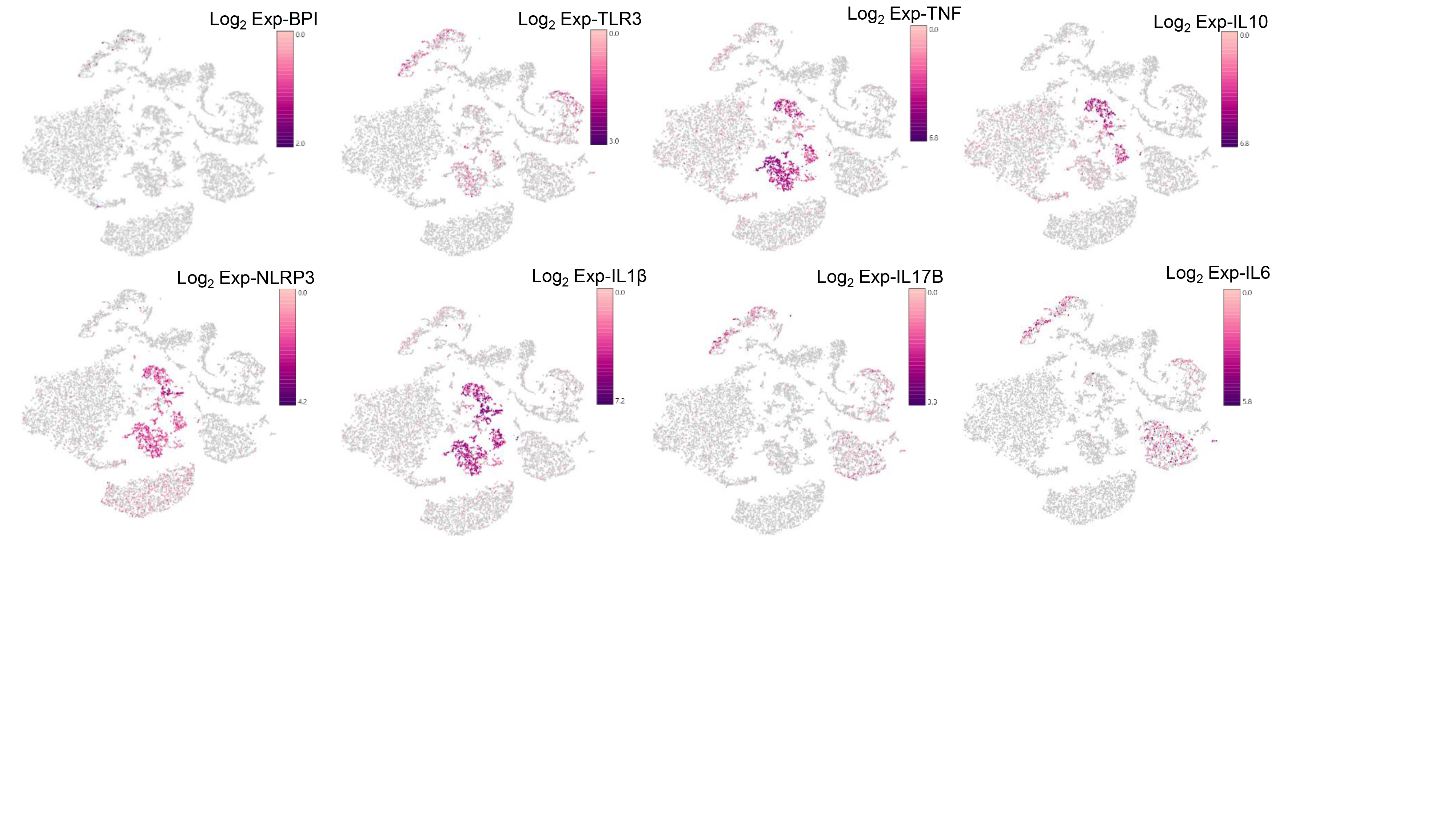
**

**FIGURE S7|** Immune genes related to bacterial infection, innate immune response and inflammatory response were in the different cell populations.

**
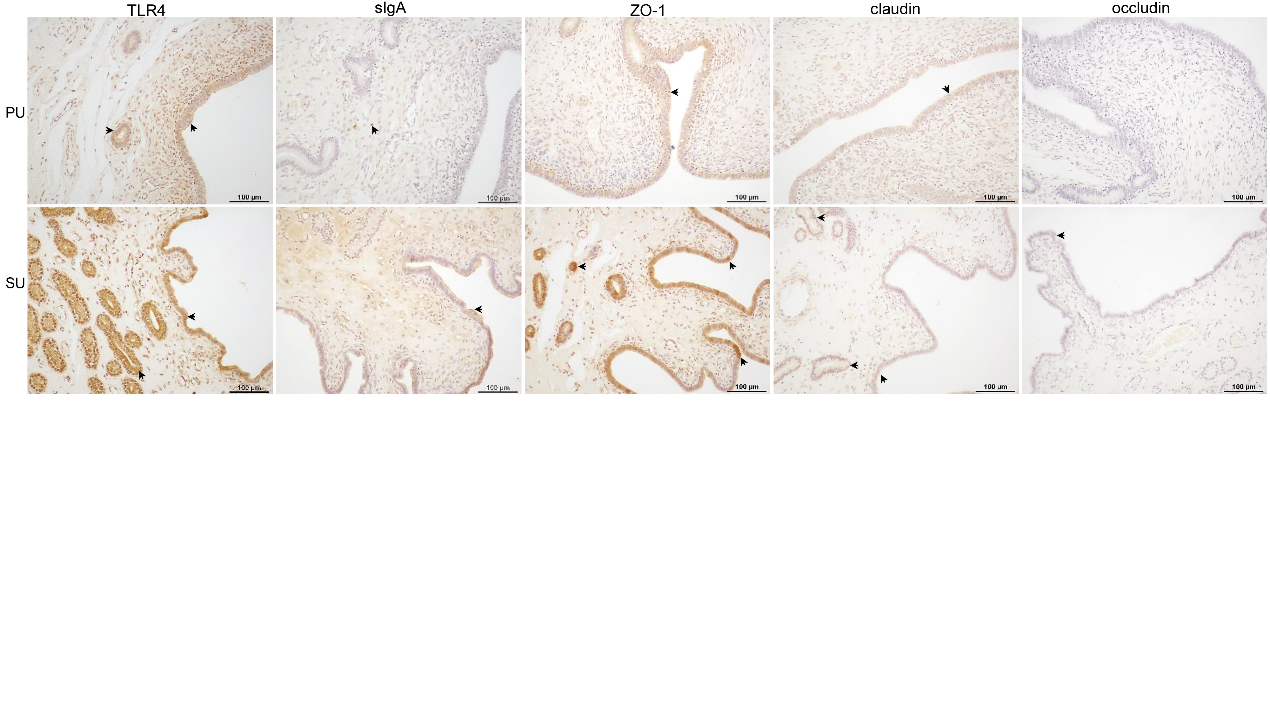
**

**FIGURE S8|** The mechanical mucosal barriers are different between the PU and SU. Higher expressions of TLR4, sIgA, ZO-1, claudin, and occludin were detected in the endometrium of the SU.

**Supplementary Tables**

**Table S1. The reagents and resources used in this study.**

| REAGENT or RESORCES | | SOURCE | IDENTIFIER |
| --- | --- | --- | --- |
| Antibodies | |  |  |
| CCL4 | | abcam | Cat#ab25129 |
| F4/80 | | abcam | Cat#ab100790 |
| PCNA | | abcam | Cat#ab19166 |
| TLR4 | | abcam | Cat#ab22048 |
| sIgA | | abcam | Cat#ab3924 |
| ZO-1 | | abcam | Cat#ab214228 |
| Claudin-1 | | abcam | Cat#ab15098 |
| occludin | | abcam | Cat#ab222691 |
| Goat anti-Rabbit IgG (HRP) | | abcam | Cat#ab6721 |
| Goat anti-Mouse IgG (HRP) | | abcam | Cat#ab6728 |
| Reagent and resources | |  |  |
| RPMI1640 Medium | | Gibco | Cat#11875093 |
| DMEM Medium | | Gibco | Cat#12320032 |
| DMEM/F12 medium | | Gibco | Cat#10565018 |
| Fetal bovine serum | | Gibco | Cat#10100147 |
| DNase I | | Sigma-Aldrich | Cat#DN25 |
| Collagenase I | | Sigma-Aldrich | Cat#C2674 |
| Cell strainer | | BD | Cat#352350 |
| EGF Recombinant Protein | | Gibco | Cat#PHG0311 |
| Heparin | | Sigma | Cat#H3149 |
| Insulin | | Sigma | Cat#91077C |
| Collagenase | | Solarbio | Cat#C8140 |
| Trypsin-EDTA | | Gibco | Cat#25200072 |
| Penicillin-Streptomycin | | Gibco | Cat#15070063 |
| DMSO | | Solarbio | Cat#D8731 |
| 6-well plates | | Corning | Cat#3335 |
| Transwell plates | | Corning | Cat#3428 |
| Matrigel basement membrane matrix | | BD | Cat#356234 |
| Trizol | | TIANGEN | Cat#DP424 |
| SYBGREEN PCR MIX | | TIANGEN | Cat#FP205 |
| Critical Commercial Assays | |  |  |
| Chromium single cell B Chip kit | | 10x Genomics | Cat#1000074 |
| Single cell 3’ Library and Gel Bead kit V3 | | 10x Genomics | Cat#1000075 |
| OMEGA DNA isolation kit | | Omega | Cat#D5635-01 |
| Nextera XT DNA Sample Preparation kit | | Illumina | Cat#FC-131-1096 |
| TruSeq Stranded mRNA LTS ample Prep Kit | | Illumina | RS-122-2101 |
| Agencourt AMPure Beads | | Beckman Coulter | A63881 |
| Qubit RNA Assay Kit | | Life Technologies | Q32852 |
| Qubit dsDNA Assay Kit | | Life Technologies | Q328520 |
| Bioanalyzer 2100 RNA-6000 Nano Kit | | Aglient | 5067-1511 |
| Bioanalyzer 2100 DNA-1000 Kit | | Aglient | 5067-1504 |
| SuperScript Ⅱ Reverse Transcriptase | | Invitrogen | 18064014 |
| Deposited Data | |  |  |
| Cell types annotation | | This paper | <https://bioconductor.org/packages/devel/bioc/html/SingleR.html> |
| Software and Algorithms | |  |  |
| R | https://www.r-project.org/ | | Version: 3.5.1; RRID: SCR_001905 |
| RStudio | https://www.rstudio.com/ | | Version: 1.2.720 |
| Cell Ranger | 10x Genomics | | Version: 1.2.0 |
| Loupe Cell Browser | 10x Genomics | | Version: 3.0.1 |
| GraphPad Prism | GraphPad Software | | Version: 6.0 |
| Other | |  |  |
| Illumina Nextera XT protocol sample B1 preparation protocol | | Fluidigm | PN 100-5950 |
| 10x library preparation protocol | | 10x Genomics | https://support.10xgenomics.com/single- cell-gene-expression/library-prep/doc/ user-guide-chromium-single-cell-3- reagent-kits-user-guide-v2-chemistry |

**Table S2. DEGs with pvalue < 0.05 and foldchange > 2 or foldChange < 0.5 between the PU and SU.**

| ***Gene name*** | **log2 FC** | ***padj*** |
| --- | --- | --- |
| *ATP6V1C2* | -5.2496035 | 4.38E-19 |
| *ERN2* | 7.3605173 | 5.75E-16 |
| *PVALB* | 5.5896612 | 2.66E-15 |
| *UPK1B* | 4.2607188 | 1.14E-13 |
| *LOC110260966* | 7.4620137 | 1.82E-13 |
| *RASD1* | 3.8622495 | 1.99E-12 |
| *LOC100524979* | 5.6763547 | 6.53E-12 |
| *RAPGEFL1* | 3.9873941 | 3.33E-11 |
| *BMP7* | 3.7133963 | 6.88E-11 |
| *RIMS1* | -3.9390997 | 1.21E-10 |
| *SGPP2* | 3.3637928 | 3.86E-10 |
| *RIPOR2* | 3.4902584 | 5.03E-10 |
| *LOC100737180* | 3.8198908 | 1.40E-09 |
| *PYGM* | -3.1619734 | 1.48E-09 |
| *RBFOX1* | 4.0085603 | 1.48E-09 |
| *NTRK3* | -3.3013241 | 2.38E-09 |
| *HPD* | -6.5425404 | 6.32E-09 |
| *LOXL2* | -4.1338575 | 6.89E-09 |
| *FZD10* | 3.5687705 | 1.24E-08 |
| *DPP6* | -3.3854049 | 1.26E-08 |
| *MYRF* | 3.0508168 | 1.69E-08 |
| *S100G* | 3.8079513 | 1.69E-08 |
| *FLRT3* | 5.2422781 | 3.64E-08 |
| *IGSF9B* | -3.0077151 | 4.30E-08 |
| *BBOX1* | 4.0012519 | 5.13E-08 |
| *PKHD1* | 3.1722439 | 5.13E-08 |
| *NGFR* | -4.037706 | 6.60E-08 |
| *IRS4* | 6.6181027 | 1.25E-07 |
| *TAC3* | 5.4367711 | 1.30E-07 |
| *TPM1* | -2.751833 | 1.30E-07 |
| *CHIA* | 3.0675846 | 2.85E-07 |
| *BCAS1* | 2.7296544 | 3.22E-07 |
| *BRINP2* | 3.6883706 | 3.38E-07 |
| *CRABP2* | 3.5305261 | 3.38E-07 |
| *RGL3* | 3.040158 | 3.92E-07 |
| *CACHD1* | 2.6777737 | 4.17E-07 |
| *GALNT6* | 2.7186647 | 4.37E-07 |
| *PRKAR2B* | 2.6957106 | 4.79E-07 |
| *COL24A1* | -3.2621518 | 8.37E-07 |
| *KCNT2* | 3.8417398 | 8.37E-07 |
| *ASTN2* | 3.0422254 | 8.48E-07 |
| *ZBED2* | 3.5116132 | 8.48E-07 |
| *LBP* | Inf | 1.18E-06 |
| *MPEG1* | 3.1726489 | 1.40E-06 |
| *SLC34A2* | 3.5638462 | 1.40E-06 |
| *SAMSN1* | 3.7420445 | 1.51E-06 |
| *LHCGR* | -5.1870769 | 1.52E-06 |
| *ADA2* | 2.7630973 | 2.56E-06 |
| *GMNN* | 2.545536 | 2.56E-06 |
| *LOC100738425* | 5.5683173 | 2.78E-06 |
| *TNFRSF19* | 2.724557 | 2.78E-06 |
| *FLNC* | -2.4704767 | 3.04E-06 |
| *LOC110255867* | 4.6629457 | 3.12E-06 |
| *SYNC* | -2.5190655 | 3.20E-06 |
| *CCR5* | 2.6715181 | 4.06E-06 |
| *DNAJB5* | -2.5767897 | 5.18E-06 |
| *KCNQ5* | -3.085065 | 5.91E-06 |
| *DMPK* | -2.5052656 | 8.25E-06 |
| *RASGRP1* | 2.9444504 | 8.48E-06 |
| *MYCN* | 4.901704 | 8.68E-06 |
| *GIMAP4* | 2.4477273 | 8.76E-06 |
| *UMOD* | 5.5237938 | 9.93E-06 |
| *CRABP1* | 2.5593709 | 1.01E-05 |
| *ITGA8* | -2.3749535 | 1.08E-05 |
| *PPP4R4* | 2.9909144 | 1.13E-05 |
| *SLC24A3* | -2.4866391 | 1.15E-05 |
| *LYZ* | 3.2623718 | 1.16E-05 |
| *CAMK2N1* | -2.4278616 | 1.30E-05 |
| *SOX7* | 2.6038504 | 1.51E-05 |
| *LGALS12* | 4.2397673 | 1.67E-05 |
| *HAS3* | 2.4649353 | 1.85E-05 |
| *CDCA7* | 4.5318446 | 1.85E-05 |
| *FBN3* | 5.1440867 | 2.06E-05 |
| *SSC5D* | -2.3424227 | 2.17E-05 |
| *PAK6* | 2.7608398 | 2.56E-05 |
| *ANXA9* | 2.8152167 | 2.59E-05 |
| *NTN4* | 2.3134434 | 2.74E-05 |
| *ACTA2* | -2.7977569 | 3.35E-05 |
| *BIN2* | 2.5062194 | 3.35E-05 |
| *ATP10A* | -2.4200159 | 3.64E-05 |
| *CA14* | -2.4955876 | 3.64E-05 |
| *SLC16A10* | 3.7246618 | 3.64E-05 |
| *RGMA* | -2.2383011 | 4.10E-05 |
| *CX3CR1* | 2.8430408 | 4.25E-05 |
| *PDLIM3* | -2.2089105 | 4.77E-05 |
| *GCNT3* | 2.4432007 | 4.79E-05 |
| *NLRP3* | 2.9084694 | 5.31E-05 |
| *XKR5* | 3.125825 | 5.33E-05 |
| *ANKRD55* | 4.7560333 | 5.74E-05 |
| *ANO4* | 2.3265881 | 5.74E-05 |
| *LOC100626715* | 3.1849759 | 5.74E-05 |
| *SYNM* | -2.168915 | 5.74E-05 |
| *SMTN* | -2.2537533 | 6.02E-05 |
| *P2RY12* | 2.6375836 | 6.06E-05 |
| *FHL2* | -2.2091711 | 6.30E-05 |
| *TERT* | 2.9168518 | 6.38E-05 |
| *RGS1* | 2.4039574 | 6.93E-05 |
| *ITGA5* | -2.2681365 | 7.63E-05 |
| *HPSE2* | -2.5490657 | 7.91E-05 |
| *FBXL22* | -2.3695295 | 8.31E-05 |
| *TRHDE* | -2.8570201 | 9.83E-05 |
| *LINGO3* | 4.3614316 | 0.0001032 |
| *CAP2* | -2.1436972 | 0.0001059 |
| *CXHXorf21* | 3.0213674 | 0.0001059 |
| *HABP2* | 2.5527952 | 0.0001128 |
| *IRF8* | 2.3018663 | 0.0001256 |
| *RASSF10* | 2.6940859 | 0.0001256 |
| *SQLE* | -2.2127055 | 0.0001256 |
| *LOC110260401* | -6.0949769 | 0.0001257 |
| *ERICH3* | 2.1909166 | 0.0001264 |
| *MYCL* | 2.7935712 | 0.0001264 |
| *PCDH15* | -2.5156216 | 0.0001319 |
| *ZBTB16* | -2.1571067 | 0.0001319 |
| *GIMAP1* | 2.1482886 | 0.0001369 |
| *NUAK2* | 2.3157171 | 0.0001369 |
| *PALM3* | 2.4454069 | 0.0001378 |
| *PDPN* | -2.2607475 | 0.0001378 |
| *PTN* | -2.2708798 | 0.0001378 |
| *RPS6KA6* | 2.3095072 | 0.0001378 |
| *LCN2* | 2.5014475 | 0.0001378 |
| *ASPG* | -5.7946601 | 0.0001424 |
| *SORCS1* | -2.1147114 | 0.0001424 |
| *WDFY4* | 2.4523401 | 0.0001451 |
| *PIK3CG* | 2.4074688 | 0.000151 |
| *KCNA3* | 3.0035375 | 0.0001605 |
| *PLK2* | 2.1051036 | 0.0001605 |
| *CKM* | -3.2102849 | 0.0001647 |
| *CNN1* | -2.1462127 | 0.0001647 |
| *GNG4* | -2.6354158 | 0.0001647 |
| *IL2RB* | 2.2737456 | 0.0001722 |
| *CFB* | 2.5863907 | 0.0001955 |
| *RASAL3* | 2.2976231 | 0.0001996 |
| *CITED4* | 2.7394426 | 0.0002055 |
| *LOC100737183* | 3.2687935 | 0.0002305 |
| *C13H3orf52* | 4.3542371 | 0.0002527 |
| *ALS2* | 2.0320693 | 0.0002582 |
| *GPR34* | 2.3570317 | 0.0002771 |
| *MAP3K21* | 2.0720953 | 0.00028 |
| *SLC7A8* | -2.4730275 | 0.0003237 |
| *ACTN2* | -3.013524 | 0.0003322 |
| *PKHD1L1* | 2.0645691 | 0.0003322 |
| *CD3G* | 2.6797956 | 0.0003401 |
| *SALL4* | 3.7391304 | 0.0003465 |
| *TPM2* | -2.3677917 | 0.0003465 |
| *SLC13A2* | 3.0349879 | 0.000352 |
| *ATP8B4* | 3.0492027 | 0.0003556 |
| *RNF182* | 6.1002652 | 0.0003595 |
| *LOC110259106* | 5.2794868 | 0.0003652 |
| *DLGAP1* | 2.0963976 | 0.0004217 |
| *MYLK* | -2.3476131 | 0.0004347 |
| *CCNJL* | 2.4893829 | 0.000448 |
| *FCGR1A* | 2.6371636 | 0.000448 |
| *LCP2* | 2.2282145 | 0.0004483 |
| *RAB6B* | -2.6616533 | 0.0004668 |
| *HAVCR1* | 5.2643955 | 0.0004778 |
| *MGAT4D* | 2.1956388 | 0.0004824 |
| *UBD* | 2.239516 | 0.0005139 |
| *IKZF3* | 2.5617807 | 0.000519 |
| *MYL9* | -2.6398636 | 0.000538 |
| *SLCO3A1* | -1.9593106 | 0.0005902 |
| *LOC100624785* | -2.0118232 | 0.000596 |
| *CLEC2L* | -6.7332971 | 0.0006001 |
| *CCL5* | 2.0548636 | 0.0006229 |
| *SKAP1* | 2.1118822 | 0.0006682 |
| *CKB* | -2.0730726 | 0.0006729 |
| *SEMA3A* | 2.9929498 | 0.0006975 |
| *PTPRC* | 2.4227895 | 0.0007298 |
| *AMBN* | 2.5825452 | 0.0007619 |
| *CD2* | 2.2188765 | 0.0007658 |
| *ACTG2* | -3.6059933 | 0.000768 |
| *LOC100624460* | 2.5521458 | 0.000768 |
| *LMOD1* | -1.8949184 | 0.0008032 |
| *CPVL* | 3.9417743 | 0.0008083 |
| *PPP1R3C* | -1.9434543 | 0.0008083 |
| *ITGAM* | 2.4177068 | 0.0008486 |
| *EFS* | -1.8951595 | 0.0008747 |
| *CCL8* | 3.3366479 | 0.0009214 |
| *NPNT* | 1.8733501 | 0.0009214 |
| *KLRK1* | 2.4308357 | 0.0009551 |
| *BANK1* | 2.198493 | 0.0009577 |
| *PPFIA4* | -2.483144 | 0.0009577 |
| *CADM1* | 1.8871647 | 0.0009747 |
| *VAV1* | 2.0015511 | 0.000975 |
| *P2RY13* | 2.6053757 | 0.0010085 |
| *CD83* | 2.1627724 | 0.0010129 |
| *MPZL2* | 2.0531345 | 0.0010224 |
| *NIPAL1* | 2.0361197 | 0.001086 |
| *TIFAB* | 3.2072831 | 0.0011173 |
| *JPH2* | -1.8539251 | 0.0011229 |
| *CD53* | 2.1650321 | 0.0011863 |
| *MKRN2OS* | 2.4245465 | 0.0011888 |
| *TNS1* | -1.8286953 | 0.0011965 |
| *ARHGAP1* | -1.8414688 | 0.0011968 |
| *CTNNAL1* | 1.869425 | 0.0011968 |
| *KYNU* | 2.6114864 | 0.0012197 |
| *ARNT2* | 1.8746356 | 0.0012213 |
| *CCNO* | Inf | 0.0012216 |
| *CRHR1* | -4.5291927 | 0.0012249 |
| *LAMB3* | 1.8927812 | 0.0012249 |
| *LEF1* | -1.8465353 | 0.0012249 |
| *TNFSF15* | 3.6346433 | 0.0012249 |
| *EMB* | 1.8997299 | 0.0012671 |
| *GPR20* | -2.1735071 | 0.0012753 |
| *CD48* | 2.3142504 | 0.001295 |
| *C9H1orf116* | 2.2082801 | 0.0013141 |
| *UPTI* | -9.3355229 | 0.0013172 |
| *JAK3* | 2.0571443 | 0.0013208 |
| *FCGR3A* | 1.8411771 | 0.0013466 |
| *AIF1* | 1.9448424 | 0.0013468 |
| *GPR31* | 2.8265864 | 0.0013468 |
| *LOC100520491* | 2.1712115 | 0.0013468 |
| *SELPLG* | 2.3659496 | 0.0013468 |
| *C3AR1* | 2.0851325 | 0.0013684 |
| *EVI2B* | 2.3195146 | 0.0014344 |
| *SEP1* | 2.2520873 | 0.0014768 |
| *PNCK* | -2.3393867 | 0.0014813 |
| *PDE8B* | 2.0987246 | 0.0014916 |
| *P4HA3* | -2.2106748 | 0.001525 |
| *FAM49A* | -1.925161 | 0.0015828 |
| *CASP1* | 2.3431886 | 0.0015888 |
| *GALNT3* | 2.3916876 | 0.0015888 |
| *LOC100521785* | 2.6775638 | 0.0015888 |
| *LTF* | 2.5148266 | 0.0015888 |
| *LOC100621844* | -3.4176068 | 0.0016047 |
| *INMT* | -2.389021 | 0.0016352 |
| *C5* | 3.3998455 | 0.0016383 |
| *HMGB2* | 1.8161253 | 0.0016466 |
| *DLL1* | -2.0727764 | 0.0016485 |
| *GYG1* | -2.1830133 | 0.0016485 |
| *NCKAP1L* | 1.94854 | 0.0016485 |
| *TAGLN* | -2.5750699 | 0.0016485 |
| *PLIN4* | -1.809278 | 0.0016659 |
| *SLC24A4* | -5.822783 | 0.0017147 |
| *DTNA* | -1.8682291 | 0.0017416 |
| *LOC110260197* | -1.8163585 | 0.0018075 |
| *CX3CL1* | 1.8077338 | 0.0018156 |
| *CFH* | 2.4089516 | 0.0018196 |
| *BIRC5* | 2.1345751 | 0.001843 |
| *DES* | -2.7860453 | 0.0018834 |
| *WISP2* | 1.7897044 | 0.0018834 |
| *DNAH5* | 3.2207578 | 0.0019035 |
| *TNNI1* | 3.117966 | 0.0019705 |
| *CCL26* | 2.0835595 | 0.0019768 |
| *FGD2* | 2.3798732 | 0.0020184 |
| *ADORA3* | 3.320639 | 0.0020213 |
| *INPP5D* | 1.9338955 | 0.0020213 |
| *SIDT1* | 1.9910494 | 0.0020296 |
| *ADRA2C* | -2.7603042 | 0.0021012 |
| *CORO1A* | 1.8357501 | 0.0021012 |
| *EHF* | 1.736819 | 0.002141 |
| *IL15* | 2.1432649 | 0.0021727 |
| *SPHK2* | -1.7671017 | 0.0021955 |
| *CD3D* | 3.1261659 | 0.0022005 |
| *CD84* | 2.4627404 | 0.0022101 |
| *SPINK13* | 2.8348121 | 0.0022768 |
| *PRSS23* | -1.7464794 | 0.0022891 |
| *RASSF5* | 2.0958009 | 0.0022891 |
| *CRTAC1* | -2.4327303 | 0.0023257 |
| *GLIS3* | 1.7781132 | 0.0023257 |
| *MCAM* | -1.7537274 | 0.0023328 |
| *NEXN* | -1.8900214 | 0.0023328 |
| *DUSP3* | -1.8032337 | 0.0023359 |
| *P2RX1* | -3.0903681 | 0.0023359 |
| *PRKG1* | -1.8057453 | 0.002353 |
| *GNAO1* | -1.8812588 | 0.0024336 |
| *TMEM59L* | -2.0532553 | 0.0024336 |
| *HS3ST2* | -2.6134009 | 0.0025085 |
| *LUZP2* | 2.7997902 | 0.0025085 |
| *ARHGAP4* | 1.8829172 | 0.0025555 |
| *FGFR4* | -3.5973764 | 0.0025555 |
| *ARHGAP30* | 2.242635 | 0.0025579 |
| *CCDC146* | 1.7929109 | 0.0026994 |
| *CXCR6* | 2.8656462 | 0.0026994 |
| *DCLK1* | -1.7494573 | 0.0026994 |
| *POPDC2* | -2.2561276 | 0.0026994 |
| *SLC1A4* | -2.1665086 | 0.0026994 |
| *TIAM1* | 2.1547636 | 0.0026994 |
| *CDH3* | -2.3910964 | 0.0027179 |
| *DCT* | 3.5039217 | 0.0027949 |
| *TMEM156* | 2.6725468 | 0.0028147 |
| *LOC100511343* | 2.061441 | 0.0028404 |
| *FSCN2* | -1.7978396 | 0.0028788 |
| *MICAL1* | -2.0192292 | 0.0028977 |
| *PCK2* | 1.8175008 | 0.0029624 |
| *SOWAHB* | 2.2526462 | 0.0030162 |
| *FERMT3* | 1.9008019 | 0.0031341 |
| *CNTFR* | 1.8246042 | 0.0032113 |
| *PRR5L* | 1.8502021 | 0.003249 |
| *LOC100622535* | 2.4598892 | 0.0032868 |
| *ID1* | 1.7118537 | 0.0032922 |
| *TRO* | 5.4205055 | 0.0033632 |
| *PDLIM7* | -2.1481495 | 0.0033738 |
| *PPP2R2B* | 4.0611369 | 0.0033738 |
| *PDE4C* | 1.8688226 | 0.0033861 |
| *ELF5* | 3.9204821 | 0.0034466 |
| *CNGA2* | 5.3560938 | 0.0035432 |
| *CCL4* | 3.3248133 | 0.0035525 |
| *P2RX2* | 4.0173724 | 0.0035966 |
| *SLC2A5* | 3.4707554 | 0.0036348 |
| *ZNF608* | 1.8597777 | 0.0036864 |
| *FAM3B* | 4.6328278 | 0.0038071 |
| *HGF* | 1.8730872 | 0.003816 |
| *BFSP1* | 3.3179697 | 0.0038418 |
| *AXIN2* | 1.6999389 | 0.0038872 |
| *TNFSF10* | 1.87363 | 0.0038872 |
| *LOC110257903* | 4.321049 | 0.0039107 |
| *C1QTNF6* | 1.9685998 | 0.0041004 |
| *IGF1* | -3.4112024 | 0.0041531 |
| *MSRB3* | -1.7956628 | 0.0041531 |
| *TNS4* | 2.0562618 | 0.0042493 |
| *FLRT1* | 4.0534203 | 0.0043117 |
| *TMEM74* | 2.238335 | 0.0043191 |
| *KCNH2* | -2.0238614 | 0.0043384 |
| *SAMD11* | 1.8699703 | 0.0043442 |
| *LOC100739325* | 2.1015394 | 0.004366 |
| *LOC100520216* | 1.8789462 | 0.0043811 |
| *LOC100622849* | 1.8320803 | 0.0044286 |
| *TGFB1I1* | -1.6656775 | 0.0044337 |
| *ERICH2* | 2.2457228 | 0.0044678 |
| *LPXN* | 2.1013699 | 0.0045262 |
| *MS4A7* | 2.1215122 | 0.0045402 |
| *KLHL6* | 2.4110777 | 0.0045751 |
| *TMEM221* | 3.0989987 | 0.0045996 |
| *LOC100525483* | -6.7782398 | 0.0046635 |
| *CACNG4* | 3.0251764 | 0.0046844 |
| *HES1* | 1.7242045 | 0.0046844 |
| *NR3C2* | 1.814564 | 0.0046912 |
| *PCP4* | -2.724195 | 0.0047844 |
| *SORBS1* | -1.7683878 | 0.0048191 |
| *TMEM47* | -1.6368722 | 0.004833 |
| *ANKS4B* | 1.660059 | 0.0050826 |
| *CD151* | -1.6434563 | 0.0051789 |
| *CTSS* | 1.6366182 | 0.0052146 |
| *RAC2* | 2.0797304 | 0.0053348 |
| *C1QC* | 1.9068453 | 0.0053536 |
| *SASH3* | 1.7994203 | 0.0054244 |
| *CACNA1H* | -1.674382 | 0.0054338 |
| *TYROBP* | 1.8152217 | 0.0055235 |
| *UPK3A* | 1.9422868 | 0.0055235 |
| *ALOX5* | 2.1379534 | 0.0056592 |
| *LOC110260191* | 2.6456342 | 0.0057149 |
| *CAV1* | -1.6153281 | 0.0060964 |
| *PRELP* | -1.6727808 | 0.0060964 |
| *PLXNB3* | 2.4143538 | 0.0062742 |
| *CD52* | 1.9233704 | 0.0062966 |
| *NAALADL1* | 2.0255562 | 0.0063582 |
| *SEMA6D* | 1.7268852 | 0.0063582 |
| *IKZF1* | 1.9817127 | 0.006392 |
| *ROBO1* | 2.3102168 | 0.0064865 |
| *HCK* | 1.983306 | 0.0065241 |
| *LOC100625049* | -1.6473429 | 0.006736 |
| *CD55* | -1.898821 | 0.0068209 |
| *STAMBPL1* | 2.3028372 | 0.0068564 |
| *GRIN2C* | Inf | 0.0068953 |
| *GPRASP2* | 1.6589364 | 0.0068974 |
| *PITPNC1* | -2.865424 | 0.0070757 |
| *BTK* | 2.1190465 | 0.0072019 |
| *MCIDAS* | Inf | 0.0072019 |
| *RGS10* | 1.7715844 | 0.0072019 |
| *SLC25A25* | -1.7944579 | 0.0072191 |
| *CDC20B* | Inf | 0.0073444 |
| *MGAM2* | -1.721145 | 0.0074394 |
| *OIP5* | 2.1915997 | 0.0074555 |
| *ACTN1* | -1.5746838 | 0.0077604 |
| *ASGR2* | 2.93652 | 0.0078968 |
| *CMTM4* | -1.5771675 | 0.0081348 |
| *HCLS1* | 1.6808254 | 0.0081348 |
| *LRRC25* | 2.3374985 | 0.0081348 |
| *PIK3AP1* | 1.6551003 | 0.0082328 |
| *ADAMTS17* | -1.7201096 | 0.0083288 |
| *C1QB* | 1.6121049 | 0.0083288 |
| *RPH3A* | 3.9267193 | 0.0083288 |
| *NABP1* | 1.7047161 | 0.0084095 |
| *APBA1* | -1.7067332 | 0.00863 |
| *SLC7A4* | 1.9972162 | 0.00863 |
| *SPON1* | -3.6954882 | 0.0086435 |
| *NUP62CL* | 2.446386 | 0.0086803 |
| *HNF4G* | 1.8192572 | 0.0087673 |
| *LOC100520329* | -1.5684068 | 0.0087848 |
| *MCOLN2* | 2.4345708 | 0.0089041 |
| *WSCD2* | -1.8091313 | 0.0089207 |
| *LAPTM5* | 1.5461637 | 0.0089737 |
| *MEST* | 2.5529313 | 0.0090912 |
| *TFRC* | -1.6627919 | 0.0091371 |
| *ACTA1* | -2.1649208 | 0.0091428 |
| *LOC100158069* | 2.9384939 | 0.0091428 |
| *VCL* | -1.5504537 | 0.0091428 |
| *ALPK1* | 1.7185547 | 0.0094673 |
| *FER1L5* | 2.8530607 | 0.0094673 |
| *SLC15A2* | 1.5702864 | 0.0094673 |
| *MCOLN3* | -2.8622089 | 0.0096563 |
| *NECAB3* | 1.5969473 | 0.0097924 |
| *KCNB1* | -1.6673561 | 0.0098742 |
| *FAM46B* | -2.9572426 | 0.0100484 |
| *GLDC* | 2.0277207 | 0.0100484 |
| *TESMIN* | 3.1740081 | 0.0100484 |
| *LOC110259229* | -1.7425935 | 0.0102325 |
| *TP53INP2* | -2.1862767 | 0.0102325 |
| *DYSF* | -1.5874107 | 0.010387 |
| *BAG2* | -1.7647511 | 0.010535 |
| *C1H14orf37* | 1.5722409 | 0.0105575 |
| *NFIL3* | -1.5513644 | 0.0105575 |
| *PTGER1* | -1.9130632 | 0.0105575 |
| *PCDH9* | 2.5699464 | 0.0106906 |
| *CD8A* | 3.1500028 | 0.0108706 |
| *EMID1* | 2.1881628 | 0.0108706 |
| *LOC100626505* | 2.2087136 | 0.0108706 |
| *CDKL4* | 1.7457916 | 0.0109898 |
| *MICALL1* | -1.5537134 | 0.0110028 |
| *TREM2* | 2.7531088 | 0.0111096 |
| *SIX4* | 2.0013289 | 0.0111258 |
| *PLCG2* | 1.6701064 | 0.0115433 |
| *PARP8* | 1.6119 | 0.0115707 |
| *RASSF3* | -1.5079591 | 0.0115762 |
| *ATOH8* | 2.0996555 | 0.0117363 |
| *CORO2A* | 1.7903597 | 0.0117363 |
| *FAM131B* | -2.0567503 | 0.0117363 |
| *GEN1* | 2.9600555 | 0.0120733 |
| *LOC100523789* | 2.1860531 | 0.012121 |
| *XCL1* | 2.5169521 | 0.0121604 |
| *PDZK1IP1* | 2.823001 | 0.0121735 |
| *ACSL5* | 1.5306837 | 0.0122835 |
| *MYOZ1* | 3.2487212 | 0.0124091 |
| *CFL2* | -1.5168701 | 0.0126488 |
| *MAL* | -1.9515687 | 0.0126488 |
| *PSMB9* | 1.6188806 | 0.0126488 |
| *FYB* | 2.1453532 | 0.0126774 |
| *KCNMB1* | -1.5107465 | 0.0126953 |
| *TNFAIP8L2* | 1.9181561 | 0.0128972 |
| *CD80* | 2.9357387 | 0.0131251 |
| *LCK* | 2.0722303 | 0.013241 |
| *PTGER4* | 1.5848758 | 0.0132465 |
| *BAIAP2L1* | 1.5379349 | 0.0132963 |
| *SLAMF6* | 2.622896 | 0.0133245 |
| *BHLHB9* | 1.5598484 | 0.0134612 |
| *PDLIM4* | -1.5260734 | 0.0134749 |
| *DNAJC22* | 2.1754889 | 0.013515 |
| *PROX1* | -2.0784765 | 0.013515 |
| *PHACTR1* | 1.6243085 | 0.0135606 |
| *RASL12* | -1.6824277 | 0.0135609 |
| *SLA-DMB* | 1.5605296 | 0.0139263 |
| *IRF5* | 1.6816869 | 0.0140169 |
| *KCNE5* | 3.6797865 | 0.0143645 |
| *C7H6orf223* | 3.359942 | 0.01441 |
| *EFHC2* | 1.9360236 | 0.01441 |
| *FIBIN* | 1.5233779 | 0.0145502 |
| *SLITRK6* | 2.5473643 | 0.0145661 |
| *MYO1F* | 1.8020934 | 0.0146245 |
| *CD27* | 2.6679969 | 0.0146653 |
| *LY75* | 3.684811 | 0.0150348 |
| *HS3ST5* | -1.7413811 | 0.0151453 |
| *GNA15* | 1.7528263 | 0.0151853 |
| *TLR10* | 2.5915845 | 0.0152635 |
| *TLR2* | 1.7155325 | 0.0153687 |
| *THEMIS* | 3.2808544 | 0.0154515 |
| *CXCL11* | 4.6332253 | 0.015723 |
| *PRF1* | 2.1260447 | 0.0157486 |
| *FLNA* | -2.0223577 | 0.0157929 |
| *LOC102165484* | 2.4485531 | 0.0158232 |
| *GPM6A* | 1.5531411 | 0.0158557 |
| *STX3* | -1.5163085 | 0.0158557 |
| *CTSO* | 1.6528368 | 0.0158818 |
| *ID3* | 1.4824456 | 0.0158818 |
| *LIMS2* | -1.9572817 | 0.0158818 |
| *MAOB* | -1.4940033 | 0.0158818 |
| *FOXN4* | 7.0213372 | 0.016189 |
| *KLHL23* | -1.4872934 | 0.016189 |
| *BOK* | -1.4979725 | 0.0163898 |
| *TMEM213* | 3.5699081 | 0.0165576 |
| *PREX1* | 1.5318416 | 0.0165843 |
| *FNBP1* | -1.4602366 | 0.0166278 |
| *ZNF510* | 1.9222423 | 0.0166835 |
| *DHRS9* | 3.065994 | 0.0167014 |
| *LY6E* | 1.5071102 | 0.0167837 |
| *LOC110255332* | -3.7743089 | 0.016816 |
| *SH3BP4* | -1.5165413 | 0.0168618 |
| *HEY2* | 2.2352888 | 0.017049 |
| *FAM19A2* | -2.7294987 | 0.0170755 |
| *EVI2A* | 1.9932982 | 0.0171741 |
| *MAN1C1* | 1.4564205 | 0.0174004 |
| *ZNF683* | 2.3790174 | 0.0174004 |
| *AMOTL1* | -2.6987198 | 0.0176911 |
| *VSTM2A* | -5.1923387 | 0.0176976 |
| *LOC102158035* | 2.3193065 | 0.0180377 |
| *KLF11* | 1.7436459 | 0.0181434 |
| *RBPMS2* | -1.5589448 | 0.0181434 |
| *SSX2IP* | 1.5195538 | 0.0181688 |
| *ITK* | 2.3675617 | 0.0181717 |
| *CBR1* | -4.7683597 | 0.0181963 |
| *LOC110256000* | -3.6994521 | 0.0181963 |
| *OPRL1* | 1.894077 | 0.018367 |
| *GFI1* | 2.8583012 | 0.0183712 |
| *PCP4L1* | -1.8104067 | 0.0183843 |
| *TMC8* | 1.9019089 | 0.0186277 |
| *ANXA6* | -1.4628164 | 0.0187214 |
| *MYH11* | -1.6702016 | 0.0188307 |
| *CD300A* | 2.2412622 | 0.0189089 |
| *ARSE* | 1.5541223 | 0.0189847 |
| *PTAFR* | 1.7815063 | 0.0189898 |
| *NACC2* | -1.4703754 | 0.0190353 |
| *CD8B* | 3.2733145 | 0.0190584 |
| *SREBF1* | -1.4530722 | 0.0190636 |
| *GULP1* | 1.5988886 | 0.0191576 |
| *PLXNA4* | -2.8603282 | 0.0191697 |
| *SEZ6L* | -3.5627074 | 0.0191816 |
| *NEK6* | 1.4780194 | 0.0192144 |
| *FABP6* | -4.0362341 | 0.0194916 |
| *SIT1* | 2.0417743 | 0.0194916 |
| *SLC15A1* | 3.448531 | 0.0196178 |
| *DOCK8* | 1.5954696 | 0.0196848 |
| *HNF1A* | 3.66162 | 0.0198311 |
| *ME3* | -1.4755369 | 0.019927 |
| *PLA2G5* | 6.0982722 | 0.019927 |
| *PLCB2* | 1.7399079 | 0.019927 |
| *DAPP1* | 1.7013416 | 0.0203671 |
| *GSAP* | 1.5265841 | 0.0203671 |
| *PKDCC* | -1.4675202 | 0.0203671 |
| *TSPAN18* | -1.4573904 | 0.0203671 |
| *RASSF9* | 1.5650779 | 0.0203915 |
| *GPR85* | 1.7632248 | 0.0206723 |
| *ANKEF1* | 2.064557 | 0.0209189 |
| *LCA5* | 1.6686714 | 0.0209189 |
| *SPI1* | 1.508464 | 0.0209639 |
| *LRRIQ1* | 1.7296712 | 0.0213585 |
| *MATN1* | 3.3997434 | 0.0213829 |
| *PAH* | -3.5306441 | 0.0213829 |
| *KIAA1324L* | -2.2174309 | 0.0217142 |
| *HACD1* | -1.5205913 | 0.0217734 |
| *VASP* | -1.435032 | 0.0218043 |
| *DMBT1* | 4.706239 | 0.0220204 |
| *PCDH20* | 2.2412615 | 0.0220204 |
| *CIITA* | 1.5180278 | 0.0223404 |
| *BCAR1* | -1.4118571 | 0.0229012 |
| *CAVIN1* | -1.4140691 | 0.0230901 |
| *SLITRK4* | 3.8128355 | 0.0230901 |
| *UABP-2* | -10.55369 | 0.0233446 |
| *CYTH4* | 1.9287662 | 0.023418 |
| *NGEF* | 1.4695821 | 0.0235455 |
| *MX2* | 1.844414 | 0.0237624 |
| *EFCAB8* | -2.3130892 | 0.0239215 |
| *STPG1* | 1.6161403 | 0.0241031 |
| *STAP1* | 2.2028788 | 0.0242239 |
| *LDLR* | -1.8166213 | 0.0244367 |
| *HIPK2* | -1.9487482 | 0.0246214 |
| *AK4* | -1.4799859 | 0.0247252 |
| *MICAL2* | -2.26329 | 0.0248501 |
| *LOC100512793* | -2.1807182 | 0.02497 |
| *CSPG5* | -2.2191162 | 0.0250861 |
| *EMR4* | 1.8232864 | 0.0250861 |
| *FAM43B* | 2.082726 | 0.0250861 |
| *PDE6C* | -5.8914275 | 0.0250861 |
| *SLA-DRA* | 1.8764078 | 0.0250861 |
| *KCNMA1* | -1.3960362 | 0.0252464 |
| *MAP3K19* | 1.7970137 | 0.0252529 |
| *PPFIA2* | -2.1779033 | 0.0252529 |
| *LOC100155195* | 2.0627233 | 0.0253032 |
| *SRRM4* | 3.14994 | 0.0253547 |
| *COL9A2* | 3.7076348 | 0.0254298 |
| *LOXL1* | -1.5407136 | 0.0257109 |
| *SNAPC2* | -1.4788124 | 0.0257109 |
| *SEZ6* | 2.63855 | 0.0257738 |
| *ST6GALNAC4* | -1.4096958 | 0.0257738 |
| *MSX2* | 1.6704691 | 0.0258378 |
| *TTC29* | 1.8425062 | 0.0258378 |
| *RUNX3* | 2.3391525 | 0.0259053 |
| *CSRP1* | -1.7410118 | 0.0259219 |
| *DEUP1* | 2.4921353 | 0.0259219 |
| *IL17B* | -1.7729372 | 0.0259219 |
| *PTPN22* | 2.6819384 | 0.0259347 |
| *ERAP2* | 1.5053076 | 0.0260752 |
| *FSTL3* | -1.4785335 | 0.0260752 |
| *SCG3* | -2.5733558 | 0.0260752 |
| *SHANK2* | 1.4342109 | 0.0262854 |
| *MAP3K1* | 1.4174437 | 0.0263129 |
| *NKG7* | 1.6660278 | 0.0263864 |
| *PLA2G2D* | 3.5939987 | 0.0264231 |
| *LIN7A* | 2.1029206 | 0.0264325 |
| *C1QTNF2* | -1.5161123 | 0.0268163 |
| *LOC100154053* | 2.463146 | 0.0268163 |
| *MYL6* | -1.3868731 | 0.0269574 |
| *MAP2* | 1.5733046 | 0.0269785 |
| *ENOX1* | -1.5466431 | 0.0269799 |
| *NEK2* | 4.3015403 | 0.0269862 |
| *MYO1C* | -1.3924296 | 0.0270331 |
| *ITGB1BP2* | -2.2927564 | 0.0270385 |
| *FLRT2* | 1.4315851 | 0.0270402 |
| *SPATA17* | 1.7177595 | 0.0270402 |
| *SFXN3* | -1.5531519 | 0.0270909 |
| *ACAP1* | 1.6699804 | 0.0270913 |
| *CACNA1B* | -6.1143739 | 0.027217 |
| *CCDC148* | 2.4780109 | 0.0273794 |
| *NUP210* | 1.4991112 | 0.0273794 |
| *AK7* | 1.8254593 | 0.0274679 |
| *RARB* | 1.6526404 | 0.0276512 |
| *MAMLD1* | -1.6188807 | 0.0280651 |
| *MILR1* | 2.4349133 | 0.0280651 |
| *CD36* | 1.6551354 | 0.0281121 |
| *LOC100626407* | 1.5465118 | 0.0282058 |
| *GP91-PHOX* | 1.9485817 | 0.0283324 |
| *LOC100738859* | -1.414156 | 0.0283324 |
| *CNTNAP1* | -1.6251593 | 0.0287504 |
| *SLC9A7* | 1.5562914 | 0.0287942 |
| *COL1A1* | -1.7595647 | 0.0288816 |
| *IPCEF1* | 2.2416522 | 0.0288816 |
| *PSMB8* | 1.3837388 | 0.0288816 |
| *ITGA7* | -1.6537354 | 0.0288978 |
| *SESN2* | -1.5135987 | 0.0289775 |
| *DOCK2* | 1.8306781 | 0.0291557 |
| *E2F7* | 4.9039723 | 0.0291557 |
| *ITGA4* | 2.8159846 | 0.029347 |
| *COL6A3* | -3.1290183 | 0.0293619 |
| *MSRB1* | -1.3884207 | 0.0295149 |
| *CCDC88B* | 1.7663321 | 0.0299569 |
| *PFN1* | -1.3662928 | 0.0299569 |
| *LOC100520992* | 1.636656 | 0.0299974 |
| *LOC100736962* | 3.3438526 | 0.0299974 |
| *SCN1B* | -1.8773273 | 0.0300137 |
| *KCNIP2* | -2.4961203 | 0.0300353 |
| *SH3BP1* | 1.5305944 | 0.0300762 |
| *CAMK4* | 1.8418565 | 0.0301988 |
| *LGALS1* | -1.390035 | 0.0302785 |
| *DACH2* | 4.266833 | 0.0304165 |
| *PTGES3L* | -1.4502597 | 0.0304964 |
| *ACTB* | -1.3544981 | 0.030772 |
| *SPEG* | -1.7552553 | 0.0309554 |
| *CD79B* | 2.0434923 | 0.0310879 |
| *TMEM61* | -1.952186 | 0.031154 |
| *ASB2* | -1.5049011 | 0.0311687 |
| *GPR183* | 2.3032431 | 0.0314175 |
| *LOC100513119* | Inf | 0.0314175 |
| *NDST3* | 3.1014427 | 0.0314175 |
| *TXNIP* | 1.3436621 | 0.0314332 |
| *PPP1R1A* | -1.9713744 | 0.0317612 |
| *MIS18A* | 1.9922743 | 0.0319107 |
| *SLITRK5* | -2.1526508 | 0.0319107 |
| *STBD1* | -1.3758227 | 0.0319107 |
| *NEGR1* | -1.6147815 | 0.0320741 |
| *SCUBE1* | -2.6698795 | 0.0321618 |
| *PHACTR3* | -2.45804 | 0.0325932 |
| *LCP1* | 1.3661069 | 0.0326358 |
| *PLAC8* | 1.5119415 | 0.0326358 |
| *RASAL1* | 1.7430324 | 0.0326358 |
| *CAMKK1* | -1.5345515 | 0.0328025 |
| *ADAMTSL3* | -1.3927865 | 0.0328159 |
| *ARVCF* | -1.368143 | 0.032856 |
| *FAM212B* | -1.5552689 | 0.0330226 |
| *ARHGAP15* | 1.990168 | 0.033034 |
| *B4GALT1* | -1.3553611 | 0.033034 |
| *GRIN2A* | -1.6962316 | 0.033034 |
| *LOC100525573* | Inf | 0.033034 |
| *RAB3B* | -1.6900185 | 0.033034 |
| *BDH1* | -1.541533 | 0.0330984 |
| *ZNF385B* | 1.8798213 | 0.0332314 |
| *CCNE2* | 2.0163844 | 0.0335378 |
| *ICAM3* | 1.920973 | 0.0335378 |
| *PARVG* | 2.1868225 | 0.0335378 |
| *SLC46A1* | 1.4126423 | 0.0335695 |
| *IRS2* | 1.3729487 | 0.0335987 |
| *B4GALNT3* | -1.4491151 | 0.0337636 |
| *XCR1* | 3.0404347 | 0.0339107 |
| *RIMS3* | -1.8968056 | 0.0342337 |
| *CSF2RB* | 2.1504934 | 0.0344808 |
| *CDCA3* | 2.3687067 | 0.0345031 |
| *RACGAP1* | 2.5239649 | 0.0345031 |
| *KIF1A* | -2.8790792 | 0.035246 |
| *CXCL9* | 4.2651958 | 0.0357202 |
| *PPP1R12C* | -1.3352891 | 0.0357591 |
| *PLD4* | 2.0740013 | 0.0357946 |
| *CCDC173* | 1.622532 | 0.0358104 |
| *NDRG4* | -3.4574099 | 0.0358193 |
| *RASD2* | -1.7039729 | 0.0363018 |
| *WIPI1* | -1.535918 | 0.0364577 |
| *WNK4* | -1.4491658 | 0.0364577 |
| *CDH12* | -3.8721894 | 0.0367118 |
| *ATP2B3* | -2.5593569 | 0.0370266 |
| *C3H16orf54* | 1.5158297 | 0.0372201 |
| *ENO2* | 1.4036784 | 0.0378752 |
| *LOC102161784* | 2.1656924 | 0.0378947 |
| *TLN1* | -1.3206861 | 0.0379328 |
| *MEOX1* | -2.390284 | 0.0380039 |
| *HSPG2* | -1.3283034 | 0.0383367 |
| *RIMKLA* | -2.2262722 | 0.0383367 |
| *CSF1R* | 1.3466653 | 0.0385339 |
| *FCER1G* | 1.7597835 | 0.0385339 |
| *NFE2L3* | 1.8130641 | 0.0385339 |
| *UBE2QL1* | 1.9810912 | 0.0386578 |
| *ADIRF* | 1.3483107 | 0.0389631 |
| *PLIN5* | 2.6505974 | 0.0392363 |
| *SPAG17* | 2.1655529 | 0.0392465 |
| *RASL11A* | -1.4242418 | 0.039622 |
| *RGS2* | -2.6626607 | 0.039622 |
| *UBE2U* | 2.2544973 | 0.0398187 |
| *TRIOBP* | -1.3181696 | 0.0398959 |
| *ST5* | -1.3326388 | 0.0400349 |
| *C4H8orf46* | -1.8589659 | 0.0401316 |
| *GPRASP1* | 1.4524751 | 0.0405951 |
| *ITGB7* | 1.880463 | 0.0406432 |
| *TNFAIP2* | 1.3407612 | 0.040787 |
| *SH3BGR* | -1.3518892 | 0.0408489 |
| *STARD13* | -1.3526393 | 0.0408906 |
| *DUSP8* | -1.5353271 | 0.0409883 |
| *TTLL7* | -1.4442881 | 0.0409883 |
| *ZBTB12* | 1.4068054 | 0.0409883 |
| *NKX6-1* | 4.8026936 | 0.0410839 |
| *SMARCD3* | -2.0155132 | 0.0412384 |
| *LOC110257072* | 2.5658192 | 0.0414639 |
| *MGAT5* | -1.3478379 | 0.0417299 |
| *CAPN6* | 1.9103376 | 0.0418419 |
| *CD244* | 2.0645894 | 0.0425587 |
| *CDC20* | 3.6666839 | 0.0425587 |
| *ARHGAP45* | 1.3396006 | 0.0426533 |
| *LOC106507765* | 2.3199203 | 0.0426533 |
| *CSPG4* | -2.262727 | 0.0428421 |
| *ADGB* | 1.7820142 | 0.0430034 |
| *ARHGAP26* | 1.4268943 | 0.0430034 |
| *GAS2L2* | 2.1305893 | 0.0430034 |
| *GPR65* | 2.3716502 | 0.0431127 |
| *EPHA3* | 2.0663722 | 0.0434362 |
| *NPPB* | #NAME? | 0.0434362 |
| *IL16* | 1.6143628 | 0.0435043 |
| *PLA2G7* | -1.3032515 | 0.0435269 |
| *TFEC* | 1.9202779 | 0.0435981 |
| *LOC100739719* | 1.7915799 | 0.0436274 |
| *SLC13A4* | 4.7332314 | 0.0436274 |
| *SDC3* | -1.327077 | 0.0436628 |
| *KBTBD11* | 1.3669085 | 0.0436794 |
| *AFAP1* | -1.3841932 | 0.0442976 |
| *ARHGAP20* | 1.5235971 | 0.0445359 |
| *SUSD5* | 1.981976 | 0.0448717 |
| *GZMA* | 4.1099327 | 0.0450412 |
| *PROSER2* | 1.5219142 | 0.0451091 |
| *FANCD2* | 1.4870425 | 0.0451781 |
| *CALD1* | -1.2804851 | 0.0453976 |
| *ENPP6* | 1.6241887 | 0.0454937 |
| *PTPN7* | 1.6837726 | 0.0456207 |
| *APOBR* | 1.560656 | 0.0457474 |
| *ARL11* | 1.7618186 | 0.0460182 |
| *ERICH6* | 4.1584592 | 0.0460182 |
| *RTN4RL1* | -1.4759344 | 0.0460182 |
| *STIL* | 1.963425 | 0.0460182 |
| *KLHL34* | 2.3416332 | 0.0461595 |
| *WNT7A* | 2.4473326 | 0.0461595 |
| *SLC23A1* | -5.7499394 | 0.0463658 |
| *B2M* | 1.2752102 | 0.0466935 |
| *CRHR2* | -2.1372657 | 0.0466935 |
| *JAM3* | -1.3207655 | 0.0466935 |
| *KIF22* | 1.8056575 | 0.0466935 |
| *SRGN* | 1.355006 | 0.0466935 |
| *CORO1C* | -1.2936336 | 0.0470444 |
| *ABCB11* | 3.7095495 | 0.0474295 |
| *VWDE* | 4.8373463 | 0.047589 |
| *SRF* | -1.2893662 | 0.0479394 |
| *FAIM2* | 2.1084324 | 0.048018 |
| *TBX1* | -1.7576299 | 0.048018 |
| *DOK2* | 1.4637354 | 0.048069 |
| *KIF2C* | 2.6987671 | 0.048069 |
| *PLPPR3* | 1.4897011 | 0.048069 |
| *GMFG* | 1.4835537 | 0.0481284 |
| *CD96* | 2.041003 | 0.0481959 |
| *IL2RG* | 1.5491518 | 0.0481959 |
| *PHF21B* | 2.4531256 | 0.0481959 |
| *TMEM63C* | 1.9240795 | 0.0482643 |
| *ADCY5* | -1.3664156 | 0.0482679 |
| *PIGZ* | 2.6739024 | 0.0482679 |
| *NRXN3* | 3.5950868 | 0.0484912 |
| *CYP2B22* | -1.6758146 | 0.0485257 |
| *MAP6D1* | -2.066039 | 0.0485257 |
| *LOC110261963* | 2.3275782 | 0.0485942 |
| *PLEKHG6* | 1.5092813 | 0.0486669 |
| *MGLL* | -1.2813615 | 0.0486794 |
| *CYP2A19* | 1.6792168 | 0.0488889 |
| *GLIS1* | -1.7552606 | 0.0489084 |
| *TLR9* | 2.3176644 | 0.0489869 |
| *RASGRP2* | -1.4149638 | 0.0491577 |
| *TVP23A* | 3.6386043 | 0.0491577 |
| *LRRC36* | 1.7110055 | 0.049202 |
| *NGB* | 3.3581711 | 0.049202 |
| *CDK1* | 3.6522246 | 0.0496739 |
| *KCND3* | -2.2081095 | 0.0496739 |
| *AGAP1* | -1.9487044 | 0.0498346 |

**Table S3. GO enrichment of highly expressed genes in the PU compared to genes in the SU.**

| **id** | **term** | **ListHits** | **padj** |
| --- | --- | --- | --- |
| GO:1903251 | multi-ciliated epithelial cell differentiation | 4 | 0 |
| GO:0009897 | external side of plasma membrane | 51 | 2.05E-16 |
| GO:0006955 | immune response | 41 | 1.72E-14 |
| GO:0007018 | microtubule-based movement | 23 | 6.32E-12 |
| GO:0003777 | microtubule motor activity | 21 | 3.91E-11 |
| GO:0006935 | chemotaxis | 22 | 3.63E-10 |
| GO:0007059 | chromosome segregation | 17 | 5.49E-09 |
| GO:0002376 | immune system process | 16 | 1.37E-08 |
| GO:0042102 | positive regulation of T cell proliferation | 16 | 2.09E-08 |
| GO:0070098 | chemokine-mediated signaling pathway | 16 | 3.13E-08 |
| GO:0000775 | chromosome, centromeric region | 13 | 4.88E-08 |
| GO:0003774 | motor activity | 20 | 7.61E-08 |
| GO:0008017 | microtubule binding | 33 | 1.49E-07 |
| GO:0005871 | kinesin complex | 14 | 1.98E-07 |
| GO:0000278 | mitotic cell cycle | 21 | 3.10E-07 |
| GO:0006954 | inflammatory response | 28 | 5.24E-07 |
| GO:0045954 | positive regulation of natural killer cell mediated cytotoxicity | 8 | 1.23E-06 |
| GO:0050853 | B cell receptor signaling pathway | 10 | 1.83E-06 |
| GO:0030890 | positive regulation of B cell proliferation | 11 | 7.15E-06 |
| GO:0030217 | T cell differentiation | 11 | 7.15E-06 |
| GO:0045059 | positive thymic T cell selection | 6 | 7.41E-06 |
| GO:0009986 | cell surface | 50 | 8.46E-06 |
| GO:2000353 | positive regulation of endothelial cell apoptotic process | 7 | 1.05E-05 |
| GO:0005874 | microtubule | 25 | 1.26E-05 |
| GO:0005089 | Rho guanyl-nucleotide exchange factor activity | 16 | 1.36E-05 |
| GO:0042613 | MHC class II protein complex | 4 | 1.53E-05 |
| GO:0050869 | negative regulation of B cell activation | 4 | 1.53E-05 |
| GO:0007080 | mitotic metaphase plate congression | 10 | 2.65E-05 |
| GO:0003341 | cilium movement | 8 | 2.91E-05 |
| GO:0000281 | mitotic cytokinesis | 13 | 3.64E-05 |
| GO:0032722 | positive regulation of chemokine production | 7 | 3.91E-05 |
| GO:0060271 | cilium assembly | 25 | 5.06E-05 |
| GO:0030593 | neutrophil chemotaxis | 11 | 5.64E-05 |
| GO:0004950 | chemokine receptor activity | 7 | 6.87E-05 |
| GO:0019956 | chemokine binding | 7 | 6.87E-05 |
| GO:0042101 | T cell receptor complex | 5 | 7.26E-05 |
| GO:0008569 | ATP-dependent microtubule motor activity, minus-end-directed | 5 | 7.26E-05 |
| GO:0035023 | regulation of Rho protein signal transduction | 16 | 7.34E-05 |
| GO:0043547 | positive regulation of GTPase activity | 33 | 7.60E-05 |
| GO:0010818 | T cell chemotaxis | 4 | 8.37E-05 |
| GO:0001771 | immunological synapse formation | 4 | 8.37E-05 |
| GO:0043303 | mast cell degranulation | 4 | 8.37E-05 |
| GO:0050868 | negative regulation of T cell activation | 6 | 8.38E-05 |
| GO:0005819 | spindle | 12 | 8.92E-05 |
| GO:0001772 | immunological synapse | 9 | 9.59E-05 |
| GO:0030286 | dynein complex | 8 | 0.0001126 |
| GO:0007166 | cell surface receptor signaling pathway | 21 | 0.0001275 |
| GO:0060326 | cell chemotaxis | 13 | 0.0001524 |
| GO:0006911 | phagocytosis, engulfment | 6 | 0.0001551 |
| GO:0032733 | positive regulation of interleukin-10 production | 6 | 0.0001551 |
| GO:0005929 | cilium | 22 | 0.000156 |
| GO:0030676 | Rac guanyl-nucleotide exchange factor activity | 5 | 0.0001649 |
| GO:0008574 | ATP-dependent microtubule motor activity, plus-end-directed | 5 | 0.0001649 |
| GO:0007052 | mitotic spindle organization | 8 | 0.0001663 |
| GO:0007088 | regulation of mitotic nuclear division | 7 | 0.0001805 |
| GO:0071222 | cellular response to lipopolysaccharide | 17 | 0.0001864 |
| GO:0009617 | response to bacterium | 17 | 0.000254 |
| GO:0042629 | mast cell granule | 4 | 0.0002618 |
| GO:0016176 | superoxide-generating NADPH oxidase activator activity | 4 | 0.0002618 |
| GO:0016493 | C-C chemokine receptor activity | 7 | 0.000275 |
| GO:0007165 | signal transduction | 76 | 0.0002771 |
| GO:0051310 | metaphase plate congression | 5 | 0.0003288 |
| GO:0002755 | MyD88-dependent toll-like receptor signaling pathway | 5 | 0.0003288 |
| GO:0000922 | spindle pole | 15 | 0.0003365 |
| GO:0008009 | chemokine activity | 8 | 0.0004736 |
| GO:0035556 | intracellular signal transduction | 40 | 0.0005666 |
| GO:0045787 | positive regulation of cell cycle | 9 | 0.0006113 |
| GO:0050870 | positive regulation of T cell activation | 5 | 0.0006113 |
| GO:0048020 | CCR chemokine receptor binding | 5 | 0.0006113 |
| GO:0045580 | regulation of T cell differentiation | 4 | 0.0006371 |
| GO:0000778 | condensed nuclear chromosome kinetochore | 4 | 0.0006371 |
| GO:0070233 | negative regulation of T cell apoptotic process | 4 | 0.0006371 |
| GO:0051315 | attachment of mitotic spindle microtubules to kinetochore | 4 | 0.0006371 |
| GO:0070365 | hepatocyte differentiation | 4 | 0.0006371 |
| GO:0043020 | NADPH oxidase complex | 4 | 0.0006371 |
| GO:0006909 | phagocytosis | 8 | 0.0006408 |
| GO:0044458 | motile cilium assembly | 6 | 0.0006779 |
| GO:0090023 | positive regulation of neutrophil chemotaxis | 6 | 0.0006779 |
| GO:0005930 | axoneme | 12 | 0.0007908 |
| GO:0043029 | T cell homeostasis | 7 | 0.0008503 |
| GO:0016538 | cyclin-dependent protein serine/threonine kinase regulator activity | 7 | 0.0008503 |
| GO:0030316 | osteoclast differentiation | 8 | 0.0008614 |
| GO:0007186 | G-protein coupled receptor signaling pathway | 48 | 0.0008614 |
| GO:0032753 | positive regulation of interleukin-4 production | 6 | 0.0010148 |
| GO:0021772 | olfactory bulb development | 6 | 0.0010148 |
| GO:0030595 | leukocyte chemotaxis | 5 | 0.0010173 |
| GO:0045503 | dynein light chain binding | 5 | 0.0010173 |
| GO:0050830 | defense response to Gram-positive bacterium | 10 | 0.0010354 |
| GO:0000307 | cyclin-dependent protein kinase holoenzyme complex | 8 | 0.0011228 |
| GO:0004896 | cytokine receptor activity | 8 | 0.0011228 |
| GO:0042110 | T cell activation | 7 | 0.0011593 |
| GO:0060294 | cilium movement involved in cell motility | 4 | 0.001287 |
| GO:0050707 | regulation of cytokine secretion | 4 | 0.001287 |
| GO:0030496 | midbody | 17 | 0.001336 |
| GO:0003700 | DNA-binding transcription factor activity | 65 | 0.0013598 |
| GO:0004888 | transmembrane signaling receptor activity | 25 | 0.0013995 |
| GO:0019957 | C-C chemokine binding | 6 | 0.0014737 |
| GO:0000070 | mitotic sister chromatid segregation | 6 | 0.0014737 |
| GO:0016887 | ATPase activity | 23 | 0.0015533 |
| GO:0032735 | positive regulation of interleukin-12 production | 5 | 0.0016348 |
| GO:0051301 | cell division | 10 | 0.0019328 |
| GO:0032729 | positive regulation of interferon-gamma production | 9 | 0.0019924 |
| GO:0048018 | receptor ligand activity | 6 | 0.0021079 |
| GO:0007159 | leukocyte cell-cell adhesion | 6 | 0.0021079 |
| GO:0005876 | spindle microtubule | 7 | 0.0021128 |
| GO:0000079 | regulation of cyclin-dependent protein serine/threonine kinase activity | 7 | 0.0021128 |
| GO:0030335 | positive regulation of cell migration | 23 | 0.0022611 |
| GO:0045086 | positive regulation of interleukin-2 biosynthetic process | 4 | 0.002342 |
| GO:0030502 | negative regulation of bone mineralization | 4 | 0.002342 |
| GO:0045060 | negative thymic T cell selection | 4 | 0.002342 |
| GO:0051382 | kinetochore assembly | 4 | 0.002342 |
| GO:0043548 | phosphatidylinositol 3-kinase binding | 4 | 0.002342 |
| GO:0005813 | centrosome | 43 | 0.0023557 |
| GO:0045505 | dynein intermediate chain binding | 5 | 0.0023557 |
| GO:0016021 | integral component of membrane | 290 | 0.0023557 |
| GO:0032715 | negative regulation of interleukin-6 production | 6 | 0.002653 |
| GO:0031018 | endocrine pancreas development | 6 | 0.002653 |
| GO:0007204 | positive regulation of cytosolic calcium ion concentration | 12 | 0.0032123 |
| GO:0016020 | membrane | 326 | 0.0032868 |
| GO:0002250 | adaptive immune response | 9 | 0.0033654 |
| GO:0050729 | positive regulation of inflammatory response | 9 | 0.0033654 |
| GO:0030041 | actin filament polymerization | 5 | 0.0033877 |
| GO:0034113 | heterotypic cell-cell adhesion | 5 | 0.0033877 |
| GO:0001530 | lipopolysaccharide binding | 6 | 0.0035827 |
| GO:0050776 | regulation of immune response | 6 | 0.0035827 |
| GO:0001891 | phagocytic cup | 4 | 0.0035827 |
| GO:0042832 | defense response to protozoan | 4 | 0.0035827 |
| GO:0048066 | developmental pigmentation | 4 | 0.0035827 |
| GO:2000484 | positive regulation of interleukin-8 secretion | 4 | 0.0035827 |
| GO:0043032 | positive regulation of macrophage activation | 4 | 0.0035827 |
| GO:0007019 | microtubule depolymerization | 4 | 0.0035827 |
| GO:0071346 | cellular response to interferon-gamma | 10 | 0.0036211 |
| GO:0031514 | motile cilium | 10 | 0.0036211 |
| GO:0000776 | kinetochore | 13 | 0.0046106 |
| GO:0051281 | positive regulation of release of sequestered calcium ion into cytosol | 6 | 0.0047572 |
| GO:0019882 | antigen processing and presentation | 5 | 0.0047723 |
| GO:0090102 | cochlea development | 5 | 0.0047723 |
| GO:0042531 | positive regulation of tyrosine phosphorylation of STAT protein | 9 | 0.0048159 |
| GO:0050852 | T cell receptor signaling pathway | 10 | 0.0050549 |
| GO:0004715 | non-membrane spanning protein tyrosine kinase activity | 8 | 0.0051906 |
| GO:0032760 | positive regulation of tumor necrosis factor production | 8 | 0.0051906 |
| GO:0071260 | cellular response to mechanical stimulus | 8 | 0.0051906 |
| GO:0007249 | I-kappaB kinase/NF-kappaB signaling | 7 | 0.005247 |
| GO:2000406 | positive regulation of T cell migration | 4 | 0.0055742 |
| GO:0036158 | outer dynein arm assembly | 4 | 0.0055742 |
| GO:0048247 | lymphocyte chemotaxis | 4 | 0.0055742 |
| GO:0021591 | ventricular system development | 4 | 0.0055742 |
| GO:0035589 | G-protein coupled purinergic nucleotide receptor signaling pathway | 4 | 0.0055742 |
| GO:0045028 | G-protein coupled purinergic nucleotide receptor activity | 4 | 0.0055742 |
| GO:0004869 | cysteine-type endopeptidase inhibitor activity | 5 | 0.0065626 |
| GO:0031295 | T cell costimulation | 5 | 0.0065626 |
| GO:0002548 | monocyte chemotaxis | 5 | 0.0065626 |
| GO:0004435 | phosphatidylinositol phospholipase C activity | 5 | 0.0065626 |
| GO:0006270 | DNA replication initiation | 5 | 0.0065626 |
| GO:0005125 | cytokine activity | 19 | 0.0068713 |
| GO:0048469 | cell maturation | 7 | 0.0076713 |
| GO:0090026 | positive regulation of monocyte chemotaxis | 4 | 0.0076713 |
| GO:0072687 | meiotic spindle | 4 | 0.0076713 |
| GO:0000777 | condensed chromosome kinetochore | 4 | 0.0076713 |
| GO:0098632 | cell-cell adhesion mediator activity | 4 | 0.0076713 |
| GO:0030031 | cell projection assembly | 4 | 0.0076713 |
| GO:0070527 | platelet aggregation | 5 | 0.0081494 |
| GO:1901224 | positive regulation of NIK/NF-kappaB signaling | 8 | 0.0083571 |
| GO:0005085 | guanyl-nucleotide exchange factor activity | 14 | 0.0085892 |
| GO:0046983 | protein dimerization activity | 22 | 0.0088555 |
| GO:0065003 | protein-containing complex assembly | 7 | 0.0091124 |
| GO:0051726 | regulation of cell cycle | 13 | 0.0096956 |
| GO:0070374 | positive regulation of ERK1 and ERK2 cascade | 21 | 0.0104846 |
| GO:0008277 | regulation of G-protein coupled receptor protein signaling pathway | 5 | 0.010857 |
| GO:0045582 | positive regulation of T cell differentiation | 4 | 0.0110644 |
| GO:0016601 | Rac protein signal transduction | 4 | 0.0110644 |
| GO:0051482 | positive regulation of cytosolic calcium ion concentration involved in phospholipase C-activating G-protein coupled signaling pathway | 4 | 0.0110644 |
| GO:0005576 | extracellular region | 58 | 0.0126964 |
| GO:0008584 | male gonad development | 10 | 0.0127681 |
| GO:0019221 | cytokine-mediated signaling pathway | 14 | 0.0129043 |
| GO:0000981 | RNA polymerase II transcription factor activity, sequence-specific DNA binding | 21 | 0.0129108 |
| GO:0043065 | positive regulation of apoptotic process | 23 | 0.0131728 |
| GO:0005886 | plasma membrane | 159 | 0.0131744 |
| GO:0032496 | response to lipopolysaccharide | 11 | 0.0132747 |
| GO:0030246 | carbohydrate binding | 12 | 0.013406 |
| GO:0072686 | mitotic spindle | 9 | 0.0134156 |
| GO:0050715 | positive regulation of cytokine secretion | 5 | 0.0138182 |
| GO:0050796 | regulation of insulin secretion | 6 | 0.0141461 |
| GO:0018108 | peptidyl-tyrosine phosphorylation | 14 | 0.0141622 |
| GO:0051607 | defense response to virus | 15 | 0.0148466 |
| GO:0090190 | positive regulation of branching involved in ureteric bud morphogenesis | 4 | 0.0150925 |
| GO:0008284 | positive regulation of cell proliferation | 35 | 0.0157348 |
| GO:0007229 | integrin-mediated signaling pathway | 10 | 0.0159105 |
| GO:0061098 | positive regulation of protein tyrosine kinase activity | 6 | 0.016428 |
| GO:0004930 | G-protein coupled receptor activity | 34 | 0.0166769 |
| GO:0032088 | negative regulation of NF-kappaB transcription factor activity | 9 | 0.0168121 |
| GO:0007411 | axon guidance | 15 | 0.0168319 |
| GO:0043406 | positive regulation of MAP kinase activity | 7 | 0.0172218 |
| GO:0032755 | positive regulation of interleukin-6 production | 7 | 0.0172218 |
| GO:0043011 | myeloid dendritic cell differentiation | 4 | 0.0172218 |
| GO:0030279 | negative regulation of ossification | 4 | 0.0172218 |
| GO:0071353 | cellular response to interleukin-4 | 4 | 0.0172218 |
| GO:0009612 | response to mechanical stimulus | 4 | 0.0172218 |
| GO:0009725 | response to hormone | 4 | 0.0172218 |
| GO:0043066 | negative regulation of apoptotic process | 36 | 0.0172218 |
| GO:0031663 | lipopolysaccharide-mediated signaling pathway | 6 | 0.0172218 |
| GO:0030168 | platelet activation | 6 | 0.0172218 |
| GO:0019955 | cytokine binding | 6 | 0.0172218 |
| GO:0050769 | positive regulation of neurogenesis | 5 | 0.0172218 |
| GO:0001819 | positive regulation of cytokine production | 5 | 0.0172218 |
| GO:0045499 | chemorepellent activity | 5 | 0.0172218 |
| GO:0007264 | small GTPase mediated signal transduction | 11 | 0.0172218 |
| GO:0030855 | epithelial cell differentiation | 8 | 0.0172218 |
| GO:0050728 | negative regulation of inflammatory response | 10 | 0.0174726 |
| GO:0048662 | negative regulation of smooth muscle cell proliferation | 4 | 0.0175489 |
| GO:0001782 | B cell homeostasis | 4 | 0.0175489 |
| GO:0031589 | cell-substrate adhesion | 4 | 0.0175489 |
| GO:0051959 | dynein light intermediate chain binding | 4 | 0.0175489 |
| GO:0045184 | establishment of protein localization | 5 | 0.0179923 |
| GO:0055088 | lipid homeostasis | 5 | 0.0179923 |
| GO:0001578 | microtubule bundle formation | 5 | 0.0179923 |
| GO:0050918 | positive chemotaxis | 5 | 0.0179923 |
| GO:0005104 | fibroblast growth factor receptor binding | 5 | 0.0179923 |
| GO:0007267 | cell-cell signaling | 7 | 0.0194137 |
| GO:0071526 | semaphorin-plexin signaling pathway | 6 | 0.0194137 |
| GO:0060070 | canonical Wnt signaling pathway | 9 | 0.0211982 |
| GO:0003281 | ventricular septum development | 5 | 0.0213557 |
| GO:0006805 | xenobiotic metabolic process | 4 | 0.0217715 |
| GO:0002102 | podosome | 4 | 0.0217715 |
| GO:0042056 | chemoattractant activity | 4 | 0.0217715 |
| GO:0005540 | hyaluronic acid binding | 4 | 0.0217715 |
| GO:0043325 | phosphatidylinositol-3,4-bisphosphate binding | 4 | 0.0217715 |
| GO:0001889 | liver development | 7 | 0.0220154 |
| GO:0050919 | negative chemotaxis | 6 | 0.0225498 |
| GO:0004713 | protein tyrosine kinase activity | 12 | 0.0234362 |
| GO:0016055 | Wnt signaling pathway | 11 | 0.0247308 |
| GO:0043280 | positive regulation of cysteine-type endopeptidase activity involved in apoptotic process | 5 | 0.0257248 |
| GO:0038083 | peptidyl-tyrosine autophosphorylation | 5 | 0.0257248 |
| GO:0050873 | brown fat cell differentiation | 5 | 0.0257248 |
| GO:0008305 | integrin complex | 5 | 0.0257248 |
| GO:0051092 | positive regulation of NF-kappaB transcription factor activity | 13 | 0.0258844 |
| GO:0001784 | phosphotyrosine residue binding | 6 | 0.026369 |
| GO:0005044 | scavenger receptor activity | 6 | 0.026369 |
| GO:0051965 | positive regulation of synapse assembly | 8 | 0.0264176 |
| GO:0043027 | cysteine-type endopeptidase inhibitor activity involved in apoptotic process | 4 | 0.027313 |
| GO:0000132 | establishment of mitotic spindle orientation | 4 | 0.027313 |
| GO:0051233 | spindle midzone | 4 | 0.027313 |
| GO:0043392 | negative regulation of DNA binding | 4 | 0.027313 |
| GO:0002053 | positive regulation of mesenchymal cell proliferation | 4 | 0.027313 |
| GO:0030215 | semaphorin receptor binding | 4 | 0.027313 |
| GO:0005615 | extracellular space | 69 | 0.0276932 |
| GO:0005543 | phospholipid binding | 10 | 0.0285106 |
| GO:0007417 | central nervous system development | 8 | 0.029814 |
| GO:0032720 | negative regulation of tumor necrosis factor production | 6 | 0.0301637 |
| GO:0005164 | tumor necrosis factor receptor binding | 5 | 0.0301637 |
| GO:0034260 | negative regulation of GTPase activity | 5 | 0.0301637 |
| GO:0015630 | microtubule cytoskeleton | 16 | 0.0301637 |
| GO:0007160 | cell-matrix adhesion | 9 | 0.0317091 |
| GO:0019722 | calcium-mediated signaling | 9 | 0.0317091 |
| GO:0051603 | proteolysis involved in cellular protein catabolic process | 7 | 0.0318341 |
| GO:0042476 | odontogenesis | 4 | 0.032777 |
| GO:0048872 | homeostasis of number of cells | 4 | 0.032777 |
| GO:0032757 | positive regulation of interleukin-8 production | 4 | 0.032777 |
| GO:0005178 | integrin binding | 10 | 0.0335087 |
| GO:0001657 | ureteric bud development | 6 | 0.0343385 |
| GO:0005814 | centriole | 11 | 0.0344476 |
| GO:0071300 | cellular response to retinoic acid | 5 | 0.0349944 |
| GO:0016358 | dendrite development | 5 | 0.0349944 |
| GO:0042169 | SH2 domain binding | 5 | 0.0349944 |
| GO:0044212 | transcription regulatory region DNA binding | 21 | 0.0349944 |
| GO:0045165 | cell fate commitment | 8 | 0.0349944 |
| GO:0043087 | regulation of GTPase activity | 10 | 0.0349944 |
| GO:0050731 | positive regulation of peptidyl-tyrosine phosphorylation | 9 | 0.0349944 |
| GO:0034446 | substrate adhesion-dependent cell spreading | 6 | 0.0349944 |
| GO:0008543 | fibroblast growth factor receptor signaling pathway | 6 | 0.0349944 |
| GO:0030218 | erythrocyte differentiation | 6 | 0.0349944 |
| GO:0097546 | ciliary base | 4 | 0.0349944 |
| GO:0042327 | positive regulation of phosphorylation | 4 | 0.0349944 |
| GO:0005070 | SH3/SH2 adaptor activity | 4 | 0.0349944 |
| GO:0045669 | positive regulation of osteoblast differentiation | 7 | 0.0349944 |
| GO:0005524 | ATP binding | 93 | 0.0349944 |
| GO:0043123 | positive regulation of I-kappaB kinase/NF-kappaB signaling | 12 | 0.0357142 |
| GO:0002244 | hematopoietic progenitor cell differentiation | 8 | 0.0359903 |
| GO:0030175 | filopodium | 6 | 0.0361769 |
| GO:0045121 | membrane raft | 13 | 0.0373343 |
| GO:0005096 | GTPase activator activity | 19 | 0.0384502 |
| GO:0034097 | response to cytokine | 5 | 0.0387087 |
| GO:0035019 | somatic stem cell population maintenance | 5 | 0.0387087 |
| GO:0005547 | phosphatidylinositol-3,4,5-trisphosphate binding | 4 | 0.0387087 |
| GO:0040008 | regulation of growth | 4 | 0.0387087 |
| GO:0001816 | cytokine production | 4 | 0.0387087 |
| GO:0007416 | synapse assembly | 4 | 0.0387087 |
| GO:0000793 | condensed chromosome | 4 | 0.0387087 |
| GO:0003148 | outflow tract septum morphogenesis | 4 | 0.0387087 |
| GO:0005911 | cell-cell junction | 15 | 0.0396675 |
| GO:0005215 | transporter activity | 8 | 0.0398766 |
| GO:0031532 | actin cytoskeleton reorganization | 7 | 0.0412582 |
| GO:0042742 | defense response to bacterium | 8 | 0.0440455 |
| GO:0001890 | placenta development | 5 | 0.0447136 |
| GO:0002062 | chondrocyte differentiation | 5 | 0.0447136 |
| GO:0005080 | protein kinase C binding | 5 | 0.0447136 |
| GO:0048661 | positive regulation of smooth muscle cell proliferation | 4 | 0.0460769 |
| GO:0048015 | phosphatidylinositol-mediated signaling | 4 | 0.0460769 |
| GO:0060976 | coronary vasculature development | 4 | 0.0460769 |
| GO:1902895 | positive regulation of pri-miRNA transcription by RNA polymerase II | 4 | 0.0460769 |
| GO:0048709 | oligodendrocyte differentiation | 4 | 0.0460769 |
| GO:0045766 | positive regulation of angiogenesis | 11 | 0.0487443 |

**Table S4. GO enrichment of the highly expressed genes in the SU compared to genes in the PU.**

| **id** | **term** | **ListHits** | **padj** |
| --- | --- | --- | --- |
| GO:0030018 | Z disc | 19 | 1.48E-10 |
| GO:0042383 | sarcolemma | 16 | 1.60E-10 |
| GO:0005925 | focal adhesion | 23 | 4.69E-09 |
| GO:0031012 | extracellular matrix | 30 | 6.28E-08 |
| GO:0003779 | actin binding | 32 | 9.15E-08 |
| GO:0001725 | stress fiber | 12 | 6.63E-07 |
| GO:0010881 | regulation of cardiac muscle contraction by regulation of the release of sequestered calcium ion | 4 | 3.17E-06 |
| GO:0006940 | regulation of smooth muscle contraction | 5 | 1.14E-05 |
| GO:0005509 | calcium ion binding | 48 | 4.33E-05 |
| GO:0008307 | structural constituent of muscle | 6 | 5.77E-05 |
| GO:0044325 | ion channel binding | 13 | 5.78E-05 |
| GO:0008076 | voltage-gated potassium channel complex | 12 | 5.81E-05 |
| GO:0070208 | protein heterotrimerization | 4 | 6.01E-05 |
| GO:0045214 | sarcomere organization | 8 | 7.11E-05 |
| GO:0005201 | extracellular matrix structural constituent | 10 | 0.00011118 |
| GO:0005581 | collagen trimer | 9 | 0.00011218 |
| GO:0043034 | costamere | 4 | 0.00015076 |
| GO:0030198 | extracellular matrix organization | 15 | 0.00015186 |
| GO:0050840 | extracellular matrix binding | 7 | 0.00017938 |
| GO:0043292 | contractile fiber | 4 | 0.00031462 |
| GO:0015629 | actin cytoskeleton | 19 | 0.00031482 |
| GO:0055114 | oxidation-reduction process | 43 | 0.00035673 |
| GO:0055085 | transmembrane transport | 40 | 0.00037509 |
| GO:0051015 | actin filament binding | 18 | 0.0005581 |
| GO:0008202 | steroid metabolic process | 6 | 0.00061451 |
| GO:0035725 | sodium ion transmembrane transport | 10 | 0.00062866 |
| GO:0016126 | sterol biosynthetic process | 5 | 0.00073711 |
| GO:0016020 | membrane | 242 | 0.00077519 |
| GO:0005978 | glycogen biosynthetic process | 4 | 0.00102194 |
| GO:0032148 | activation of protein kinase B activity | 5 | 0.0010667 |
| GO:0001968 | fibronectin binding | 5 | 0.0010667 |
| GO:0006816 | calcium ion transport | 12 | 0.00121628 |
| GO:0070588 | calcium ion transmembrane transport | 13 | 0.00124067 |
| GO:0005901 | caveola | 8 | 0.00125104 |
| GO:0004497 | monooxygenase activity | 8 | 0.00151764 |
| GO:0007229 | integrin-mediated signaling pathway | 10 | 0.00151764 |
| GO:0030425 | dendrite | 19 | 0.00170868 |
| GO:0007160 | cell-matrix adhesion | 10 | 0.00174108 |
| GO:0007015 | actin filament organization | 11 | 0.0019071 |
| GO:0030017 | sarcomere | 5 | 0.00195645 |
| GO:0042632 | cholesterol homeostasis | 9 | 0.00198853 |
| GO:0008092 | cytoskeletal protein binding | 10 | 0.00199622 |
| GO:0005216 | ion channel activity | 19 | 0.00214338 |
| GO:0007626 | locomotory behavior | 11 | 0.00214801 |
| GO:0005615 | extracellular space | 58 | 0.0023857 |
| GO:0005516 | calmodulin binding | 11 | 0.00243239 |
| GO:0005261 | cation channel activity | 5 | 0.0026242 |
| GO:0005251 | delayed rectifier potassium channel activity | 5 | 0.0026242 |
| GO:0006811 | ion transport | 29 | 0.00273281 |
| GO:0042734 | presynaptic membrane | 7 | 0.00305383 |
| GO:0060307 | regulation of ventricular cardiac muscle cell membrane repolarization | 4 | 0.00335639 |
| GO:0009986 | cell surface | 32 | 0.00363123 |
| GO:0030199 | collagen fibril organization | 7 | 0.00367628 |
| GO:0005249 | voltage-gated potassium channel activity | 9 | 0.0038049 |
| GO:0086091 | regulation of heart rate by cardiac conduction | 6 | 0.00407297 |
| GO:0008146 | sulfotransferase activity | 6 | 0.00407297 |
| GO:0010608 | posttranscriptional regulation of gene expression | 5 | 0.00417947 |
| GO:0014704 | intercalated disc | 5 | 0.00417947 |
| GO:0032332 | positive regulation of chondrocyte differentiation | 4 | 0.00448458 |
| GO:0042391 | regulation of membrane potential | 12 | 0.00466339 |
| GO:0005856 | cytoskeleton | 20 | 0.00473813 |
| GO:0060048 | cardiac muscle contraction | 6 | 0.00486999 |
| GO:0005178 | integrin binding | 10 | 0.00563278 |
| GO:0030170 | pyridoxal phosphate binding | 7 | 0.00569765 |
| GO:0004180 | carboxypeptidase activity | 4 | 0.00603695 |
| GO:0005248 | voltage-gated sodium channel activity | 4 | 0.00603695 |
| GO:0006874 | cellular calcium ion homeostasis | 10 | 0.00628744 |
| GO:0005886 | plasma membrane | 119 | 0.00702802 |
| GO:0005604 | basement membrane | 9 | 0.00707576 |
| GO:0071230 | cellular response to amino acid stimulus | 7 | 0.007756 |
| GO:0008021 | synaptic vesicle | 9 | 0.0078358 |
| GO:0030552 | cAMP binding | 4 | 0.0078358 |
| GO:0048566 | embryonic digestive tract development | 4 | 0.0078358 |
| GO:0098655 | cation transmembrane transport | 6 | 0.00802272 |
| GO:0043588 | skin development | 6 | 0.00802272 |
| GO:0005262 | calcium channel activity | 8 | 0.00819508 |
| GO:0050804 | modulation of chemical synaptic transmission | 5 | 0.00962787 |
| GO:0008305 | integrin complex | 5 | 0.00962787 |
| GO:0048675 | axon extension | 4 | 0.00999465 |
| GO:0016021 | integral component of membrane | 206 | 0.01004468 |
| GO:0005507 | copper ion binding | 6 | 0.01004468 |
| GO:0007616 | long-term memory | 4 | 0.01004468 |
| GO:0048786 | presynaptic active zone | 4 | 0.01004468 |
| GO:0071805 | potassium ion transmembrane transport | 12 | 0.01004468 |
| GO:0014069 | postsynaptic density | 13 | 0.01004468 |
| GO:0030036 | actin cytoskeleton organization | 12 | 0.01004468 |
| GO:0006813 | potassium ion transport | 11 | 0.01004468 |
| GO:0007613 | memory | 7 | 0.01004468 |
| GO:0030175 | filopodium | 6 | 0.01004468 |
| GO:0010595 | positive regulation of endothelial cell migration | 6 | 0.01004468 |
| GO:0070509 | calcium ion import | 4 | 0.01004468 |
| GO:0007612 | learning | 6 | 0.01083833 |
| GO:0009749 | response to glucose | 5 | 0.01227552 |
| GO:0052689 | carboxylic ester hydrolase activity | 4 | 0.01227552 |
| GO:0002027 | regulation of heart rate | 4 | 0.01227552 |
| GO:0080025 | phosphatidylinositol-3,5-bisphosphate binding | 4 | 0.01227552 |
| GO:0007519 | skeletal muscle tissue development | 6 | 0.01247093 |
| GO:0045211 | postsynaptic membrane | 9 | 0.01318677 |
| GO:0005913 | cell-cell adherens junction | 8 | 0.01426468 |
| GO:0031623 | receptor internalization | 5 | 0.0142705 |
| GO:0060291 | long-term synaptic potentiation | 5 | 0.0142705 |
| GO:0020037 | heme binding | 9 | 0.0142705 |
| GO:0016477 | cell migration | 14 | 0.01561512 |
| GO:0007155 | cell adhesion | 19 | 0.01569507 |
| GO:0045202 | synapse | 17 | 0.01652227 |
| GO:0043235 | receptor complex | 15 | 0.01788112 |
| GO:0016529 | sarcoplasmic reticulum | 4 | 0.01792021 |
| GO:0048709 | oligodendrocyte differentiation | 4 | 0.01792021 |
| GO:0030027 | lamellipodium | 11 | 0.01802818 |
| GO:0001938 | positive regulation of endothelial cell proliferation | 6 | 0.01815696 |
| GO:0006936 | muscle contraction | 5 | 0.01922907 |
| GO:0009898 | cytoplasmic side of plasma membrane | 5 | 0.01922907 |
| GO:0001649 | osteoblast differentiation | 7 | 0.01997366 |
| GO:0005882 | intermediate filament | 7 | 0.01997366 |
| GO:0002040 | sprouting angiogenesis | 4 | 0.02094389 |
| GO:0005244 | voltage-gated ion channel activity | 9 | 0.02098078 |
| GO:0010718 | positive regulation of epithelial to mesenchymal transition | 5 | 0.02098078 |
| GO:0008201 | heparin binding | 10 | 0.02098078 |
| GO:0034599 | cellular response to oxidative stress | 6 | 0.02098078 |
| GO:0016339 | calcium-dependent cell-cell adhesion via plasma membrane cell adhesion molecules | 4 | 0.02098078 |
| GO:0030879 | mammary gland development | 4 | 0.02098078 |
| GO:0035987 | endodermal cell differentiation | 4 | 0.02098078 |
| GO:0005887 | integral component of plasma membrane | 43 | 0.02098078 |
| GO:0005506 | iron ion binding | 10 | 0.02098078 |
| GO:0043209 | myelin sheath | 12 | 0.02098078 |
| GO:0001666 | response to hypoxia | 8 | 0.02313025 |
| GO:0051480 | regulation of cytosolic calcium ion concentration | 4 | 0.02334148 |
| GO:0060325 | face morphogenesis | 4 | 0.02334148 |
| GO:0007623 | circadian rhythm | 6 | 0.0248812 |
| GO:0008217 | regulation of blood pressure | 4 | 0.02697796 |
| GO:0009636 | response to toxic substance | 4 | 0.02697796 |
| GO:0062023 | collagen-containing extracellular matrix | 7 | 0.02796202 |
| GO:0016491 | oxidoreductase activity | 25 | 0.02803536 |
| GO:0010628 | positive regulation of gene expression | 23 | 0.02987568 |
| GO:0005267 | potassium channel activity | 7 | 0.03055781 |
| GO:0016358 | dendrite development | 4 | 0.03087563 |
| GO:0030178 | negative regulation of Wnt signaling pathway | 5 | 0.03167203 |
| GO:0021766 | hippocampus development | 5 | 0.03167203 |
| GO:0042593 | glucose homeostasis | 9 | 0.0330586 |
| GO:0051965 | positive regulation of synapse assembly | 6 | 0.0330586 |
| GO:0030335 | positive regulation of cell migration | 14 | 0.0330586 |
| GO:0005903 | brush border | 5 | 0.0330586 |
| GO:0097110 | scaffold protein binding | 5 | 0.0330586 |
| GO:0045296 | cadherin binding | 5 | 0.0330586 |
| GO:0031594 | neuromuscular junction | 5 | 0.0330586 |
| GO:0030334 | regulation of cell migration | 7 | 0.0330586 |
| GO:0050731 | positive regulation of peptidyl-tyrosine phosphorylation | 7 | 0.0330586 |
| GO:0006979 | response to oxidative stress | 8 | 0.03411805 |
| GO:0044297 | cell body | 5 | 0.03472004 |
| GO:0030900 | forebrain development | 6 | 0.03472004 |
| GO:0030168 | platelet activation | 4 | 0.03472004 |
| GO:0007043 | cell-cell junction assembly | 4 | 0.03472004 |
| GO:0015914 | phospholipid transport | 4 | 0.03472004 |
| GO:0004896 | cytokine receptor activity | 4 | 0.03472004 |
| GO:0001707 | mesoderm formation | 4 | 0.03472004 |
| GO:0010508 | positive regulation of autophagy | 4 | 0.03472004 |
| GO:0043491 | protein kinase B signaling | 4 | 0.03472004 |
| GO:0043154 | negative regulation of cysteine-type endopeptidase activity involved in apoptotic process | 5 | 0.03816391 |
| GO:0016705 | oxidoreductase activity, acting on paired donors, with incorporation or reduction of molecular oxygen | 5 | 0.03816391 |
| GO:0017124 | SH3 domain binding | 6 | 0.0418288 |
| GO:0009897 | external side of plasma membrane | 16 | 0.04284333 |
| GO:0016197 | endosomal transport | 4 | 0.04419731 |
| GO:0042995 | cell projection | 6 | 0.04488207 |
| GO:0008081 | phosphoric diester hydrolase activity | 5 | 0.04620695 |
| GO:0016459 | myosin complex | 4 | 0.04620695 |
| GO:0048812 | neuron projection morphogenesis | 5 | 0.04634468 |
| GO:0006897 | endocytosis | 7 | 0.04791452 |
| GO:0006898 | receptor-mediated endocytosis | 7 | 0.04791452 |
| GO:0005884 | actin filament | 6 | 0.04831255 |

**Table S5. KEGG enrichment of the highly expressed genes in the PU compared to genes in the SU.**

| **id** | **term** | **ListHits** | **padj** |
| --- | --- | --- | --- |
| path:ssc05150 | Staphylococcus aureus infection | 21 | 9.87E-13 |
| path:ssc04640 | Hematopoietic cell lineage | 28 | 2.23E-12 |
| path:ssc05332 | Graft-versus-host disease | 15 | 3.77E-09 |
| path:ssc04514 | Cell adhesion molecules (CAMs) | 31 | 3.77E-09 |
| path:ssc05330 | Allograft rejection | 15 | 1.34E-08 |
| path:ssc04062 | Chemokine signaling pathway | 33 | 8.34E-08 |
| path:ssc04658 | Th1 and Th2 cell differentiation | 22 | 8.34E-08 |
| path:ssc05340 | Primary immunodeficiency | 13 | 1.62E-07 |
| path:ssc05310 | Asthma | 11 | 2.20E-07 |
| path:ssc04659 | Th17 cell differentiation | 23 | 3.84E-07 |
| path:ssc04060 | Cytokine-cytokine receptor interaction | 40 | 5.16E-07 |
| path:ssc04672 | Intestinal immune network for IgA production | 14 | 6.38E-07 |
| path:ssc05140 | Leishmaniasis | 17 | 9.70E-07 |
| path:ssc04612 | Antigen processing and presentation | 16 | 1.12E-06 |
| path:ssc05320 | Autoimmune thyroid disease | 14 | 1.40E-06 |
| path:ssc04650 | Natural killer cell mediated cytotoxicity | 22 | 3.36E-06 |
| path:ssc04940 | Type I diabetes mellitus | 13 | 3.52E-06 |
| path:ssc05322 | Systemic lupus erythematosus | 20 | 4.57E-06 |
| path:ssc04660 | T cell receptor signaling pathway | 20 | 8.94E-06 |
| path:ssc05166 | Human T-cell leukemia virus 1 infection | 36 | 9.03E-06 |
| path:ssc05321 | Inflammatory bowel disease (IBD) | 15 | 9.70E-06 |
| path:ssc05416 | Viral myocarditis | 14 | 2.00E-05 |
| path:ssc05200 | Pathways in cancer | 57 | 2.79E-05 |
| path:ssc05169 | Epstein-Barr virus infection | 29 | 2.92E-05 |
| path:ssc05144 | Malaria | 12 | 5.00E-05 |
| path:ssc04110 | Cell cycle | 20 | 0.0001589 |
| path:ssc04620 | Toll-like receptor signaling pathway | 17 | 0.0001811 |
| path:ssc04666 | Fc gamma R-mediated phagocytosis | 15 | 0.0002967 |
| path:ssc05152 | Tuberculosis | 23 | 0.0004918 |
| path:ssc05142 | Chagas disease (American trypanosomiasis) | 17 | 0.0004979 |
| path:ssc05323 | Rheumatoid arthritis | 15 | 0.0004979 |
| path:ssc04380 | Osteoclast differentiation | 19 | 0.0006083 |
| path:ssc04662 | B cell receptor signaling pathway | 13 | 0.0006798 |
| path:ssc05162 | Measles | 19 | 0.0007209 |
| path:ssc04610 | Complement and coagulation cascades | 14 | 0.000784 |
| path:ssc04670 | Leukocyte transendothelial migration | 17 | 0.0010034 |
| path:ssc04015 | Rap1 signaling pathway | 26 | 0.0010034 |
| path:ssc05145 | Toxoplasmosis | 16 | 0.0013189 |
| path:ssc04145 | Phagosome | 20 | 0.0013189 |
| path:ssc05202 | Transcriptional misregulation in cancer | 22 | 0.0015684 |
| path:ssc00512 | Mucin type O-glycan biosynthesis | 7 | 0.0024107 |
| path:ssc04664 | Fc epsilon RI signaling pathway | 11 | 0.0031849 |
| path:ssc05226 | Gastric cancer | 19 | 0.0046514 |
| path:ssc04390 | Hippo signaling pathway | 19 | 0.0058845 |
| path:ssc04064 | NF-kappa B signaling pathway | 13 | 0.0082616 |
| path:ssc05133 | Pertussis | 11 | 0.0097687 |
| path:ssc05221 | Acute myeloid leukemia | 10 | 0.0101971 |
| path:ssc05205 | Proteoglycans in cancer | 22 | 0.0109314 |
| path:ssc04934 | Cushing syndrome | 18 | 0.0119084 |
| path:ssc04914 | Progesterone-mediated oocyte maturation | 12 | 0.0157703 |
| path:ssc05143 | African trypanosomiasis | 6 | 0.0185352 |
| path:ssc05020 | Prion diseases | 6 | 0.0185352 |
| path:ssc05224 | Breast cancer | 17 | 0.0190919 |
| path:ssc04550 | Signaling pathways regulating pluripotency of stem cells | 16 | 0.0190919 |
| path:ssc05217 | Basal cell carcinoma | 9 | 0.0251766 |
| path:ssc04360 | Axon guidance | 19 | 0.0288737 |
| path:ssc05164 | Influenza A | 17 | 0.0308031 |
| path:ssc04068 | FoxO signaling pathway | 15 | 0.0308031 |
| path:ssc04014 | Ras signaling pathway | 23 | 0.0319259 |
| path:ssc04916 | Melanogenesis | 12 | 0.0363238 |
| path:ssc04151 | PI3K-Akt signaling pathway | 31 | 0.0434824 |
| path:ssc05168 | Herpes simplex infection | 18 | 0.0451056 |

**Table S6. KEGG enrichment of the highly expressed genes in the SU compared to genes in the PU.**

| **id** | **term** | **ListHits** | **padj** |
| --- | --- | --- | --- |
| path:ssc04510 | Focal adhesion | 36 | 4.50E-12 |
| path:ssc04512 | ECM-receptor interaction | 20 | 2.34E-09 |
| path:ssc04974 | Protein digestion and absorption | 18 | 3.50E-07 |
| path:ssc05414 | Dilated cardiomyopathy (DCM) | 16 | 4.64E-06 |
| path:ssc04270 | Vascular smooth muscle contraction | 20 | 4.64E-06 |
| path:ssc00360 | Phenylalanine metabolism | 8 | 4.64E-06 |
| path:ssc00100 | Steroid biosynthesis | 7 | 2.47E-05 |
| path:ssc04611 | Platelet activation | 17 | 0.0001463 |
| path:ssc00260 | Glycine, serine and threonine metabolism | 9 | 0.0001463 |
| path:ssc05412 | Arrhythmogenic right ventricular cardiomyopathy | 12 | 0.0001485 |
| path:ssc05410 | Hypertrophic cardiomyopathy (HCM) | 13 | 0.0001764 |
| path:ssc04022 | cGMP-PKG signaling pathway | 20 | 0.0001764 |
| path:ssc00350 | Tyrosine metabolism | 8 | 0.0002968 |
| path:ssc04713 | Circadian entrainment | 13 | 0.0010849 |
| path:ssc04810 | Regulation of actin cytoskeleton | 21 | 0.0016853 |
| path:ssc04020 | Calcium signaling pathway | 19 | 0.0025559 |
| path:ssc04913 | Ovarian steroidogenesis | 8 | 0.0027403 |
| path:ssc04015 | Rap1 signaling pathway | 20 | 0.0033909 |
| path:ssc00410 | beta-Alanine metabolism | 6 | 0.0044822 |
| path:ssc04151 | PI3K-Akt signaling pathway | 28 | 0.0048586 |
| path:ssc00250 | Alanine, aspartate and glutamate metabolism | 6 | 0.0106442 |
| path:ssc04730 | Long-term depression | 8 | 0.0106476 |
| path:ssc04261 | Adrenergic signaling in cardiomyocytes | 14 | 0.0106476 |
| path:ssc00330 | Arginine and proline metabolism | 7 | 0.0114966 |
| path:ssc04727 | GABAergic synapse | 10 | 0.0114966 |
| path:ssc04721 | Synaptic vesicle cycle | 8 | 0.013232 |
| path:ssc04978 | Mineral absorption | 6 | 0.0136132 |
| path:ssc00590 | Arachidonic acid metabolism | 8 | 0.013827 |
| path:ssc04926 | Relaxin signaling pathway | 13 | 0.0144982 |
| path:ssc05146 | Amoebiasis | 10 | 0.0202517 |
| path:ssc00400 | Phenylalanine, tyrosine and tryptophan biosynthesis | 2 | 0.0222236 |
| path:ssc00340 | Histidine metabolism | 4 | 0.0233909 |
| path:ssc00790 | Folate biosynthesis | 4 | 0.0270255 |
| path:ssc04726 | Serotonergic synapse | 11 | 0.0270255 |
| path:ssc04921 | Oxytocin signaling pathway | 13 | 0.0401516 |
| path:ssc00072 | Synthesis and degradation of ketone bodies | 2 | 0.0429896 |
| path:ssc00140 | Steroid hormone biosynthesis | 6 | 0.0471405 |
| path:ssc05031 | Amphetamine addiction | 7 | 0.0471405 |

**Table S7. The gene expressions in the mucosal epithelial cells of endometrium during the proliferative and secretory phases.**

| **FeatureName** | **EC-PU Average** | **EC-SU Average** |
| --- | --- | --- |
| COL1A1 | 0.007249551 | 1.227601422 |
| COL1A2 | 0.00859206 | 1.120202232 |
| UPTI | 0.005101536 | 48.30892044 |
| RBP4 | 0.006175543 | 41.87261766 |
| PHYH | 3.517911643 | 0.528709958 |
| VIM | 0.049404346 | 6.52712999 |
| ID4 | 1.053735631 | 0.114410413 |
| PHGDH | 2.124655383 | 0.645988599 |
| PRSS23 | 0.002953521 | 1.690023453 |
| LAMA3 | 0.008189307 | 4.788027976 |
| CENPF | 1.128513405 | 0.008285991 |
| LCN2 | 1.981946634 | 0.124608556 |
| LOC100739218 | 0.000268502 | 1.497852202 |
| SOD3 | 0.014767603 | 3.102784902 |
| TSTD1 | 1.393927515 | 0.793224283 |
| NMU | 0.044839814 | 8.668102563 |
| MAP1B | 0.011545581 | 1.745794545 |
| TIMP1 | 0.089411126 | 2.084564097 |
| CD2AP | 1.105153742 | 0.312318119 |
| PLS1 | 2.433701048 | 1.645087887 |
| LOC100524999 | 0.012485337 | 1.016308654 |
| MDK | 5.599472475 | 3.13337933 |
| KRT8 | 4.419540959 | 3.267548644 |
| CFD | 0.201376411 | 12.37066573 |
| SOX17 | 1.132809435 | 0.747013949 |
| ATP13A3 | 1.518646638 | 0.16444505 |
| KRT18 | 1.625778889 | 1.298669728 |
| CDO1 | 0.003490524 | 1.019176881 |
| MGST1 | 2.64662304 | 1.366232424 |
| FOLR1 | 0.223393565 | 1.210073364 |
| SPP1 | 0.039738278 | 2.328682136 |
| TST | 1.514082106 | 1.353484745 |
| COL17A1 | 0.019063634 | 1.397145543 |
| STMN1 | 3.061995449 | 0.492697767 |
| S100A10 | 0.337104111 | 5.428280119 |
| LOC100626247 | 2.277835706 | 0.647263367 |
| MRPS26 | 1.79305556 | 0.772509306 |
| TIMP2 | 0.264877105 | 5.98184805 |
| S100A1 | 1.342912157 | 1.132631218 |
| CLDN4 | 3.333585102 | 3.90652602 |
| TSPAN1 | 1.417824182 | 1.537051313 |
| UPP1 | 0.037858765 | 1.655286029 |
| SLC12A2 | 0.03423399 | 1.507412961 |
| MMP7 | 0.048733091 | 2.730871387 |
| CLDN7 | 1.454071936 | 1.910239596 |
| ISG12(A) | 7.875562919 | 6.435665398 |
| LOC110260659 | 0.535661252 | 1.426783896 |
| IGFBP2 | 1.332709086 | 0.955119798 |
| PRNP | 0.054640133 | 1.067618059 |
| MECOM | 0.998558495 | 1.116696621 |
| SLPI | 2.482299889 | 0.559623078 |
| PSAP | 0.389998982 | 4.692101696 |
| CRIM1 | 0.087665864 | 1.848732048 |
| HMGN1 | 2.27407668 | 1.020451649 |
| H2AFZ | 2.054844894 | 0.347374234 |
| ITGA2 | 0.046450825 | 1.383441789 |
| KRT7 | 0.81087568 | 1.207205137 |
| DDT | 1.254038035 | 0.504808061 |
| CD9 | 2.830278326 | 3.548316259 |
| NUCKS1 | 3.482872148 | 0.776333609 |
| ATP11A | 0.065111706 | 1.000692748 |
| PRDX2 | 2.295691081 | 1.293889349 |
| CTSC | 0.21265349 | 1.327033313 |
| TMEM238 | 1.185570055 | 1.484785832 |
| ZFP36L2 | 0.521699155 | 1.102355482 |
| CD74 | 1.429638265 | 0.094014128 |
| KRT19 | 1.941939853 | 3.416377789 |
| UNC5CL | 0.069139234 | 1.556491522 |
| LOC100512448 | 2.332207337 | 0.338769551 |
| SPINT2 | 2.477064102 | 3.683760341 |
| WFDC2 | 1.243566461 | 2.214590416 |
| ATP1A1 | 2.529421969 | 3.089081148 |
| TACSTD2 | 1.086492861 | 1.924262042 |
| HMGN2 | 4.068071997 | 0.670527879 |
| DBI | 2.443769869 | 0.860149594 |
| SOX4 | 1.751035016 | 1.722211341 |
| IFITM1 | 0.586810861 | 1.367825883 |
| CD320 | 0.966338269 | 1.716156193 |
| ACP5 | 0.04000678 | 1.158763959 |
| PTPRF | 0.938548325 | 1.444949337 |
| DYNLL1 | 1.421583208 | 0.950020727 |
| SLC46A2 | 0.058801912 | 1.144741513 |
| HMGB1 | 6.179570788 | 1.767146907 |
| SNRPG | 1.867967585 | 0.861424362 |
| GPX1 | 0.446115875 | 1.02714418 |
| CDH1 | 0.802015118 | 1.336594071 |
| RN18S | 13.48094243 | 30.12754424 |
| CLU | 1.536233511 | 1.006429203 |
| DDIT4 | 0.428529002 | 1.292295889 |
| ERH | 1.362110042 | 0.631010077 |
| SNRPE | 1.26477811 | 0.618899782 |
| ID3 | 1.473001318 | 0.083178601 |
| LOC102158335 | 1.057226156 | 8.522141647 |
| RPSA | 9.969743339 | 3.429444159 |
| LGALS1 | 1.062327692 | 2.434487866 |
| ATP5G3 | 2.268438141 | 1.22887619 |
| PSMB6 | 1.008493065 | 0.455410808 |
| RPS2 | 20.67330232 | 8.177954332 |
| LOC102157763 | 2.214737764 | 0.119509484 |
| GLB1 | 0.083504085 | 1.006429203 |

**Table S8. Differences of the colonialized luminal bacteria in the PU and SU.**

| **taxa** | **mean(PU)** | **mean(SU)** | **p-value** |
| --- | --- | --- | --- |
| Bacteroides.zoogleoformans | 0 | 0.001223 | 0.000999 |
| Caloramator.australicus | 0 | 0.00195 | 0.000999 |
| Caloramator.coolhaasii | 0 | 0.000625 | 0.000999 |
| Hydrogenophilus.hirschii | 0 | 0.006707 | 0.000999 |
| Meiothermus.ruber | 0 | 0.008293 | 0.000999 |
| Prevotella.brevis | 0.000178 | 0.003034 | 0.000999 |
| Sporanaerobacter.acetigenes | 0 | 0.000984 | 0.000999 |
| Tepidimonas.taiwanensis | 0 | 0.00266 | 0.000999 |
| Tepidiphilus.margaritifer | 0 | 0.000741 | 0.000999 |
| Thermus.igniterrae | 0 | 0.000925 | 0.000999 |
| Turicibacter.sanguinis | 0 | 0.000556 | 0.000999 |
| Ureibacillus.thermophilus | 0 | 0.002788 | 0.000999 |
| Parabacteroides.distasonis | 0.000362 | 0.002309 | 0.001998 |
| Prevotella.buccalis | 0.000684 | 0.003691 | 0.001998 |
| Curvibacter.gracilis | 0.000407 | 0 | 0.002893 |
| Deinococcus.wulumuqiensis | 0 | 0.000495 | 0.002997 |
| Mitsuokella.jalaludinii | 0.000534 | 0.003895 | 0.002997 |
| Paraprevotella.clara | 0.000097 | 0.003529 | 0.002997 |
| Streptococcus.alactolyticus | 0.000921 | 0.004549 | 0.002997 |
| Fluviicola.taffensis | 0 | 0.00041 | 0.003701 |
| Sphingobium.qiguonii | 0 | 0.000408 | 0.003701 |
| Ureibacillus.thermosphaericus | 0 | 0.000407 | 0.003701 |
| Prevotella.copri | 0.002492 | 0.009433 | 0.003996 |
| Pseudomonas.oryzihabitans | 0.000397 | 0 | 0.005392 |
| Tepidimicrobium.ferriphilum | 0 | 0.006324 | 0.005994 |
| Christensenella.minuta | 0 | 0.000328 | 0.008237 |
| Grimontia.hollisae | 0 | 0.000333 | 0.008237 |
| Novosphingobium.stygium | 0 | 0.000325 | 0.008237 |
| Paracoccus.aminophilus | 0 | 0.000332 | 0.008237 |
| Prevotella.buccae | 0 | 0.000334 | 0.008237 |
| Prevotella.melaninogenica | 0 | 0.000331 | 0.008237 |
| Selenomonas.lacticifex | 0 | 0.00032 | 0.008237 |
| Acetobacterium.wieringae | 0.000367 | 0 | 0.010101 |
| Bacillus.flexus | 0.00035 | 0 | 0.010101 |
| Butyricimonas.paravirosa | 0.000358 | 0 | 0.010101 |
| Chryseobacterium.polytrichastri | 0.000368 | 0 | 0.010101 |
| Caloramator.indicus | 0 | 0.000574 | 0.010989 |
| Proteocatella.sphenisci | 0.00183 | 0.008684 | 0.013986 |
| Stenotrophomonas.koreensis | 0.000265 | 0.002432 | 0.014985 |
| Psychrobacter.maritimus | 0.000625 | 0.003768 | 0.017982 |
| Acidovorax.caeni | 0 | 0.000275 | 0.018331 |
| Anaerococcus.obesiensis | 0 | 0.000276 | 0.018331 |
| Carnobacterium.alterfunditum | 0 | 0.000263 | 0.018331 |
| Deinococcus.caeni | 0 | 0.000263 | 0.018331 |
| Eubacterium.ramulus | 0 | 0.000295 | 0.018331 |
| Iamia.majanohamensis | 0 | 0.000263 | 0.018331 |
| Lysobacter.concretionis | 0 | 0.000292 | 0.018331 |
| Paenarthrobacter.nitroguajacolicus | 0 | 0.000295 | 0.018331 |
| Prevotella.amnii | 0 | 0.000278 | 0.018331 |
| Pseudoxanthomonas.taiwanensis | 0 | 0.000291 | 0.018331 |
| Selenomonas.noxia | 0 | 0.000278 | 0.018331 |
| Sutterella.stercoricanis | 0 | 0.000276 | 0.018331 |
| Terrimonas.rubra | 0 | 0.000295 | 0.018331 |
| Kluyvera.intermedia | 0.03962 | 0.029029 | 0.018981 |
| Ochrobactrum.anthropi | 0.258704 | 0.19457 | 0.018981 |
| Burkholderia.rinojensis | 0.000318 | 0 | 0.01903 |
| Dysgonomonas.capnocytophagoides | 0.000325 | 0 | 0.01903 |
| Moheibacter.sediminis | 0.000318 | 0 | 0.01903 |
| Nitrosospira.multiformis | 0.000308 | 0 | 0.01903 |
| Psychrobacter.arenosus | 0.000331 | 0 | 0.01903 |
| Deferrisoma.camini | 0.000491 | 0.000053 | 0.022977 |
| Actinomyces.nasicola | 0.000044 | 0.000409 | 0.026407 |
| Blautia.producta | 0.000043 | 0.000354 | 0.026407 |
| Mucilaginibacter.herbaticus | 0.000045 | 0.000409 | 0.026407 |
| Prevotella.timonensis | 0.000043 | 0.000354 | 0.026407 |
| Pseudoxanthomonas.mexicana | 0.000043 | 0.000373 | 0.026407 |
| Sphingomonas.phyllosphaerae | 0.000484 | 0.000053 | 0.026973 |
| Bacteroides.xylanisolvens | 0.000383 | 0.000059 | 0.027889 |
| Acidovorax.facilis | 0.000467 | 0.000055 | 0.028701 |
| Novosphingobium.sediminis | 0.000471 | 0.000053 | 0.028701 |
| Robinsoniella.peoriensis | 0.000446 | 0.000053 | 0.028701 |
| Serratia.proteamaculans | 0.029161 | 0.020422 | 0.02997 |
| Bacteroides.coprophilus | 0.000045 | 0.001056 | 0.030969 |
| Amycolatopsis.bartoniae | 0.008094 | 0.005329 | 0.033966 |
| Anaerococcus.octavius | 0.000255 | 0 | 0.036082 |
| Chryseobacterium.hominis | 0.000263 | 0 | 0.036082 |
| [Eubacterium].hallii | 0.000287 | 0 | 0.036082 |
| Methylobacterium.radiotolerans | 0.000266 | 0 | 0.036082 |
| Microbacterium.proteolyticum | 0.000284 | 0 | 0.036082 |
| Ruminococcus.flavefaciens | 0.000263 | 0 | 0.036082 |
| Sphingomonas.hankookensis | 0.000256 | 0 | 0.036082 |
| Acinetobacter.junii | 0.000828 | 0.000262 | 0.036963 |
| Megasphaera.elsdenii | 0.000196 | 0.00132 | 0.036963 |
| Tepidiphilus.succinatimandens | 0 | 0.001037 | 0.03996 |
| Acetoanaerobium.sticklandii | 0 | 0.000218 | 0.040792 |
| Actinoallomurus.yoronensis | 0 | 0.000233 | 0.040792 |
| Anaeromyxobacter.dehalogenans | 0 | 0.000236 | 0.040792 |
| Anaerorhabdus.furcosa | 0 | 0.000202 | 0.040792 |
| Bacteroides.clarus | 0 | 0.000226 | 0.040792 |
| Barnesiella.viscericola | 0 | 0.00023 | 0.040792 |
| Citricoccus.zhacaiensis | 0 | 0.000219 | 0.040792 |
| Cronobacter.turicensis | 0 | 0.000228 | 0.040792 |
| Empedobacter.brevis | 0 | 0.000213 | 0.040792 |
| Fervidobacterium.nodosum | 0 | 0.000233 | 0.040792 |
| Gemmata.obscuriglobus | 0 | 0.000236 | 0.040792 |
| Gemmobacter.aquaticus | 0 | 0.000233 | 0.040792 |
| Hydrogenophaga.taeniospiralis | 0 | 0.000205 | 0.040792 |
| Luteimonas.abyssi | 0 | 0.000224 | 0.040792 |
| Lysobacter.novalis | 0 | 0.000236 | 0.040792 |
| Methylobacterium.dankookense | 0 | 0.000207 | 0.040792 |
| Natronincola.histidinovorans | 0 | 0.000215 | 0.040792 |
| Prevotella.histicola | 0 | 0.000223 | 0.040792 |
| Prevotella.jejuni | 0 | 0.000224 | 0.040792 |
| Sphingobacterium.kitahiroshimense | 0 | 0.000206 | 0.040792 |
| Pseudomonas.proteolytica | 0.008762 | 0.005692 | 0.040959 |
| Acinetobacter.guillouiae | 0.121198 | 0.089868 | 0.041958 |
| Thermanaerovibrio.acidaminovorans | 0 | 0.003136 | 0.043956 |
| Sphingomonas.mali | 0.000583 | 0.000164 | 0.044955 |
| Ottowia.pentelensis | 0.000044 | 0.000509 | 0.045954 |
| Casaltella.massiliensis | 0.000353 | 0.000058 | 0.047774 |
| Holdemanella.biformis | 0.00037 | 0.000059 | 0.047774 |
| Massilia.arvi | 0.000376 | 0.000059 | 0.047774 |
| Beijerinckia.fluminensis | 0.024995 | 0.018501 | 0.048951 |
| Succinivibrio.dextrinosolvens | 0.000285 | 0.001672 | 0.048951 |

**Table S9. The primers used to detect the genes in the UMEC and lymphocytes.**

| **Gene name** | **Sequence (5'-3')** | | **Length** |
| --- | --- | --- | --- |
| *CCL5* | Forward | CTCCCCATATGCCTCGGACA | 167 bp |
|  | Reverse | TCTTCTCTGGGTTGGCACAC |  |
| *CCR1* | Forward | AAAGCCCACTGCCTTTAGCA | 153 bp |
|  | Reverse | CTCCTGTACCTTCTGGCACG |  |
| *CCL4* | Forward | ATGAAGCTCTGCGTGACTGT | 147 bp |
|  | Reverse | AGTCACGAAGTTGCGAGGAA |  |
| *TLR2* | Forward | ACGTATCCATCAATGAACACTGC | 153 bp |
|  | Reverse | GTCCGTTAAGGGTGCAGTCA |  |
| *CX3CR1* | Forward | AGGAGGGAGGGAAAGGGAAA | 111 bp |
|  | Reverse | GACATGGTGAGTGCCTGGG |  |
| *CX3CL1* | Forward | GTCGTCATCTTGAAGACCAGAAA | 151 bp |
|  | Reverse | GCTCACTCATGCCGATTTGC |  |
| *GAPDH* | Forward | GTCGGAGTGAACGGATTTGGC | 150 bp |
|  | Reverse | CTTGCCGTGGGTGGAATCAT |  |
